# Studying the Intrinsic Reactivity of Chromanes by Gas-Phase Infrared Spectroscopy

**DOI:** 10.1021/jasms.4c00216

**Published:** 2024-07-01

**Authors:** Carla Kirschbaum, Kim Greis, América Y. Torres-Boy, Jerome Riedel, Sandy Gewinner, Wieland Schöllkopf, Gerard Meijer, Gert von Helden, Kevin Pagel

**Affiliations:** † Freie Universität Berlin, Institute of Chemistry and Biochemistry, 14195 Berlin, Germany; ‡ Fritz Haber Institute of the Max Planck Society, 14195 Berlin, Germany

## Abstract

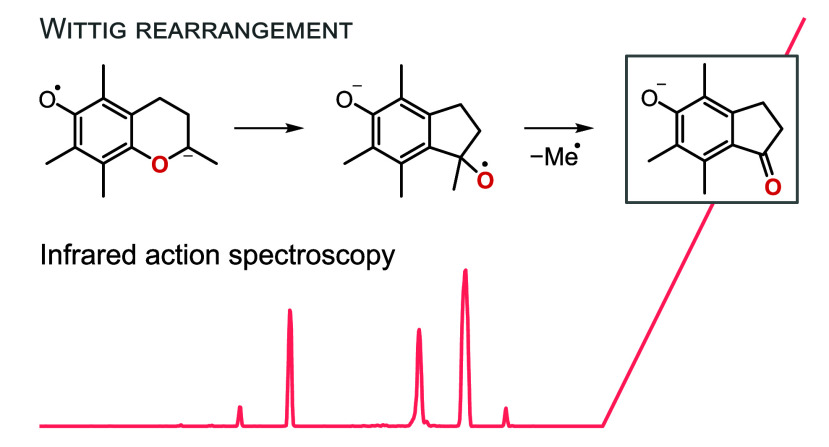

Tandem mass spectrometry is routinely used for the structural analysis of organic molecules, but many fragmentation reactions are not well understood. Because several potential structures can correspond to a measured mass, the assignment of product ions is ambiguous using mass spectrometry alone. Here, we combine mass spectrometry with high-resolution gas-phase infrared spectroscopy and computational chemistry tools to identify product ion structures and derive collision-induced fragmentation mechanisms of the chromane derivatives Trolox and Methyltrolox. We find that protonated Trolox and Methyltrolox fragment identically via dehydration and decarbonylation, while deprotonated ions display substantially diverging reactivities. For deprotonated Methyltrolox, we observe unusual radical fragmentation reactions and suggest a [1,2]-Wittig rearrangement involving aryl migration in the gas phase. Overall, the combined experimental and theoretical approach presented here revealed complex proton dynamics and intramolecular rearrangement reactions, which expand our understanding on structure–reactivity relationships of isolated molecules in different protonation states.

## Introduction

Mass spectrometry (MS) is one of the main techniques used for the structural analysis of organic molecules.^1^ Structural information is obtained using collision-, electron-, or photon-induced fragmentation of analyte ions in tandem mass spectrometry (MS^2^) experiments.^2^ However, the fragmentation processes occurring inside the mass spectrometer are challenging to predict by quantum chemistry,^3^ and a recent study showed that the majority of fragment ion structure annotations in computationally generated MS^2^ spectra are incorrect.^4^ Gaining fundamental understanding of the correlations between ion structure and dissociation behavior could help to improve the rule-based prediction of fragmentation patterns^5,6^ and to validate quantum chemical or machine learning-based methods that generate MS^2^ spectra *in silico*.^3,7,8^ Furthermore, knowledge of fragmentation mechanisms greatly facilitates the interpretation of MS^2^ spectra and increases the gain in structural information.^9^

To unravel fragmentation mechanisms, the fragment structures resulting from ion activation must be experimentally confirmed, which is challenging because fragments are generated *in situ* and their lifetime is restricted to their travel through the mass spectrometer. A classical MS^2^ experiment merely yields a list of mass-to-charge ratios (*m*/*z*) corresponding to fragment ions, the formation of which can further be investigated using energy-resolved measurements.^10^ In most cases, these fragment ions can correspond to a large number of conceivable structures. Structural information on gas-phase ions can be obtained, for example, using ion–molecule reactions that allow to identify functional groups.^11−13^ Another technique that has been extensively used to determine structures of intact ions and dissociation products is gas-phase infrared (IR) ion spectroscopy.^4,14−20^ Gas-phase IR spectroscopy yields IR spectra of *m*/*z*-selected ions, which are characteristic of the ionic structure and reveal the presence of various functional groups. Because IR absorption of isolated ions in the gas phase reflects their quantum-chemical properties, the probed ion structures can be assigned by comparison with quantum-chemically computed IR spectra of candidate structures. The comparison between calculated and experimental IR spectra is essential for obtaining detailed structural information and assigning structures.^21^

Additional certainty about the fragment structure can be gained by orthogonal gas-phase techniques that can be coupled to MS, such as ion mobility-mass spectrometry (IM-MS).^22^ Drift-tube IM-MS provides the collision cross section (CCS) of fragment ions as an additional structural parameter that can be used, for example, to distinguish between compact and extended structures. There are a number of approaches to theoretically calculate the CCS candidate structures for comparison with the experimental value.^23−25^

**Figure 1:**
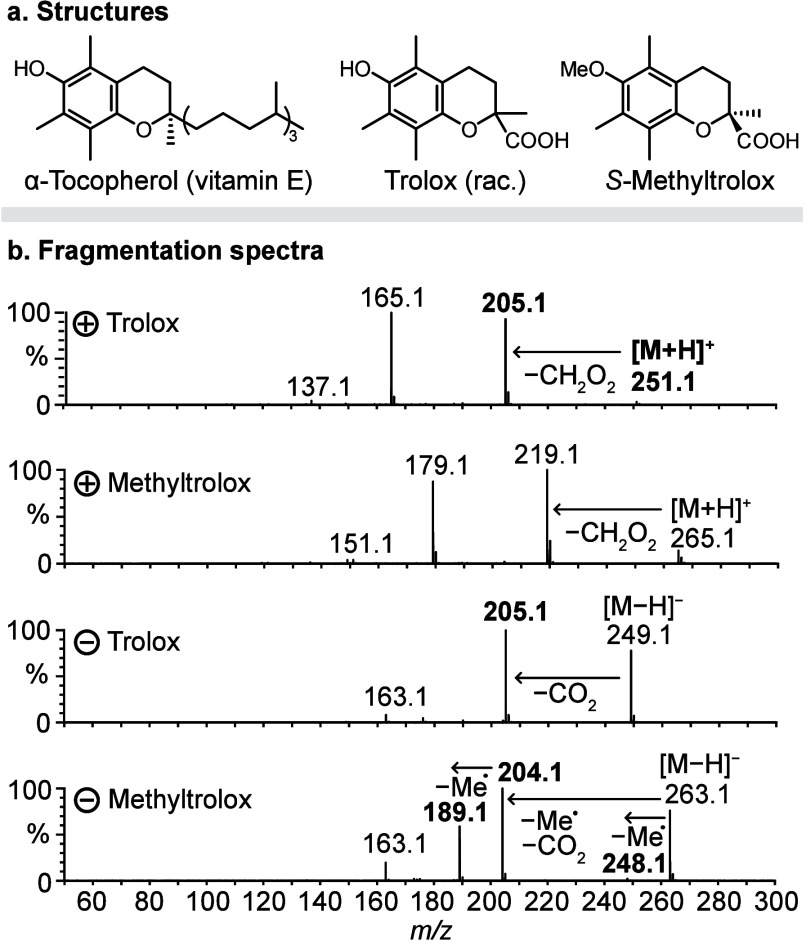
Collision-induced dissociation of Trolox and Methyltrolox. (a) Chemical structures of α-tocopherol, Trolox and *S*-Methyltrolox. (b) MS/MS spectra of protonated and deprotonated Trolox and Methyltrolox. The ions highlighted in bold have been investigated by infrared spectroscopy in this work.

Of particular interest for the understanding of fragmentation reactions is how the charge and availability of charge carriers influence the fragmentation behavior. Here we focus on the influence of the protonation state and availability of charge-stabilizing functional groups on the fragmentation of small organic molecules. As a suitable system to investigate this we selected Trolox and Methyltrolox–two chromane-derived antioxidants and water-soluble analogs of vitamin E (Figure 1a).^26^ Depending on their protonation state, Trolox and Methyltrolox undergo substantially different fragmentation reactions (Figure 1b), which makes them an interesting target to study the influence of protons on fragmentation. In addition, the two molecules differ only by a methyl group attached to the phenol oxygen in Methyltrolox, as a consequence of which the oxygen loses its ability to stabilize a negative charge. Therefore, this pair of molecules also offers the possibility of investigating the influence of a small structural difference on the fragmentation behavior of deprotonated ions.

Here, we investigate collision-induced fragmentation reactions of protonated and deprotonated Trolox and Methyltrolox using gas-phase IR spectroscopy in combination with drift-tube IM-MS and different computational chemistry tools. We show that reaction products can be unambiguously identified by matching experimental IR spectra with computed spectra of candidate structures. Based on the confirmed fragment structures, we suggest computed fragmentation reaction mechanisms. We identified several unexpected reactions including radical fragmentation and, most interestingly, a [1,2]-Wittig rearrangement of an aryl ether.

## Experimental Section

### Sample Preparation

(±)-6-Hydroxy-2,5,7,8-tetramethylchromane-2-carboxylic acid (Trolox) and (S)-6-Methoxy-2,5,7,8-tetramethylchromane-2-carboxylic acid (Methyltrolox) were purchased from Sigma-Aldrich (Taufkirchen, Germany). Stock solutions (10 mM) were prepared in methanol (HPLC grade, Sigma-Aldrich) and diluted to 50–200 μM before measurement. The solutions were stored at −25 °C until use.

### Cryogenic Infrared Ion Spectroscopy in Superfluid Helium Droplets

Gas-phase IR spectra were measured on a custom-built instrument described previously.^27,28^ Protonated and deprotonated ions were generated from methanolic solutions of Trolox or Methyltrolox (100–200 μM) by nanoelectrospray ionization. Emitter tips were prepared in-house from borosilicate capillaries pulled by a P-1000 micropipette puller (Sutter Instrument, Novato, USA) and coated with Pd/Pt by a sputter coater 108auto (Cressington, Dortmund, Germany). The protonated or deprotonated precursor ions were fragmented by in-source fragmentation via acceleration of the ions and collisions with residual gas molecules (Figure S1). In-source fragmentation is a process comparable to collision-induced dissociation (CID). It relies on the presence of residual air in the differentially pumped source region that acts as buffer gas for ion–molecule collisions. Ions generated by nanoelectrospray ionization are accelerated in the source region by voltage gradients and collide with the residual gas, which induces fragmentation of the ions. Five individually tunable voltages are varied to achieve a suitable voltage gradient: the potential on the source block and the offset and end-cap potentials on the two ring electrode ion guides behind the source block. In general, ions must start from a high potential to undergo sufficient acceleration toward the second ion guide. The instrument does not allow *m*/*z* selection of precursor ions prior to fragmentation, resulting in the activation of all ions that are present after ionization.

The fragment ions are selected by their *m*/*z* in a quadrupole and guided into a hexapole ion trap, where they are thermalized by precooled helium buffer gas (90 K). After the buffer gas is pumped out of the trap, the trapped fragment ions are picked up by a pulsed beam of superfluid helium droplets (0.4 K), which coaxially traverse the trap. The droplets are generated by the expansion of helium (60 bar) through the cryogenic nozzle (19 K) of a pulsed Even-Lavie valve (10 Hz). Each droplet can pick up one ion and transport it to the interaction region, where the doped droplets interact with the pulsed beam (10 Hz macro-pulse repetition rate) of the Fritz Haber Institute free-electron laser (FHI FEL).^29^ If a vibrational transition of the ion is resonant with the photon energy, the IR photons are sequentially absorbed and induce evaporation of the helium shell. After the absorption of multiple photons, the ion is released from the droplet and analyzed by time-of-flight MS. The final IR spectra are constructed by plotting the ion signal on the time-of-flight detector against the photon energy as an indirect measure for IR absorption. IR spectra were measured by scanning the photon energy in steps of 2 cm^–1^. Each spectrum was averaged from two individual scans.

### Ion Mobility-Mass Spectrometry and Collision-Induced Dissociation

Drift-tube ion mobility-mass spectrometry (DT-IM-MS) and tandem mass spectrometry measurements were performed on a modified Synapt G2-S HDMS instrument (Waters Corporation, Manchester, UK) containing a drift tube instead of the commercial traveling wave cell.^30^ The chromane derivatives in methanol (50 μM) were ionized by nanoelectrospray ionization. CID was performed in the trap cell by applying voltages between 10–15 V. Drift times of precursor and fragment ions were acquired in helium at 10 different drift voltages, and CCS were calculated based on the Mason-Schamp equation.^31^ The measurements were repeated on three different days and averaged.

### Computational Methods

A list of candidate structures corresponding to the *m*/*z* of each investigated fragment was generated manually and with the aid of the QCxMS program (version 5.1.3)^32^ using the CID^33,34^ and the dissociative electron attachment (DEA)^35^ modes. QCxMS computes fragmentation in Born–Oppenheimer molecular dynamics employing the semiempirical method GFN2-xTB.^36,37^ For CID simulations, the input parameters were set to “xtb2”, “cid”, “elab 60”, “fullauto”, and for anions “charge −1”. For the DEA mode, the input commands “xtb2”, “dea” were used. For visualization of the results, the entirety of xyz structure output files were analyzed to extract the exact masses and abundance of all generated fragments, both charged and neutral. It was manually confirmed whether the structures with the searched atomic composition indeed correspond to the proposed fragment structure.

The conformational space of each candidate structure was then sampled in CREST (version 2.9)^38^ with GFN2-xTB^36^ and default settings. The lowest-energy conformer was reoptimized in Gaussian 16^39^ at the PBE0+D3/6-311+G(d,p) level of theory^40,41^ using default grid settings. Harmonic frequencies were computed at the same level of theory and were scaled by an empirical factor of 0.965. Harmonic free energies (Δ*F*) were determined at 90 K, which corresponds to the temperature of the conformational ensemble in the ion trap before shock-freezing the ions in the helium droplets. Gibbs energies (Δ*G*) are given at room temperature.

Fragmentation mechanisms were computed in Gaussian 16 by increasing the distance of the bond to be broken in a relaxed potential energy surface (PES) scan with geometry optimization after each step. Based on the confirmed product ion structure, several pathways leading from the precursor to the fragment ion were tested, and only scans leading to a transition state were further considered. After optimization of the structure at the saddle point of the PES as a transition state, the existence of a single imaginary frequency along the reaction coordinate was confirmed by a harmonic frequency analysis. The transition states thus obtained were linked to the corresponding reactants and products by an intrinsic reaction coordinate calculation to generate energetic reaction profiles. The reactants, transition states and products were optimized at the PBE0+D3/6-311+G(d,p) level of theory.

CCS of optimized structures were computed using the software HPCCS,^23^ which is based on the trajectory method (TM).^25^ The CCS were computed at 298.15 K (25 °C) in helium based on density functional theory (DFT)-computed Merz–Singh–Kollman charges.^42^

The spectra prediction tool of CFM-ID 4.0^43^ was accessed via https://cfmid.wishartlab.com. ESI-QToF MS/MS spectra were generated for protonated and deprotonated Trolox and Methyltrolox. The predicted fragment structures did not depend on the simulated collision energy.

## Results and Discussion

### Dehydration and Decarbonylation of Protonated Chromanes

At first, we investigated the fragmentation of protonated Trolox and Methyltrolox using CID. The MS^2^ spectra of the two chromane derivatives show the same fragments, shifted by the mass of a methyl group, which indicates that protonated Trolox and Methyltrolox follow the same fragmentation routes (Figure 1b). The two main fragmentation reactions include the well-studied retro-Diels–Alder reaction (*m*/*z* 165 and 179, respectively),^44,45^ and the net neutral loss of formic acid (CH_2_O_2_). To investigate the latter fragmentation reaction, gas-phase IR spectra of protonated Trolox (*m*/*z* 251) and the resulting fragment (*m*/*z* 205) were recorded using a custom-built instrument described previously.^27,28^

Briefly, the analytes are ionized by nanoelectrospray ionization and fragmented in-source. The fragments of interest are selected by their *m*/*z*, thermalized in an ion trap, and picked up by superfluid helium droplets, which have an intrinsic temperature of 0.4 K. The ion-doped droplets are then irradiated with IR light provided by the Fritz Haber Institute free-electron laser (FHI FEL).^29^ If the photon energy is resonant with a vibrational transition of the ion, the ion absorbs a photon, and the energy is dissipated by evaporation of surrounding helium. The ion relaxes back into its vibrational ground state and can absorb the next photon, until the ion is released from the droplet after multiple photon absorption events. The detection of released ions is thus an indirect measure for IR absorption. The use of superfluid helium droplets increases the spectroscopic resolution compared with classical infrared multiple photon dissociation spectroscopy because ions are held at cryogenic temperatures and the internal ion energy does not increase over the course of photon absorption.

**Figure 2:**
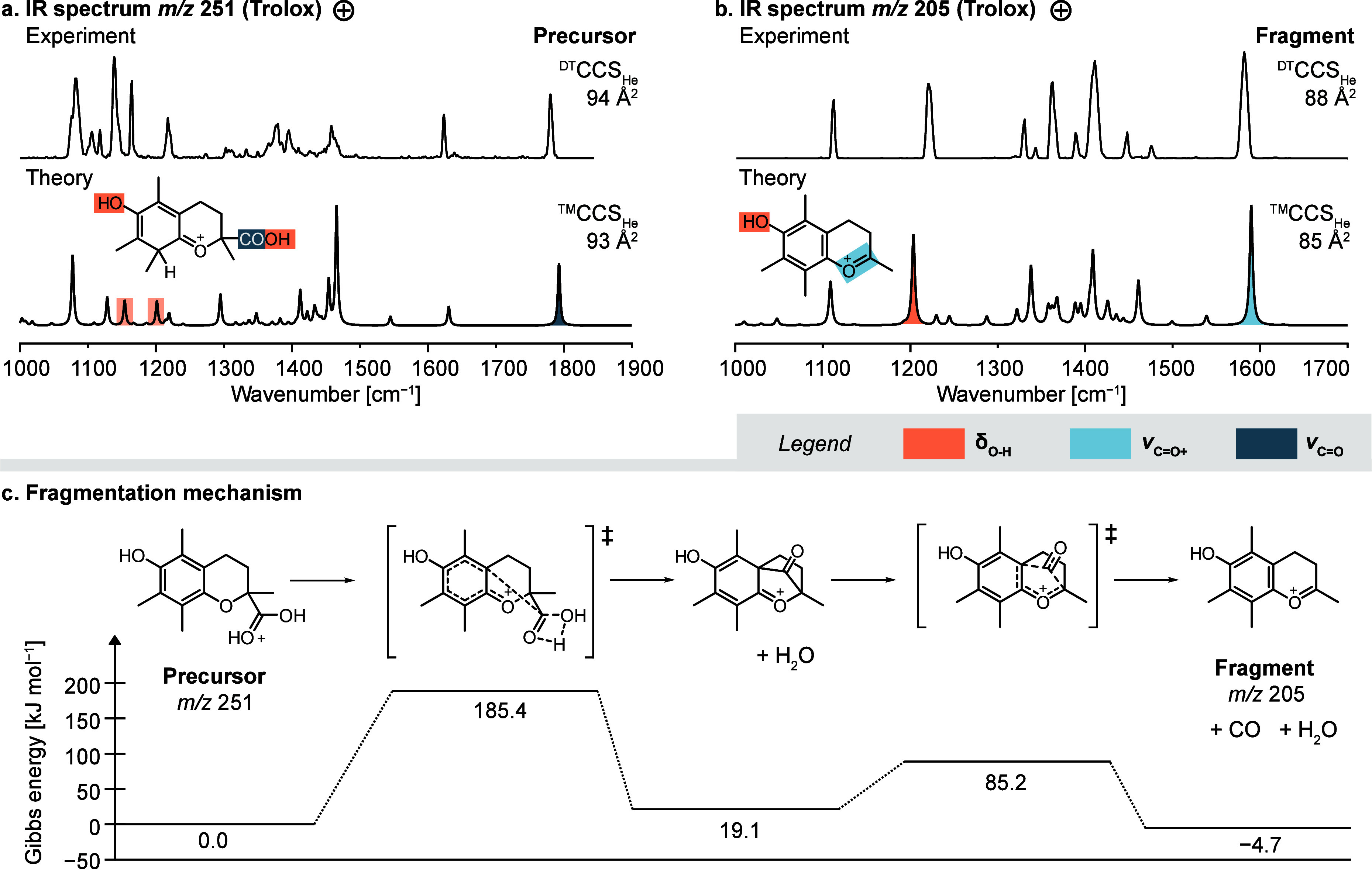
Fragmentation of protonated Trolox. (a) Trolox is protonated on the aromatic ring, as confirmed by matching the experimental and computed IR spectra. (b) The fragment derived from neutral loss of CH_2_O_2_ features an intact chromane scaffold. (c) The underlying fragmentation reaction requires an initial proton transfer to the carboxylic acid (not shown), followed by loss of water and carbon monoxide. IR spectra and energetics were computed at the PBE0+D3/6-311+G(d,p) level of theory. TM = Trajectory method.

The IR spectra of the protonated Trolox precursor ion and the fragment resulting from neutral loss of CH_2_O_2_ (*m*/*z* 205) are shown in Figure 2a and 2b, respectively. To assign the vibrational bands and match structures to the measured spectra, all potential protonation sites on the neutral molecules were computationally identified using the sampling tool CREST,^38^ followed by geometry optimization and harmonic frequency analysis of the protomers by DFT at the PBE0+D3/6-311+G(d,p)^40,41,46^ level of theory. The search resulted in seven protomers for protonated Trolox, which correspond to protonation of the phenol oxygen, ring oxygen, or carboxyl oxygen, or protonation of the benzene ring at four possible positions (Figure S3). Even though protonation of the benzene ring breaks its aromaticity, it is energetically more favorable than protonation of any of the oxygen atoms because the positive charge is stabilized by the phenol or ring oxygen.

The computed IR spectra of the four benzene ring protomers contain the main vibration bands that are experimentally observed for protonated Trolox, including the C=O stretching (ν) vibration of the carboxyl group at 1800 cm^–1^ and O–H bending (δ) vibrations between 1100–1200 cm^–1^. The structure and computed spectrum of the lowest-energy protomer is shown in Figure 2a. Even though the other protomers have similar computed IR spectra and theoretical CCS, their relative free energies are between 11–19 kJ mol^–1^ higher. Assuming that the protomers can interconvert, the contribution of other protomers to the experimental spectrum is negligible based on the expected Boltzmann distribution at the ion trap temperature (90 K).

The fragment at *m*/*z* 205 corresponding to the loss of CH_2_O_2_ was investigated using the same approach. Its IR spectrum shows the most intense band at 1582 cm^–1^, which indicates a C=O^+^ stretching vibration (Figure 2b). Accordingly, the structure which provides the best spectral match features an intact chromane scaffold with the positive charge being stabilized by the ring oxygen. The phenol oxygen is protonated, which is reflected in the O–H bending vibration at 1200 cm^–1^. Hence, the phenol oxygen does not participate in the fragmentation and charge transfer, while the ring oxygen stabilizes the positive charge.

The net loss of CH_2_O_2_ could proceed via an initial loss of water followed by decarbonylation, as previously suggested for small organic carboxylic acids.^47^ To confirm this hypothesis, transition states and activation barriers were computed by DFT. For the initial loss of water, the proton must be transferred from the aromatic ring to the carboxyl group. The computed activation barrier for the proton transfer reaction is 58 kJ mol^–1^ (Δ*G*, Figure S7). Once the carboxyl group is protonated, it can either directly eliminate water (Figure 2c) or first form a geminal diol intermediate (Figure S8). Both pathways result in a bicyclic ketone, which yields the spectroscopically observed product ion (*m*/*z* 205) by decarbonylation. The thermal decarbonylation of unsaturated bicyclic ketones is a well-known organic reaction,^48^ which has been used, for example, to obtain isotopically labeled CO in a reaction sequence that is strikingly similar to the fragmentation reaction observed here in the gas phase.^49^

The proposed mechanism was further supported by molecular dynamics simulations of the CID process using the program QCxMS.^32−34^ Only the protonated carboxylic acid but not the most stable protomer yields the observed fragment ion (Figures S9–S10). Hence the dissociation of water requires an initial proton transfer from the aromatic ring to the carboxylic acid. This example demonstrates that the dynamics of mobile protons can generate higher-energy protomers, which provide favorable geometrical conditions for fragmentation reactions. Because the phenol group is not involved in the reaction, the results are expected to be transferable to Methyltrolox.

### Decarboxylation and Ring Opening of Deprotonated Chromanes

Contrary to the protonated precursors, the fragmentation reactions of deprotonated Trolox and Methyltrolox significantly differ from each other (Figure 1b). Deprotonated Trolox yields one major fragment (*m*/*z* 205) resulting from decarboxylation of the precursor ion and the retro-Diels–Alder product at *m*/*z* 163, whereas deprotonated Methyltrolox yields a range of radical fragments by sequential loss of methyl radicals and decarboxylation, which is unusual for closed-shell precursor ions.

**Figure 3:**
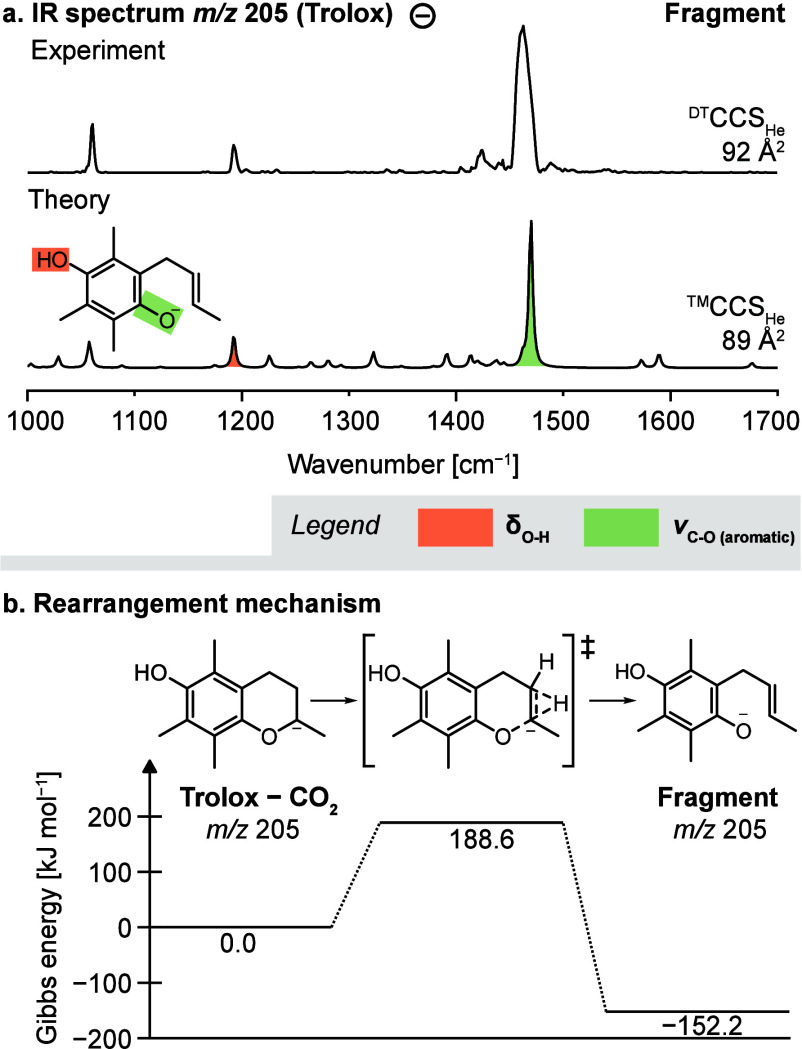
Fragmentation of deprotonated Trolox. (a) The IR spectrum of the fragment derived from decarboxylation matches the computed spectrum of a *para*-hydroxyl phenolate. (b) The computed reaction mechanism suggests that the initial decarboxylation is followed by a proton transfer, which induces ring opening. IR spectra and energetics were computed at the PBE0+D3/6-311+G(d,p) level of theory.

The fragment at *m*/*z* 205 resulting from neutral loss of CO_2_ from deprotonated Trolox has the same mass as the protonated fragment resulting from loss of CH_2_O_2_, but the CCS of the anion is 4 Å^2^ larger than that of the cation. The IR spectrum of the anion features an intense band below 1500 cm^–1^, which corresponds to the C–O stretching vibration of a phenolate that involves C=C stretching vibrations of the aromatic ring (Figure 3a). A second band that is indicative of an O–H bending vibration is located below 1200 cm^–1^. Accordingly, the best match is obtained for a *para*-hydroxyl phenolate resulting from opening of the dihydropyrane ring. The structure correlates well with the increased CCS, as the alkyl chain is more extended than the closed ring of the protonated fragment structure.

An energetic evaluation of deprotonated Trolox precursor ions suggests that the negative charge is initially localized at the carboxyl rather than the phenol group (Figure S3). Hence, the negative charge is expected to be transferred from the carboxylate to the ring oxygen, which is accompanied by ring opening. The CID simulation of deprotonated Trolox in QCxMS yields abundant fragment ions with an intact chromane scaffold resulting from decarboxylation (Figure S14). After proton transfer from either of the two neighboring carbon atoms, the dihydropyrane ring opens to stabilize the negative charge on the ring oxygen (Figure 3b). The emerging C=C bond in the aliphatic chain can be formed at two different positions, which cannot be distinguished based on their computed IR spectra (Figure S12).

The fragment at *m*/*z* 204 in the MS^2^ spectrum of deprotonated Methyltrolox has an analogous structure to decarboxylated Trolox, with the difference that the phenol oxygen bears an unpaired electron (Figure S16). The electron is stabilized between the two oxygens and the aromatic ring like in vitamin E phenoxyl radicals, which stabilize unpaired electrons and break radical chain reactions in lipid membranes.^50^ The fragmentation can proceed via the same reaction mechanism with comparable computed activation barriers as deprotonated Trolox (Figure S18).

### Wittig Rearrangement of Deprotonated Methyltrolox

**Figure 4:**
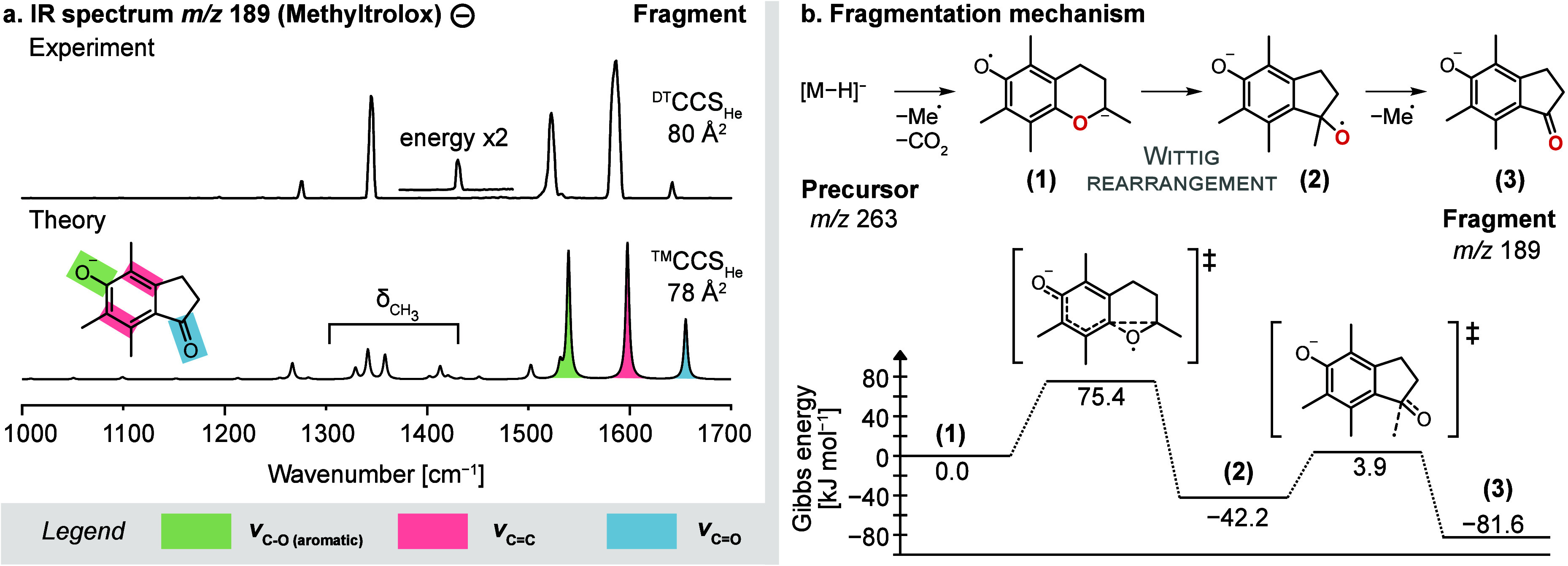
Fragmentation of deprotonated Methyltrolox. (a) The IR spectrum of the even-electron fragment at *m*/*z* 189 matches with the computed spectrum of the depicted indanone derivative. The band at 1420 cm^–1^ is only visible at higher laser pulse energy and is shown as an inset. (b) The proposed fragmentation reaction involves a [1,2]-Wittig rearrangement, followed by the loss of a methyl radical. IR spectra and energetics were computed at the PBE0+D3/6-311+G(d,p) level of theory.

The fragmentation of deprotonated Methyltrolox is more complex than that observed for deprotonated Trolox. Negative ionization of Methyltrolox yields a carboxylate, which can eliminate a methyl radical from the phenol oxygen (Δ*G* = 125 kJ mol^–1^) to yield a radical anion at *m*/*z* 248 (Figure S15). Apart from the unpaired electron, the structure is analogous to deprotonated Trolox. However, the unpaired electron induces fragmentation reactions that are not observed for even-electron Trolox anions, resulting in an additional fragment at *m*/*z* 189 that has no equivalent in the fragment spectrum of Trolox. To theoretically simulate the radical fragmentation pathways observed for deprotonated Methyltrolox, QCxMS was employed in the dissociative electron attachment (DEA) mode.^35^ DEA involves ionization of a neutral molecule by the attachment of a low-energy electron *in silico* to simulate dissociation of radical anions. For the radical anion resulting from abstraction of a methyl radical from deprotonated Methyltrolox (*m*/*z* 248), the simulation exclusively yielded a closed-ring radical anion with *m*/*z* 204 via decarboxylation (structure (1) in Figure 4b). The computed energetic barrier for the decarboxylation reaction is in the range of 80 kJ mol^–1^ (Δ*G*). The closed-ring radical anion is the putative precursor for the ring opening reaction leading to the spectroscopically observed and previously discussed fragment at *m*/*z* 204 (Figure S18).

Assuming that the radical anion (1) is also the precursor of the other fragment observed in the MS^2^ spectrum of deprotonated Methyltrolox (*m*/*z* 189), we took structure (1) as input for a second DEA simulation. The simulation yielded abundant loss of hydrogen radicals, which is not observed in the experimental mass spectra and is probably an artifact of the simulation. However, the simulation also yielded the sought product ion at *m*/*z* 189 (Figure S21). Its structure features an indanone scaffold, and the corresponding computed IR spectrum and CCS show good agreement with the experiment (Figure 4a). Especially the C=O stretching vibration at 1650 cm^–1^ is diagnostic for the formation of a ketone. There are two additional bands at 1600 cm^–1^ and between 1500–1550 cm^–1^, which can be assigned to the C=C stretching vibrations of the benzene ring and the C–O stretching vibration of the phenolate.

Several low-intensity bands predicted by theory are not visible or only visible at increased laser pulse energies in the experimental spectrum. Because the experimental intensities scale nonlinearly with the photon flux in multiple photon absorption experiments, relative intensities deviate from linear absorption spectra.^51^ In practice, it is necessary to find a compromise between saturation of high-intensity bands and visibility of low-intensity bands. Overall, we obtain a convincing match that contains vibrations of all characteristic functional groups of the proposed fragment structure.

Despite the good match between experiment and theory, the underlying rearrangement reaction is intriguing as it involves the dissociation of an aryl ether bond. A very similar reaction has been reported in the condensed phase, namely the ring contraction of 6*H*-benzo[*c*]chromene to 9-fluorenol.^52^ This isomerization reaction is a [1,2]-Wittig rearrangement, which yields secondary or tertiary alcohols from α-deprotonated ethers.^53^ The migration of neutral and electron-poor aryl ethers in solution is thought to proceed via a concerted anionic addition/elimination mechanism.^54^ In the gas phase, theoretical studies on aryl ethers suggest a two-step addition/elimination mechanism with a very low activation barrier for the second transition state (ca. 2 kJ mol^–1^).^55^

Assuming an addition/elimination mechanism, the energetic profile of the fragmentation reaction of deprotonated Methyltrolox was computed (Figure 4b). The results suggest that the isomerization reaction proceeds via a concerted mechanism with a low activation barrier (Δ*G* = 75 kJ mol^–1^). Gas-phase [1,2]-Wittig rearrangements of aryl ethers have been previously postulated based on fragmentation patterns.^56,57^ Even though the intermediates (1) and (2) were not experimentally detected, the major structural changes from the precursor to the confirmed fragment structure (3) and the favorable energetics provide strong evidence that a [1,2]-Wittig rearrangement occurs the gas phase.

### Integration of Fragmentation Pathways

**Figure 5:**
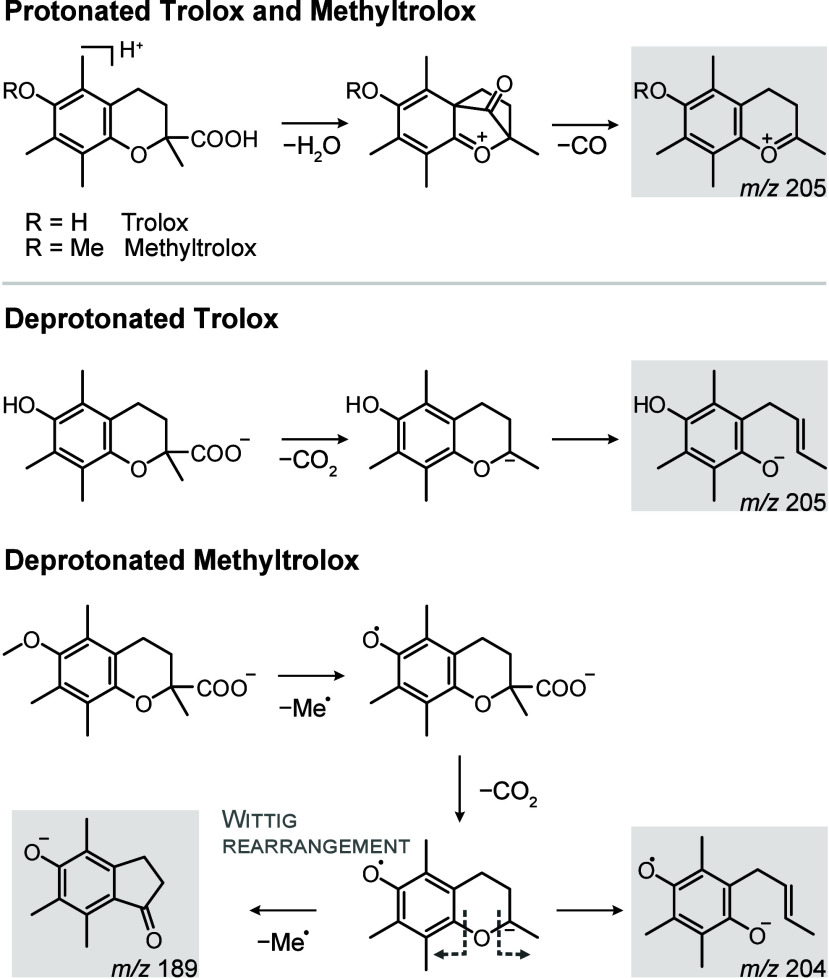
Overview of the fragmentation reactions studied in this work. The fragment structures highlighted in gray have been confirmed by IR spectroscopy.

An overview of the investigated reactions and fragment structures is provided in Figure 5. Protonated Trolox and Methyltrolox yield a closed-ring fragment resulting from dehydration and decarboxylation of the protonated precursor, which requires initial proton transfer to the carboxyl group. Deprotonated Trolox readily decarboxylates, and the resulting fragment undergoes a proton rearrangement reaction accompanied by ring opening. The fragmentation of deprotonated Methyltrolox deviates from deprotonated Trolox due to its inability to stabilize a negative charge on the methoxy group. Elimination of the methyl radical results in a structure that is analogous to deprotonated Trolox, with the difference that the phenol oxygen now carries an unpaired electron. After decarboxylation, the ion can undergo two competing reactions: ring opening or a Wittig rearrangement, forming the fragments at *m*/*z* 204 and *m*/*z* 189, respectively. For deprotonated Trolox, no Wittig rearrangement is observed because the phenol group cannot stabilize the negative charge.

We tested our results against CFM-ID 4.0 (Competitive Fragment Modeling Identification), which generates MS/MS spectra for the Human Metabolome Database.^43^ None of the fragment structures identified by IR spectroscopy are correctly predicted by the machine learning-based algorithm (Table S5). Expanding the training set with more experimentally confirmed fragment ion structures from different molecule classes will likely increase the accuracy of structure predictions by machine learning- and rule-based fragmentation algorithms.

## Conclusions

In conclusion, we investigated the impact of protonation and methylation on the fragmentation behavior of two chromane derivatives, Trolox and Methyltrolox. Using a combination of gas-phase infrared spectroscopy, ion mobility-mass spectrometry, and computational chemistry, we identified fragment ion structures and proposed dissociation reaction mechanisms that link the precursor to the product ion. We observed rearrangement reactions involving mobile protons, and radical fragmentation reactions resulting in a putative [1,2]-Wittig rearrangement in the gas phase. Overall, our results reveal close structure–reactivity relationships and highlight that fragmentation is driven by the availability of protons and charge-stabilizing functional groups.

It should be noted that the use of in-source fragmentation in our experimental setup presents several shortcomings in identifying product ion structures. For example, residual solvent molecules in the ion source could act as intermolecular proton shuttle agents,^58^ which are not available in collision cells. Furthermore, ions are not *m*/*z*-selected prior to fragmentation. Therefore, we are not able to analyze mixtures because generated fragments cannot be assigned back to their precursors. Coupling with liquid chromatography for mixture separation is also not feasible due to incompatible IR spectroscopy and LC time scales. However, similar instruments with *m*/*z* selection and a separate collision cell and/or significantly reduced acquisition times are capable of measuring product ions of defined precursors even in complex mixtures.^14,59−61^

Overall, this and other studies^4,14−20^ contribute to the understanding of gas-phase dissociation reactions. A fundamental understanding of fragmentation mechanisms brought by IR spectroscopy and computational chemistry can help to predict fragmentation of other compounds based on their structural features using rule-based approaches. Furthermore, experimentally confirmed fragment ion structures can be fed into the training data used to train machine learning-based fragmentation tools to improve the accuracy of their structural predictions.

## Data Availability

The data underlying this study are available in the published article and its Supporting Information.

## Supporting Information Available

Mass spectra, computed structures, additional IR spectra, and collision cross sections (PDF)
