# Supplementary material for: Studying the Intrinsic Reactivity of Chromanes by Gas-Phase Infrared Spectroscopy
Source: J Am Soc Mass Spectrom. 2024 Jul 1;35(8):1950–8. doi: 10.1021/jasms.4c00216 (PMC11311547; doi:10.1021/jasms.4c00216)
Supplement: Supplementary file 1 — js4c00216_si_001.pdf [file js4c00216_si_001.pdf]

Studying the Intrinsic Reactivity of Chromanes by Gas-Phase Infrared Spectroscopy

Supplementary Information

Carla Kirschbaum,[a,b] Kim Greis,[a,b] América Y. Torres-Boy,[b] Jerome Riedel,[a,b] Sandy Gewinner,[b] Wieland Schöllkopf,[b] Gerard Meijer,[b] Gert von Helden,[b] and Kevin Pagel*[a,b]

a Institut für Chemie und Biochemie, Freie Universität Berlin, 14195 Berlin, Germany

b Fritz-Haber-Institut der Max-Planck-Gesellschaft, 14195 Berlin, Germany

Correspondence to: pagel@fhi-berlin.mpg.de

Table of Contents

In-Source Fragmentation **3**
Tandem Mass Spectra **4**
Precursor Ion Structures **5**
Positive Ion Mode: Dehydration and Decarbonylation of Trolox **7**
Negative Ion Mode: Decarboxylation of Trolox **15**
Negative Ion Mode: Radical Fragmentation of Methyltrolox **20**
Collision Cross Sections **28**
Comparison with Structure Prediction **28**
Coordinates of Computed Structures **29**
Trolox *m/z* 249 (−) 29
Trolox *m/z* 251 (+) 29
Methyltrolox m/z 263 (−) 32
Methyltrolox m/z 265 (+) 32
Trolox *m/z* 205 (+) 35
Trolox *m/z* 251 → 205 (+) 37
Trolox *m/z* 205 (−) 44
Trolox *m/z* 249 → 205 (−) 47
Methyltrolox *m/z* 248 (−) 50
Methyltrolox *m/z* 204 (−) 52
Methyltrolox *m/z* 263 → 204 (−) 55
Methyltrolox *m/z* 189 (−) 57
Methyltrolox *m/z* 204 → 189 (−) 59

In-Source Fragmentation

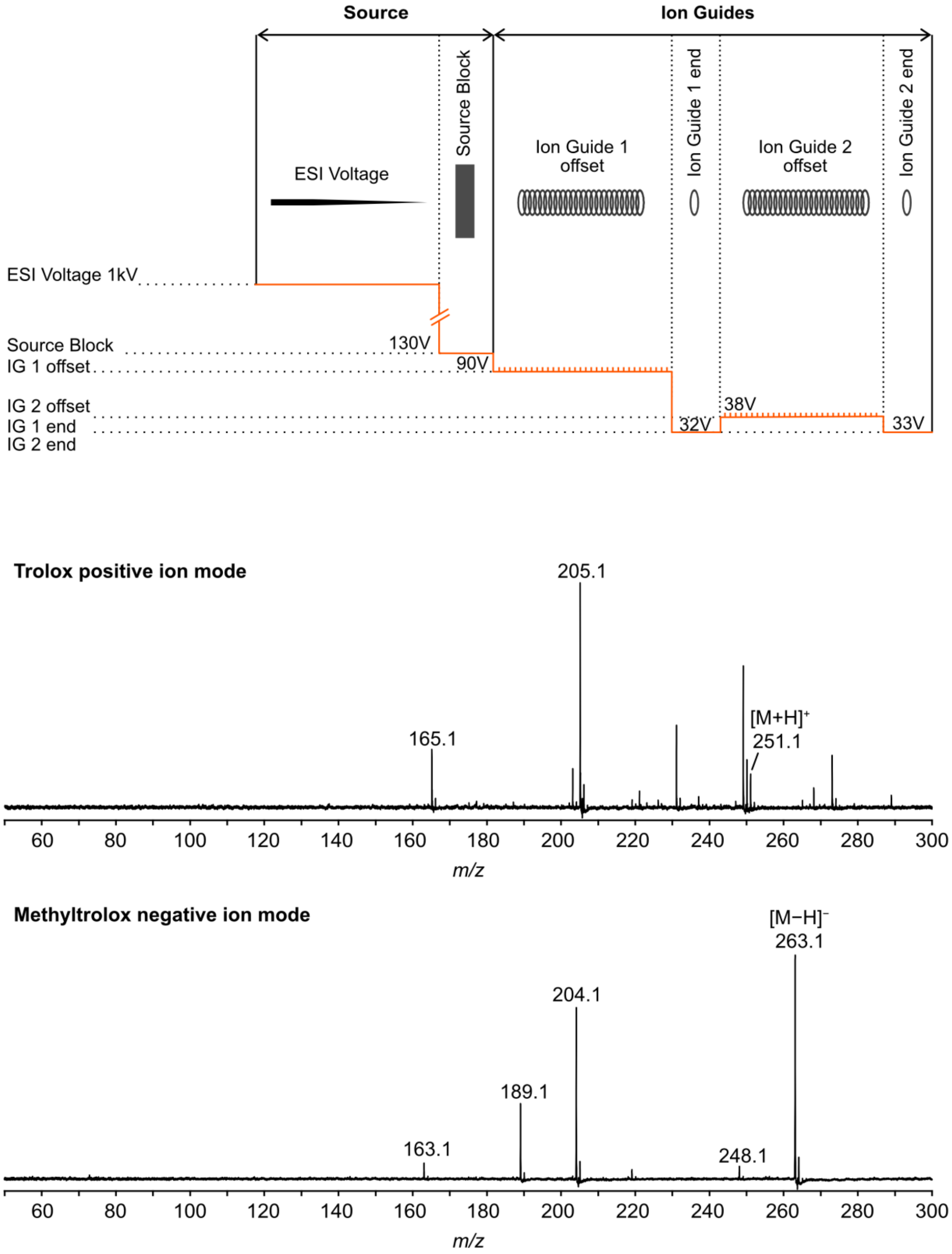

Figure S1. Voltage scheme of the source region to induce in-source fragmentation. Precursor ions are generated by nano-electrospray ionization and accelerated from the source block towards the second ion guide (IG). Empirically determined values employed for the fragmentation of Trolox in the positive ion mode are shown in the scheme (drawing is not to scale). Source fragmentation spectra of Trolox and Methyltrolox are shown below with assigned ions highlighted.

Tandem Mass Spectra

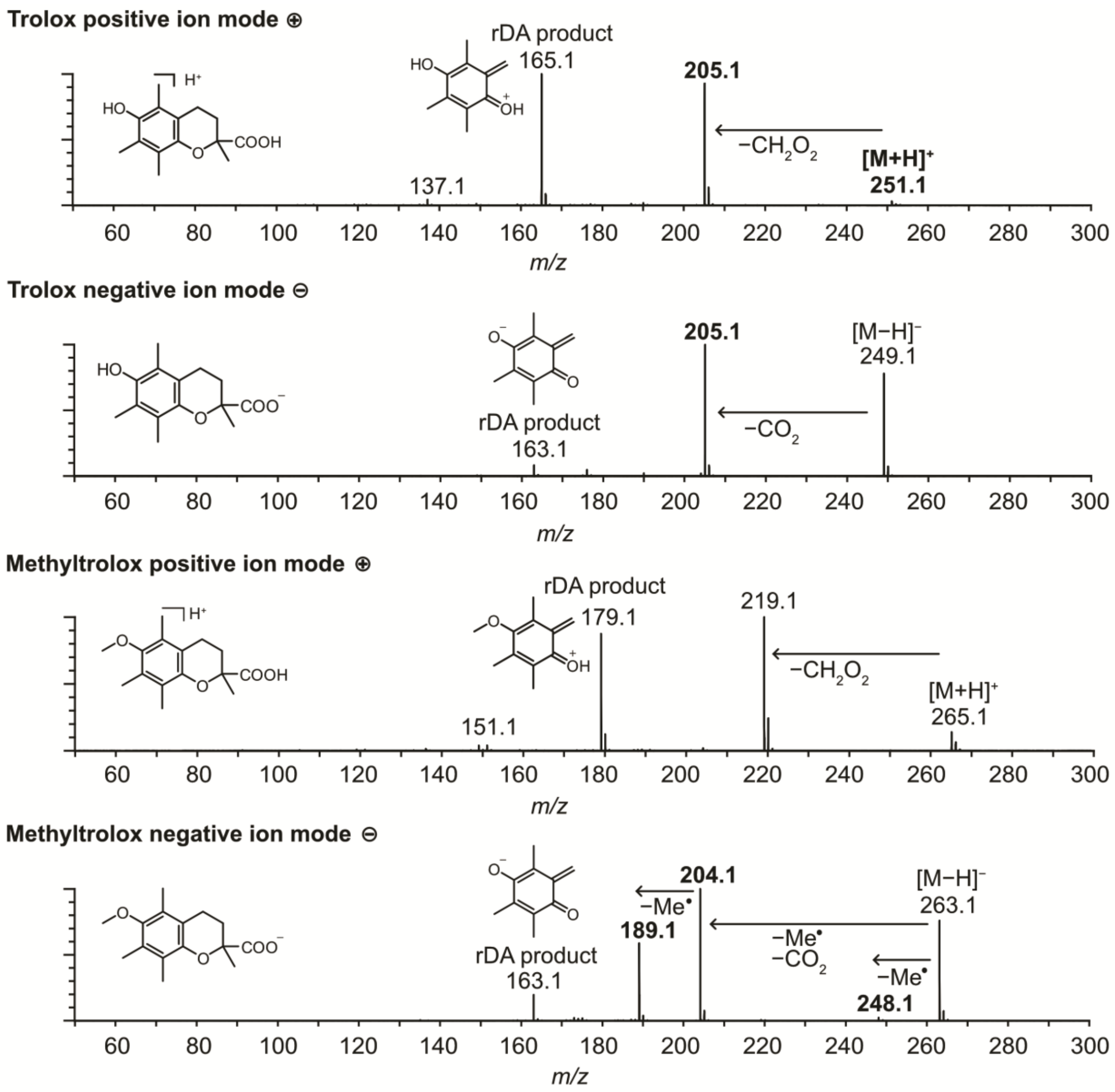

Figure S2. Tandem mass spectra of protonated and deprotonated Trolox and Methyltrolox recorded on a Synapt G2-S HDMS instrument. The ions highlighted in bold have been investigated by IR spectroscopy in this work. Retro-Diels-Alder (rDA) products are shown.

Precursor Ion Structures

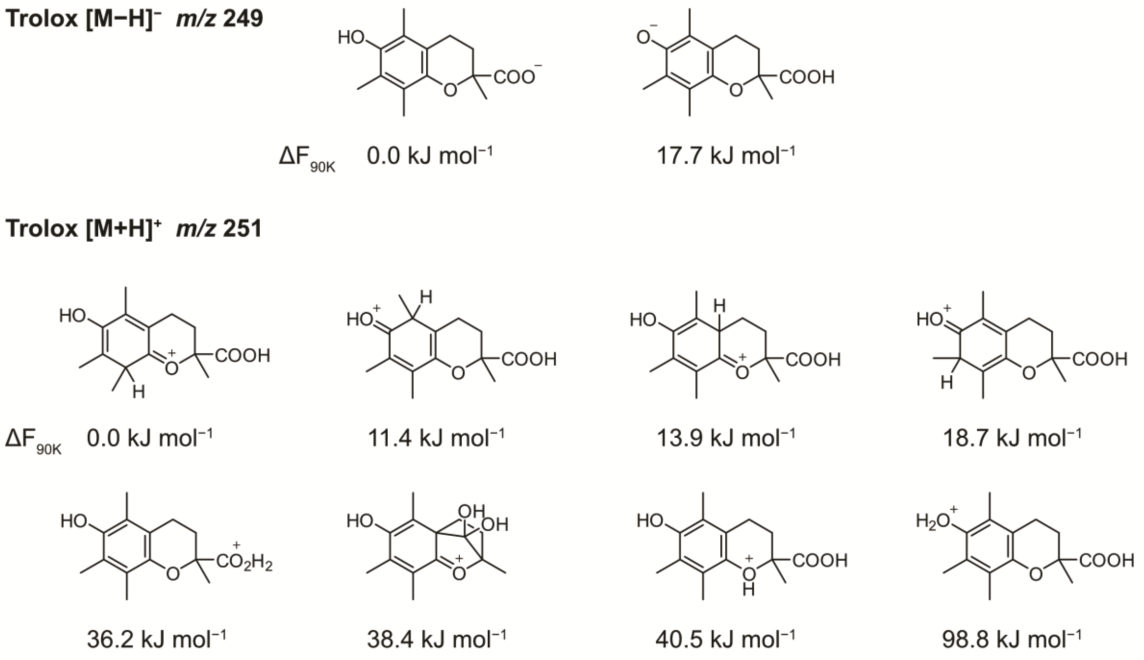

Figure S3. Structures and relative free energies of deprotonated and protonated Trolox precursor ions. Due to its acidity, the carboxyl group is preferentially deprotonated rather than the phenolic hydroxyl group. Protonation of the aromatic ring is energetically more favorable than protonation of the oxygen atoms. XYZ coordinates of all structures are listed on page 29ff.

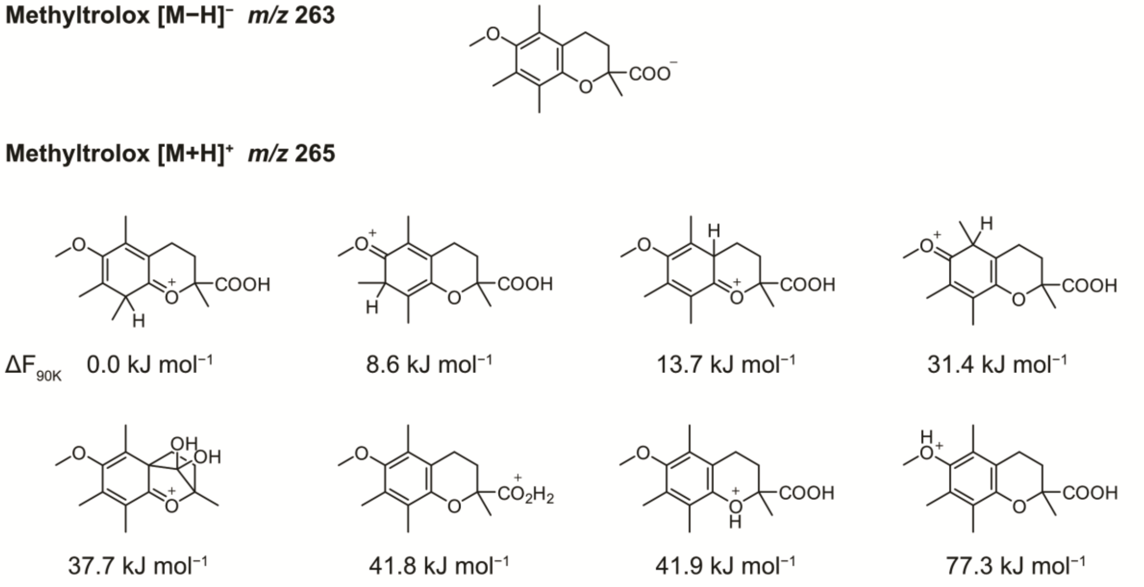

Figure S4. Structures and relative free energies of deprotonated and protonated Methyltrolox precursor ions. The molecule features only one acidic proton at the carboxyl group, which is abstracted upon ionization in negative ion mode. In positive ion mode, protonation of the aromatic ring is energetically more favorable than protonation of the oxygen atoms. XYZ coordinates of all structures are listed on page 32ff.

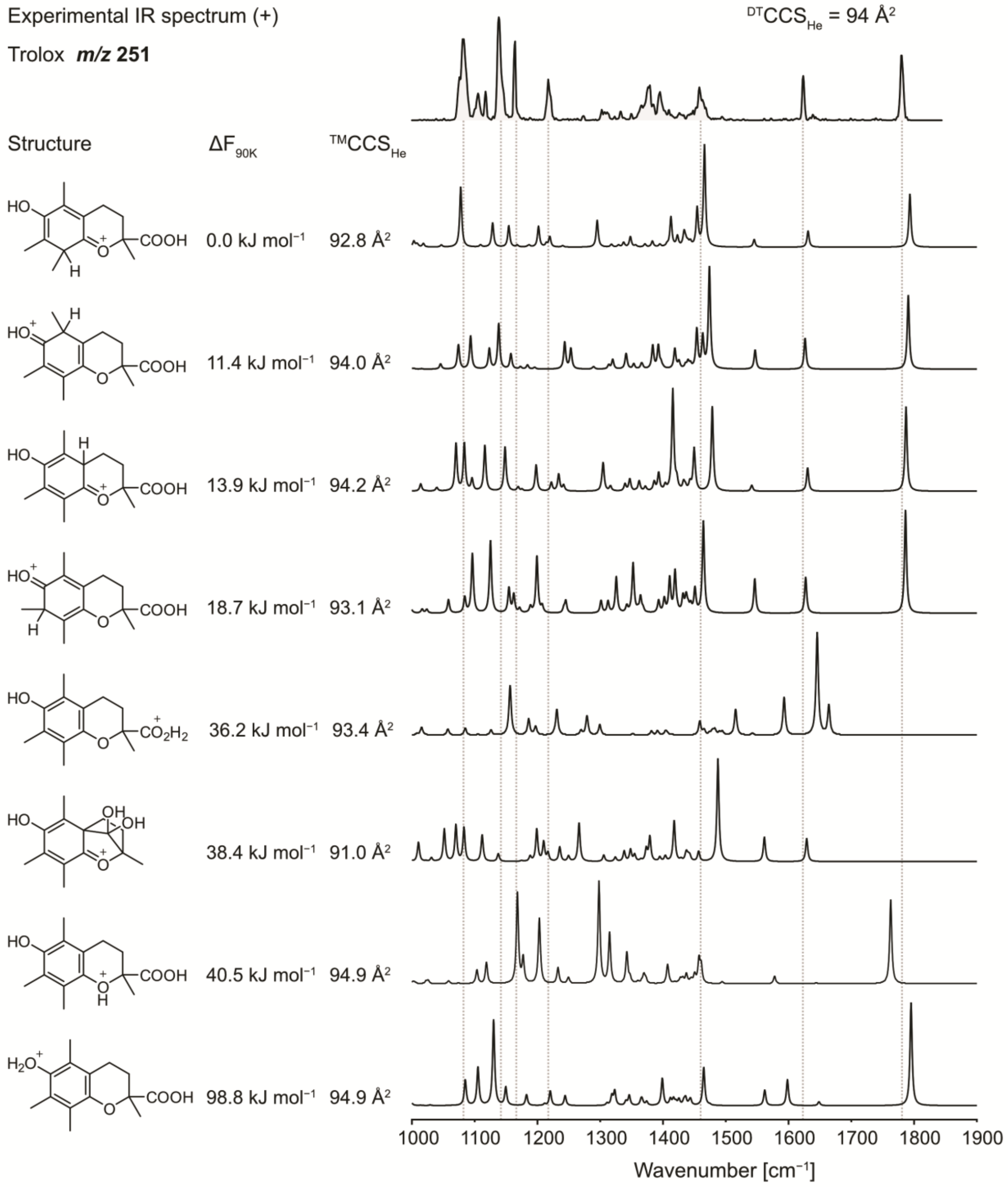

Figure S5. Experimental IR spectrum of protonated Trolox (*m/z* 251) and computed IR spectra of candidate structures. The absorption bands around 1800 cm^{-1} and 1600 cm^{-1} are diagnostic for protonation of the aromatic ring. The computed structure shown on top yields the best match with the experiment. Spectra were computed at the PBE0+D3/6-311+G(d,p) level of theory and scaled by 0.965. XYZ coordinates of all structures are listed on page 29ff.

Positive Ion Mode: Dehydration and Decarbonylation of Trolox

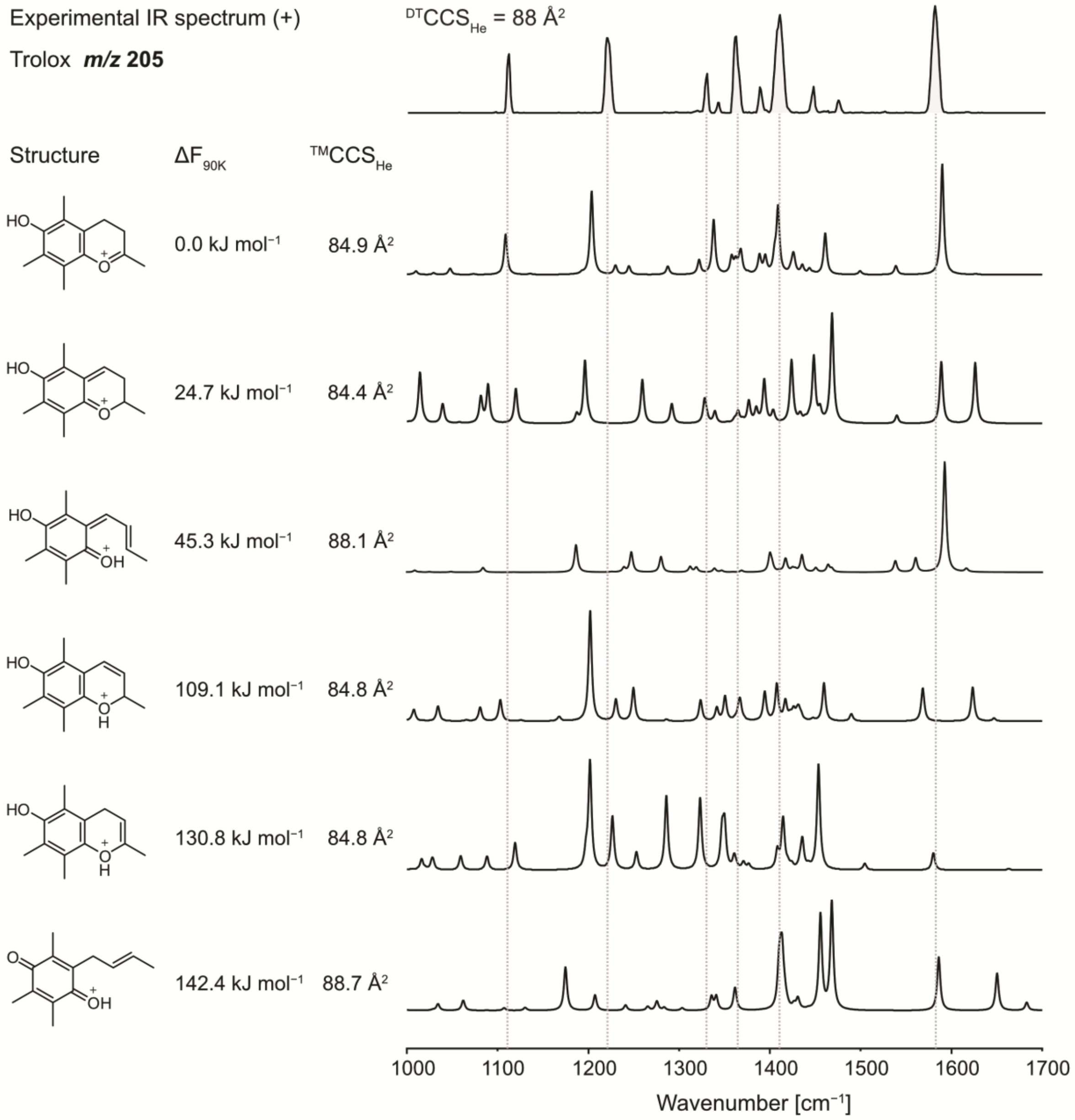

Figure S6. Experimental IR spectrum of the fragment at *m/z* 205 generated from protonated Trolox precursor ions and computed IR spectra of candidate structures. The band at 1200 cm^{-1} indicates the presence of a hydroxyl group, whereas the band at 1600 cm^{-1} corresponds to a $C=O^+$ stretching vibration. The best spectral match is shown on top. Spectra were computed at the PBE0+D3/6-311+G(d,p) level of theory and scaled by 0.965. XYZ coordinates of all structures are listed on page 35ff.

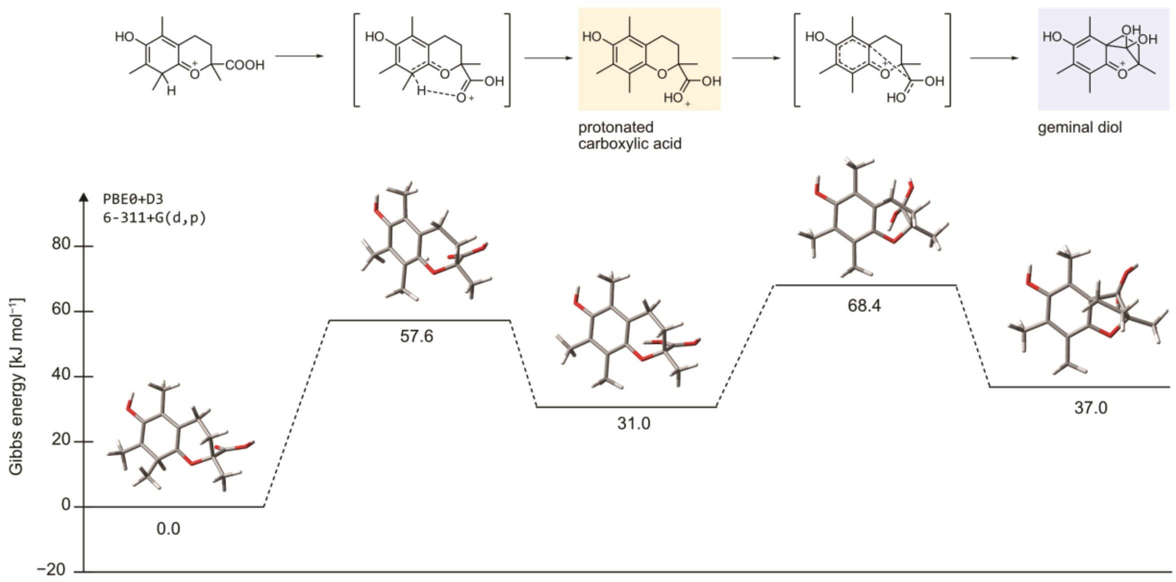

Figure S7. Computed activation barriers for the intramolecular proton transfer in the protonated Trolox precursor. The proton is transferred from the aromatic ring to the carboxyl group. The resulting protonated carboxylic acid can either directly undergo neutral water loss (*cf.* Figure S8a) or first cyclize to yield a geminal diol, which then loses water (*cf.* Figure S8b). The structures were optimized at the PBE0+D3/6-311+G(d,p) level of theory. XYZ coordinates of all structures are listed on page 37ff.

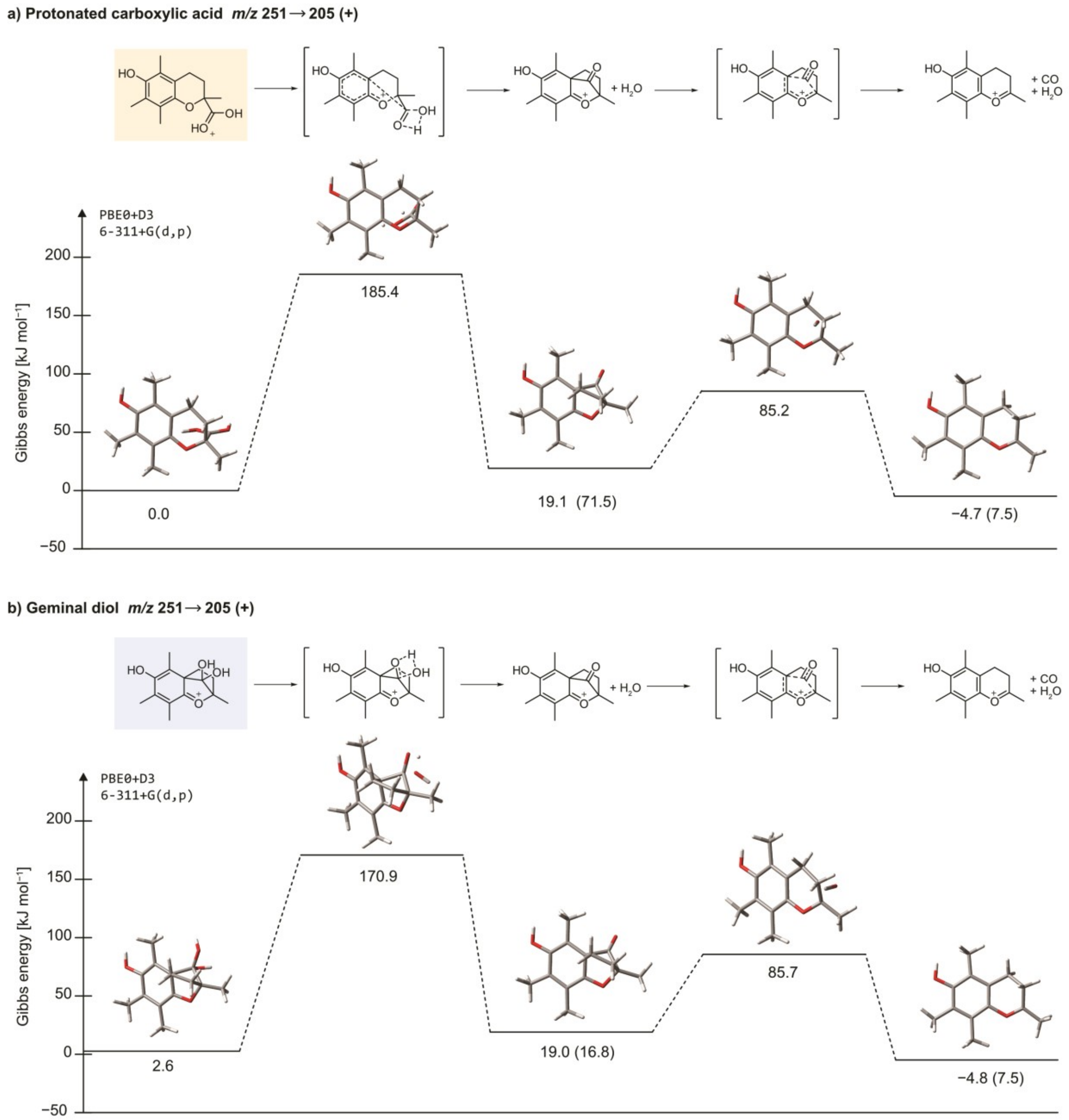

Figure S8. Computed reaction pathway for the neutral loss of water and carbon monoxide from the protonated Trolox precursors from Figure S7. The reaction can proceed via a protonated carboxylic acid (a) or bicyclic geminal diol (b). After an initial proton transfer between the two oxygen atoms of the carboxylic acid or diol, respectively, a water molecule is eliminated. The following reaction steps are identical for both precursors. Decarbonylation of the bicyclic ketone yields the spectroscopically observed product ion at *m/z* 205. The structures were optimized at the PBE0+D3/6-311+G(d,p) level of theory. The Gibbs energy of the associated products, *i.e.,* the cation and the neutral H_2O or CO molecule, before their separation is indicated in brackets. The relative energies are scaled to the protonated carboxylic acid structure in the energy diagram (a), and the energy difference between the protonated carboxylic acid and the diol is not exactly the same as in Figure S7 because the hydroxyl groups are slightly rotated in the diol. XYZ coordinates of all structures are listed on page 39ff.

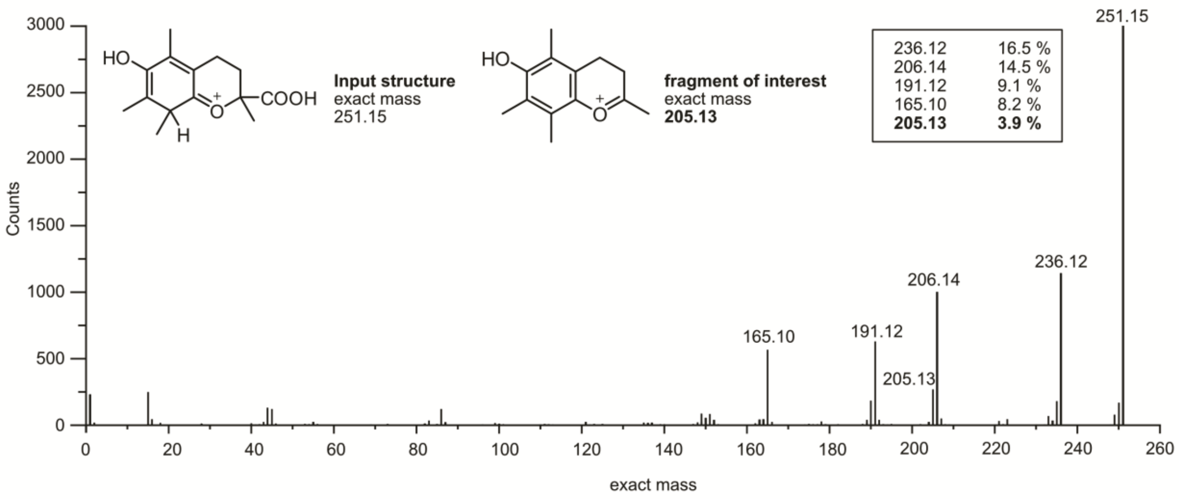

Figure S9. Simulation of collision-induced dissociation of the depicted protonated Trolox precursor in QCxMS. The exact masses of all generated structures were extracted and plotted against their abundance, *i.e.,* the absolute number of xyz output files corresponding to the respective exact mass. The relative abundances of the most abundant fragments are shown and refer to the sum of all generated fragments (excluding the precursor). The fragment of interest (exact mass = 205.13) is formed but the obtained structures do not correspond to the searched structure.

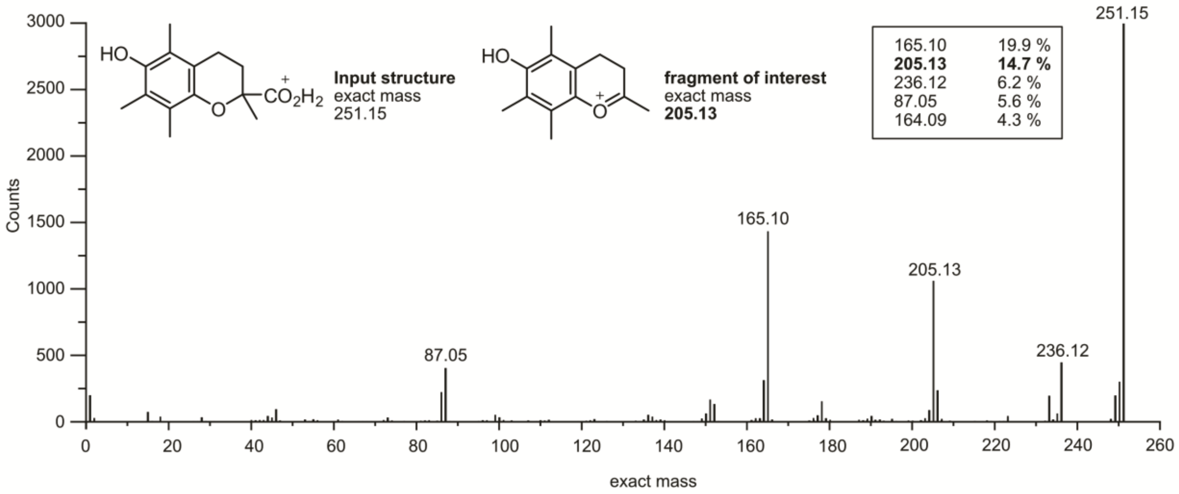

Figure S10. Simulation of collision-induced dissociation of the depicted Trolox precursor protonated at the carboxylic acid in QCxMS. The exact masses of all generated structures were extracted and plotted against their abundance, *i.e.,* the absolute number of xyz output files corresponding to the respective exact mass. The relative abundances of the most abundant fragments are shown and refer to the sum of all generated fragments (excluding the precursor). The fragment of interest (exact mass = 205.13) is the second most abundant fragment after the retro-Diels-Alder product (exact mass = 165.10) and corresponds to the searched structure.

Table S1. Overview of fragments generated in the QCxMS simulation of the protonated Trolox precursor (Figure S9). The exact masses, sum formulas, absolute number of xyz files per fragment and the relative abundance are given.

Exact mass	Sum formula	number	%
1.01	H	233	3.4
2.02	H2	21	0.3
15.02	CH3	250	3.6
16.03	CH4	45	0.7
17.01	HO	4	0.1
18.02	H2O	18	0.3
26.02	C2H2	2	0.0
28.00	CO	14	0.2
28.03	C2H4	10	0.1
29.01	CHO	2	0.0
29.04	C2H5	2	0.0
31.02	CH3O	1	0.0
39.02	C3H3	1	0.0
40.03	C3H4	15	0.2
41.01	C2HO	1	0.0
41.04	C3H5	7	0.1
42.02	C2H2O	8	0.1
42.05	C3H6	5	0.1
43.02	C2H3O	25	0.4
43.06	C3H7	4	0.1
44.00	CO2	133	1.9
44.03	C2H4O	1	0.0
45.01	CHO2	123	1.8
45.04	C2H5O	2	0.0
46.01	CH2O2	14	0.2
47.02	CH3O2	3	0.0
52.03	C4H4	1	0.0
53.04	C4H5	10	0.1
54.02	C3H2O	8	0.1
54.05	C4H6	9	0.1
55.02	C3H3O	8	0.1
55.06	C4H7	25	0.4
56.03	C3H4O	11	0.2
58.05	C3H6O	4	0.1
61.01	CHO3	1	0.0
61.04	C2H5O2	1	0.0
67.02	C4H3O	4	0.1
67.06	C5H7	2	0.0
68.03	C4H4O	4	0.1
69.01	C3HO2	1	0.0
69.04	C4H5O	7	0.1
70.05	C4H6O	2	0.0
71.02	C3H3O2	1	0.0
71.05	C4H7O	1	0.0
71.06	C4H7O	2	0.0
72.03	C3H4O2	2	0.0
73.04	C3H5O2	10	0.1
74.01	C2H2O3	1	0.0
74.05	C3H6O2	1	0.0
81.07	C6H9	4	0.1
82.05	C5H6O	13	0.2
82.08	C6H10	1	0.0
83.05	C5H7O	35	0.5
85.04	C4H5O2	11	0.2
86.05	C4H6O2	122	1.8
87.05	C4H7O2	23	0.3
88.03	C3H4O3	4	0.1
89.04	C3H5O3	1	0.0
91.06	C7H7	1	0.0
94.05	C6H6O	4	0.1
96.03	C5H4O2	1	0.0
96.06	C6H8O	6	0.1
97.04	C5H5O2	2	0.0
97.07	C6H9O	4	0.1
98.05	C5H6O2	3	0.0
99.05	C5H7O2	17	0.2
100.06	C5H8O2	12	0.2
101.07	C5H9O2	8	0.1
102.05	C4H6O3	1	0.0
106.05	C7H6O	3	0.0
109.04	C6H5O2	2	0.0
109.07	C7H9O	4	0.1
110.05	C6H6O2	6	0.1
110.08	C7H10O	6	0.1
111.05	C6H7O2	3	0.0
111.09	C7H11O	11	0.2
112.06	C6H8O2	8	0.1
113.04	C5H5O3	6	0.1
113.07	C6H9O2	2	0.0
114.04	C5H6O3	2	0.0
121.07	C8H9O	26	0.4
121.10	C9H13	6	0.1
122.05	C7H6O2	1	0.0
122.08	C8H10O	6	0.1
123.09	C8H11O	11	0.2
125.07	C7H9O2	10	0.1
128.06	C6H8O3	1	0.0

133.07	C9H9O	4	0.1
134.08	C9H10O	5	0.1
135.09	C9H11O	20	0.3
136.06	C8H8O2	7	0.1
136.09	C9H12O	1	0.0
136.10	C9H12O	18	0.3
137.07	C8H9O2	20	0.3
137.10	C9H13O	18	0.3
138.08	C8H10O2	3	0.0
138.11	C9H14O	1	0.0
139.09	C8H11O2	9	0.1
140.06	C7H8O3	2	0.0
140.09	C8H12O2	8	0.1
146.08	C10H10O	2	0.0
147.09	C10H11O	10	0.1
148.06	C9H8O2	4	0.1
148.10	C10H12O	22	0.3
149.07	C9H9O2	88	1.3
149.10	C10H13O	26	0.4
150.08	C9H10O2	56	0.8
151.05	C8H7O3	2	0.0
151.09	C9H11O2	85	1.2
152.09	C9H12O2	40	0.6
153.10	C9H13O2	9	0.1
162.08	C10H10O2	6	0.1
162.11	C11H14O	15	0.2
163.09	C10H11O2	36	0.5
163.12	C11H15O	44	0.6
164.09	C10H12O2	46	0.7
165.10	C10H13O2	566	8.2
166.11	C10H14O2	26	0.4
167.09	C9H11O3	2	0.0
169.07	C8H9O4	5	0.1
169.10	C9H13O3	1	0.0
172.10	C12H12O	4	0.1
174.08	C11H10O2	2	0.0
174.11	C12H14O	6	0.1
175.09	C11H11O2	10	0.1
176.09	C11H12O2	9	0.1
177.10	C11H13O2	3	0.0
177.14	C12H17O	8	0.1
178.08	C10H10O3	2	0.0
178.11	C11H14O2	29	0.4
179.12	C11H15O2	8	0.1
180.09	C10H12O3	3	0.0
182.11	C10H14O3	8	0.1
188.09	C12H12O2	10	0.1
188.13	C13H16O	8	0.1
189.10	C12H13O2	40	0.6
189.14	C13H17O	21	0.3
190.11	C12H14O2	185	2.7
190.14	C13H18O	3	0.0
191.12	C12H15O2	630	9.1
192.13	C12H16O2	40	0.6
193.10	C11H13O3	8	0.1
194.11	C11H14O3	6	0.1
195.08	C10H11O4	11	0.2
195.12	C11H15O3	7	0.1
196.09	C10H12O4	2	0.0
196.13	C11H16O3	4	0.1
202.11	C13H14O2	8	0.1
203.12	C13H15O2	2	0.0
204.13	C13H16O2	25	0.4
**205.13**	**C13H17O2**	**270**	**3.9**
206.14	C13H18O2	1001	14.5
207.12	C12H15O3	4	0.1
207.15	C13H19O2	51	0.7
208.09	C11H12O4	1	0.0
208.13	C12H16O3	8	0.1
209.10	C11H13O4	7	0.1
211.12	C11H15O4	5	0.1
217.10	C13H13O3	2	0.0
220.09	C12H12O4	5	0.1
220.13	C13H16O3	6	0.1
221.10	C12H13O4	32	0.5
222.11	C12H14O4	2	0.0
222.14	C13H18O3	9	0.1
223.12	C12H15O4	46	0.7
223.15	C13H19O3	9	0.1
230.11	C14H14O3	6	0.1
231.12	C14H15O3	1	0.0
232.13	C14H16O3	6	0.1
233.13	C14H17O3	69	1.0
234.11	C13H14O4	35	0.5
234.14	C14H18O3	4	0.1
235.12	C13H15O4	182	2.6
236.12	C13H16O4	1142	16.5
248.12	C14H16O4	6	0.1
249.13	C14H17O4	80	1.2
250.14	C14H18O4	169	2.4
251.15	C14H19O4	7714	

Table S2. Overview of fragments generated in the QCxMS simulation of the Trolox precursor protonated at the carboxylic acid (Figure S10). The exact masses, sum formulas, absolute number of xyz files per fragment and the relative abundance are given.

Exact mass	Sum formula	number	%
1.01	H	197	2.7
2.02	H2	25	0.3
15.02	CH3	71	1.0
16.03	CH4	4	0.1
17.01	HO	2	0.0
18.02	H2O	36	0.5
26.02	C2H2	1	0.0
27.02	C2H3	2	0.0
28.00	CO	30	0.4
28.03	C2H4	6	0.1
29.01	CHO	4	0.1
29.04	C2H5	2	0.0
30.05	C2H6	1	0.0
31.02	CH3O	1	0.0
40.03	C3H4	10	0.1
41.04	C3H5	9	0.1
42.02	C2H2O	2	0.0
42.05	C3H6	10	0.1
43.02	C2H3O	11	0.2
44.00	CO2	40	0.6
44.03	C2H4O	1	0.0
45.01	CHO2	29	0.4
45.04	C2H5O	3	0.0
46.01	CH2O2	92	1.3
47.02	CH3O2	7	0.1
52.03	C4H4	1	0.0
53.04	C4H5	13	0.2
54.02	C3H2O	1	0.0
54.05	C4H6	4	0.1
55.02	C3H3O	15	0.2
55.06	C4H7	8	0.1
56.03	C3H4O	9	0.1
56.06	C4H8	6	0.1
60.03	C2H4O2	2	0.0
61.04	C2H5O2	13	0.2
62.01	CH2O3	3	0.0
66.02	C4H2O	1	0.0
67.02	C4H3O	2	0.0
68.03	C4H4O	1	0.0
69.04	C4H5O	2	0.0
70.05	C4H6O	2	0.0
71.02	C3H3O2	2	0.0
72.03	C3H4O2	10	0.1
73.04	C3H5O2	28	0.4
74.05	C3H6O2	7	0.1
77.04	C6H5	4	0.1
80.03	C5H4O	2	0.0
81.01	C4HO2	1	0.0
81.04	C5H5O	5	0.1
82.05	C5H6O	6	0.1
83.05	C5H7O	8	0.1
84.03	C4H4O2	1	0.0
85.04	C4H5O2	5	0.1
86.05	C4H6O2	221	3.1
87.05	C4H7O2	402	5.6
88.03	C3H4O3	4	0.1
89.04	C3H5O3	2	0.0
95.05	C6H7O	4	0.1
96.06	C6H8O	9	0.1
97.04	C5H5O2	9	0.1
97.07	C6H9O	3	0.0
98.05	C5H6O2	1	0.0
99.05	C5H7O2	50	0.7
100.06	C5H8O2	30	0.4
101.04	C4H5O3	1	0.0
101.07	C5H9O2	11	0.2
102.04	C4H6O3	1	0.0
103.05	C4H7O3	6	0.1
107.05	C7H7O	6	0.1
109.04	C6H5O2	2	0.0
109.07	C7H9O	2	0.0
110.05	C6H6O2	8	0.1
111.05	C6H7O2	7	0.1
111.09	C7H11O	4	0.1
112.06	C6H8O2	12	0.2
119.09	C9H11	2	0.0
120.10	C9H12	2	0.0
121.10	C9H13	5	0.1
122.05	C7H6O2	1	0.0
122.08	C8H10O	9	0.1
123.05	C7H7O2	4	0.1
123.09	C8H11O	17	0.2
124.06	C7H8O2	2	0.0
126.04	C6H6O3	1	0.0
126.08	C7H10O2	3	0.0
133.07	C9H9O	5	0.1
134.05	C8H6O2	1	0.0

134.08	C9H10O	2	0.0
135.05	C8H7O2	14	0.2
135.09	C9H11O	6	0.1
136.06	C8H8O2	2	0.0
136.10	C9H12O	49	0.7
137.10	C9H13O	35	0.5
138.08	C8H10O2	10	0.1
139.09	C8H11O2	14	0.2
140.09	C8H12O2	8	0.1
147.05	C9H7O2	1	0.0
147.09	C10H11O	2	0.0
148.10	C10H12O	1	0.0
149.07	C9H9O2	22	0.3
149.10	C10H13O	14	0.2
150.08	C9H10O2	60	0.8
150.11	C10H14O	4	0.1
151.09	C9H11O2	164	2.3
152.09	C9H12O2	131	1.8
153.10	C9H13O2	4	0.1
161.10	C11H13O	10	0.1
162.08	C10H10O2	6	0.1
162.11	C11H14O	24	0.3
163.09	C10H11O2	24	0.3
163.12	C11H15O	16	0.2
164.09	C10H12O2	310	4.3
165.07	C9H9O3	2	0.0
165.10	C10H13O2	1432	19.9
166.11	C10H14O2	14	0.2
174.11	C12H14O	1	0.0
175.09	C11H11O2	6	0.1
176.09	C11H12O2	26	0.4
176.13	C12H16O	8	0.1
177.10	C11H13O2	46	0.6
177.14	C12H17O	8	0.1
178.08	C10H10O3	2	0.0
178.11	C11H14O2	151	2.1
178.14	C12H18O	3	0.0
179.12	C11H15O2	23	0.3
180.13	C11H16O2	12	0.2
187.12	C13H15O	12	0.2
188.13	C13H16O	8	0.1
189.10	C12H13O2	6	0.1
189.14	C13H17O	20	0.3
190.11	C12H14O2	41	0.6
191.12	C12H15O2	12	0.2
192.13	C12H16O2	14	0.2
193.10	C11H13O3	5	0.1
195.12	C11H15O3	18	0.2
196.13	C11H16O3	3	0.0
199.09	C13H11O2	6	0.1
200.09	C13H12O2	5	0.1
202.08	C12H10O3	8	0.1
202.11	C13H14O2	8	0.1
203.12	C13H15O2	24	0.3
204.13	C13H16O2	85	1.2
**205.13**	**C13H17O2**	**1059**	**14.7**
206.14	C13H18O2	235	3.3
207.08	C11H11O4	6	0.1
207.15	C13H19O2	19	0.3
209.10	C11H13O4	5	0.1
215.12	C14H15O2	4	0.1
217.10	C13H13O3	1	0.0
218.11	C13H14O3	7	0.1
219.08	C12H11O4	4	0.1
221.13	C13H17O3	4	0.1
222.14	C13H18O3	3	0.0
223.12	C12H15O4	28	0.4
223.15	C13H19O3	41	0.6
232.13	C14H16O3	5	0.1
233.13	C14H17O3	193	2.7
234.11	C13H14O4	13	0.2
234.14	C14H18O3	14	0.2
235.12	C13H15O4	60	0.8
236.12	C13H16O4	445	6.2
248.12	C14H16O4	19	0.3
249.13	C14H17O4	195	2.7
250.14	C14H18O4	299	4.1
251.15	C14H19O4	9790	

Negative Ion Mode: Decarboxylation of Trolox

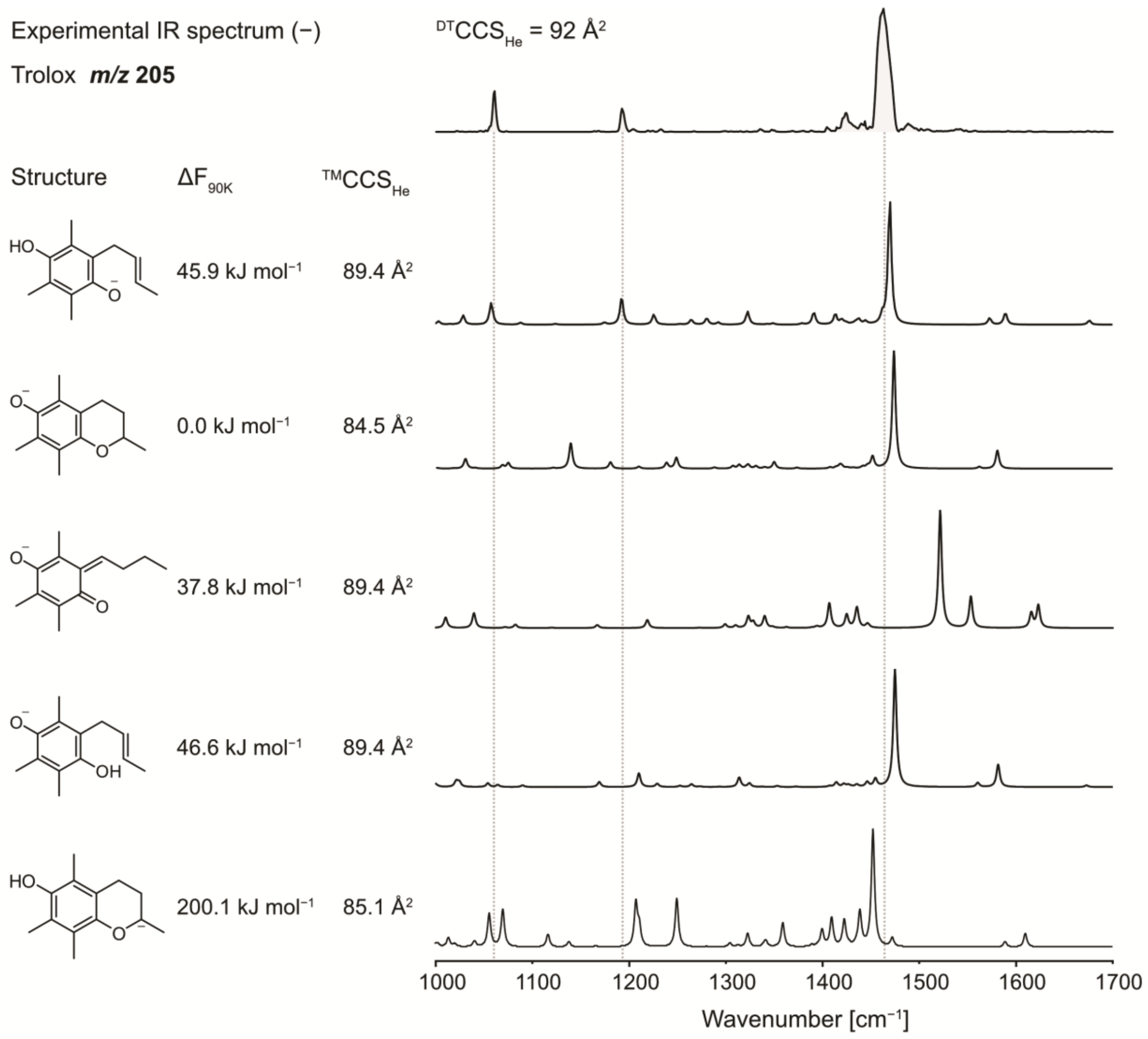

Figure S11. Experimental IR spectrum of the fragment at *m/z* 205 generated from deprotonated Trolox precursor ions and computed IR spectra of candidate structures. The band at 1200 cm^{-1} indicates the presence of a hydroxyl group, whereas the position of the main band corresponds to the frequency of a phenolic C–O stretch. The best spectral match is shown on top and does not correspond to the lowest-energy structure. Spectra were computed at the PBE0+D3/6-311+G(d,p) level of theory and scaled by 0.965. XYZ coordinates of all structures are listed on page 44ff.

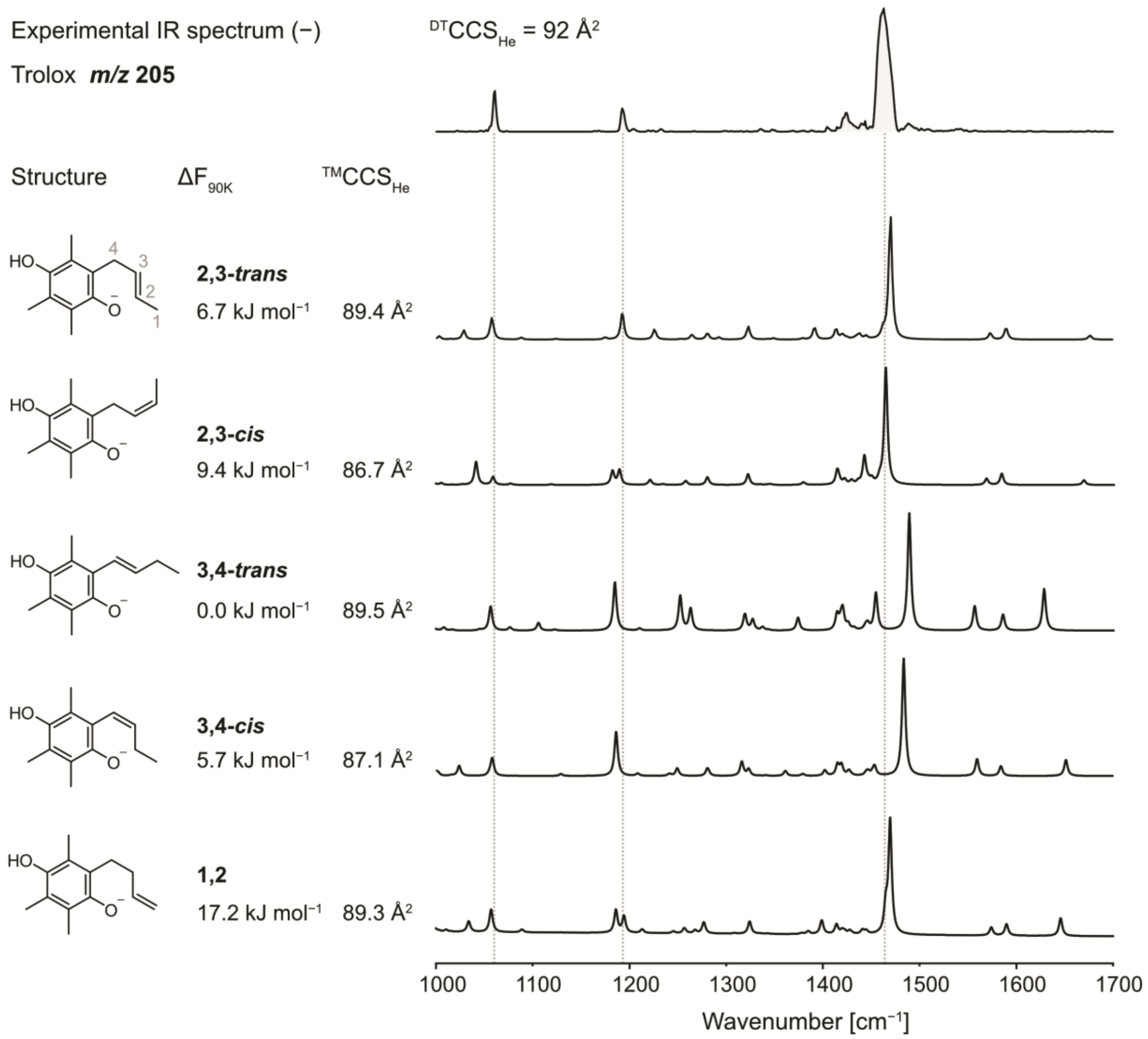

Figure S12. Experimental IR spectrum of the fragment at *m/z* 205 generated from deprotonated Trolox precursor ions and computed IR spectra of candidate structures featuring varying positions and configurations of the C=C bond in the aliphatic chain. The 3,4-isomers can be excluded both due to their poor spectral match and incompatibility with the proposed reaction pathway (*cf.* Figure S13). The 2,3- and 1,2 double bond isomers yield a satisfactory spectral match. Spectra were computed at the PBE0+D3/6-311+G(d,p) level of theory and scaled by 0.965. XYZ coordinates of all structures are listed on page 45ff.

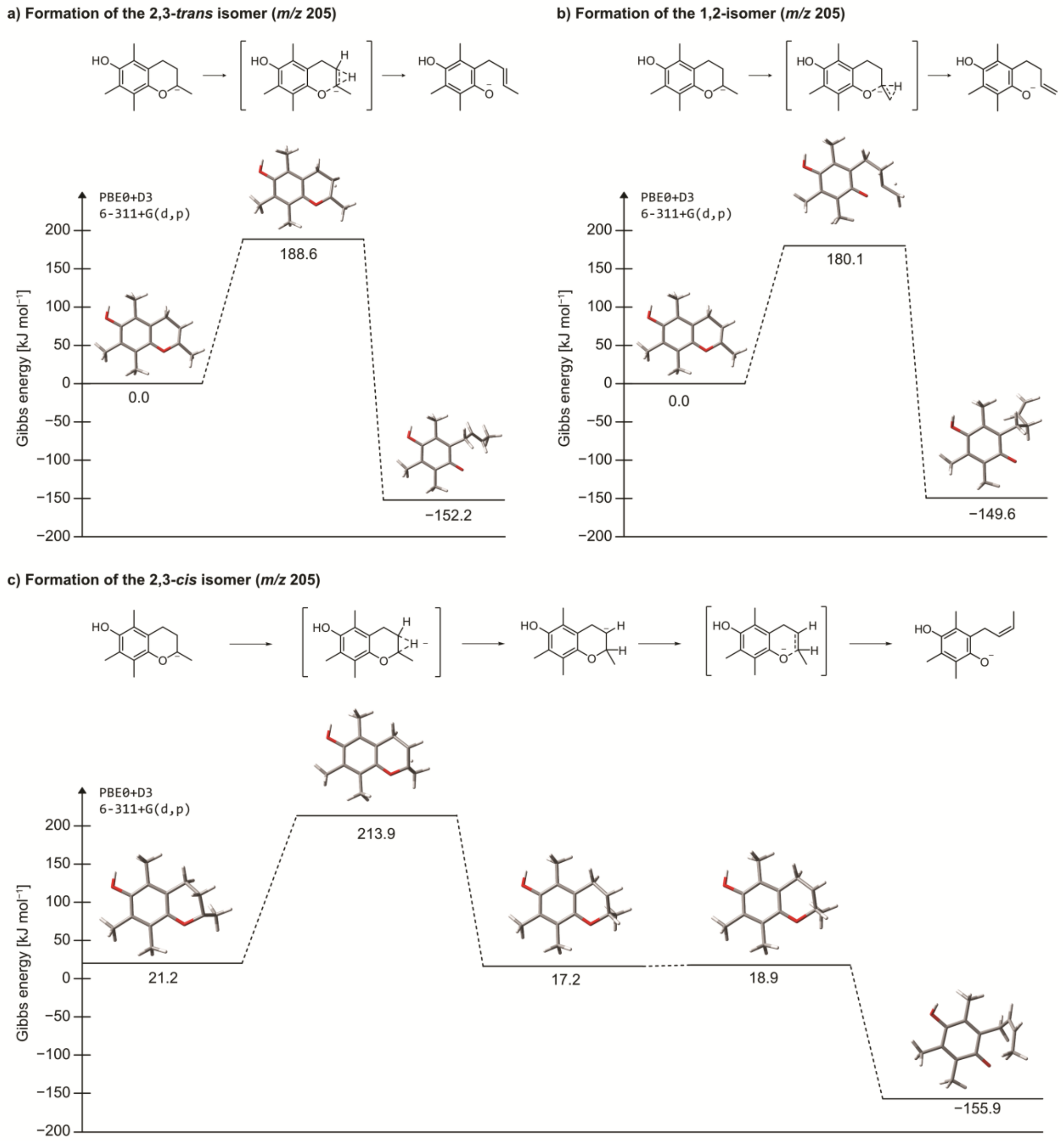

Figure S13. Computed reaction pathway for the intramolecular ring opening of deprotonated Trolox after decarboxylation (*m/z* 205) in negative ion mode. Computed energy profiles for the formation of the 2,3-*trans* (a), 1,2- (b) and 2,3-*cis* (c) double bond isomers are shown. According to the computation, the reaction involves an initial proton transfer, followed by a ring opening reaction with no significant activation barrier, which yields the spectroscopically observed fragments at *m/z* 205. The energy profiles for the formation of the 2,3-*trans* and 1,2-isomer start from the same initial structure. The formation of the 2,3-*cis* isomer requires a different ring conformation of the initial structure, which is 21 kJ mol^{-1} higher in energy (ΔG). The structures were optimized at the PBE0+D3/6-311+G(d,p) level of theory. XYZ coordinates of all structures are listed on page 47ff.

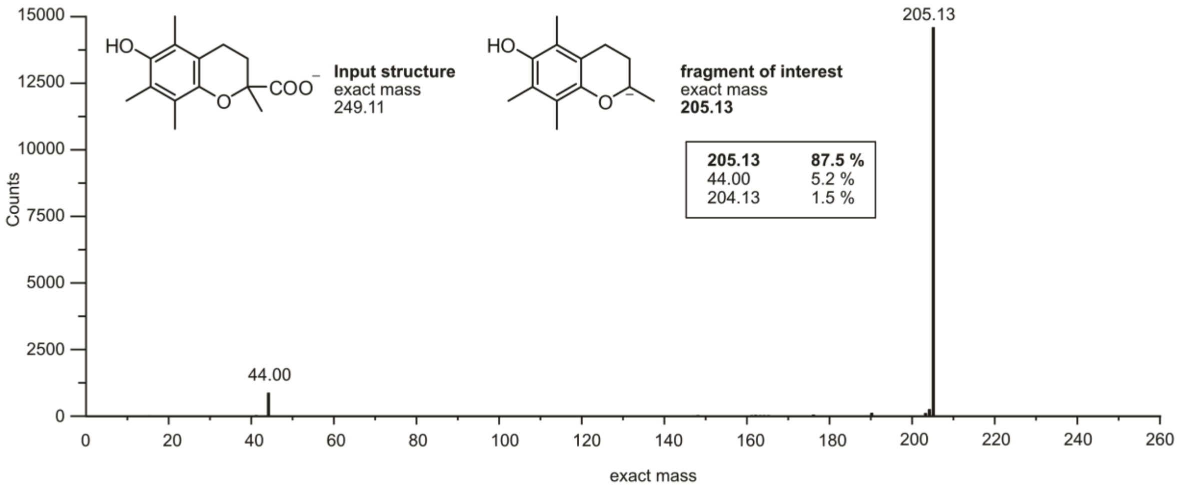

Figure S14. Simulation of collision-induced dissociation of the depicted deprotonated Trolox precursor in QCxMS. The exact masses of all generated structures were extracted and plotted against their abundance, *i.e.,* the absolute number of xyz output files corresponding to the respective exact mass. The relative abundances of the most abundant fragments are shown and refer to the sum of all generated fragments (excluding the precursor). The fragment of interest (exact mass = 205.13) is the most abundant fragment and corresponds to the depicted structure. The precursor dissociates completely.

Table S3. Overview of fragments generated in the QCxMS simulation of the deprotonated Trolox precursor (Figure S14). The exact masses, sum formulas, absolute number of xyz files per fragment and the relative abundance are given.

Exact mass	Sum formula	number	%
1.01	H	77	0.5
2.02	H2	10	0.1
15.02	CH3	27	0.2
16.03	CH4	2	0.0
17.01	HO	1	0.0
18.02	H2O	1	0.0
27.02	C2H3	2	0.0
28.00	CO	8	0.0
28.03	C2H4	3	0.0
29.04	C2H5	9	0.1
40.03	C3H4	9	0.1
41.01	C2HO	1	0.0
41.04	C3H5	30	0.2
42.02	C2H2O	7	0.0
42.05	C3H6	3	0.0
43.02	C2H3O	25	0.1
44.00	CO2	876	5.2
44.03	C2H4O	6	0.0
54.02	C3H2O	1	0.0
54.05	C4H6	3	0.0
55.06	C4H7	5	0.0
56.03	C3H4O	6	0.0
57.04	C3H5O	5	0.0
58.05	C3H6O	5	0.0
67.06	C5H7	4	0.0
70.05	C4H6O	4	0.0
71.05	C4H7O	1	0.0
77.04	C6H5	2	0.0
82.05	C5H6O	2	0.0
95.05	C6H7O	1	0.0
108.06	C7H8O	4	0.0
109.07	C7H9O	4	0.0
110.08	C7H10O	2	0.0
121.07	C8H9O	2	0.0
123.09	C8H11O	7	0.0
132.06	C9H8O	2	0.0
133.07	C9H9O	12	0.1
134.08	C9H10O	1	0.0
135.09	C9H11O	12	0.1
137.10	C9H13O	4	0.0
138.08	C8H10O2	1	0.0
145.10	C11H13	6	0.0
146.08	C10H10O	1	0.0
147.09	C10H11O	20	0.1
148.09	C10H12O	31	0.2
149.10	C10H13O	12	0.1
150.08	C9H10O2	9	0.1
151.09	C9H11O2	8	0.0
159.09	C11H11O	6	0.0
161.10	C11H13O	32	0.2
162.11	C11H14O	39	0.2
163.09	C10H11O2	7	0.0
163.12	C11H15O	34	0.2
164.09	C10H12O2	35	0.2
165.10	C10H13O2	36	0.2
174.11	C12H14O	2	0.0
175.09	C11H11O2	2	0.0
175.12	C12H15O	10	0.1
176.09	C11H12O2	47	0.3
177.10	C11H13O2	19	0.1
177.14	C12H17O	10	0.1
187.09	C12H11O2	6	0.0
187.12	C13H15O	3	0.0
188.13	C13H16O	4	0.0
189.10	C12H13O2	26	0.2
190.11	C12H14O2	116	0.7
202.11	C13H14O2	10	0.1
203.12	C13H15O2	114	0.7
204.13	C13H16O2	258	1.5
**205.13**	**C13H17O2**	**14596**	**87.5**

Negative Ion Mode: Radical Fragmentation of Methyltrolox

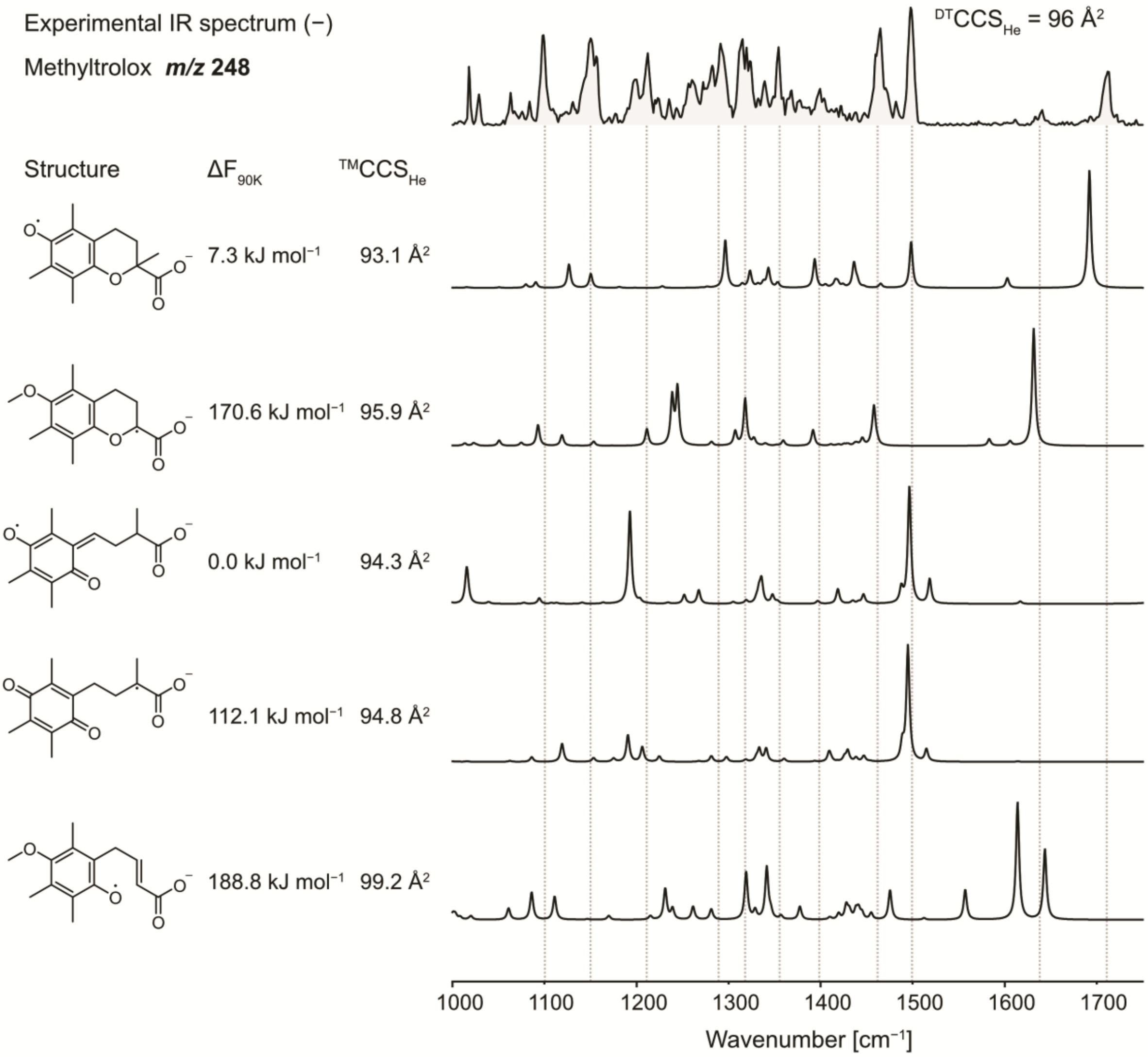

Figure S15. Experimental IR spectrum of the radical fragment at *m/z* 248 generated by methyl radical abstraction from deprotonated Methyltrolox precursor ions, and computed IR spectra of candidate structures. The highly populated experimental spectrum suggests that methyl radicals could be abstracted from different positions. The best spectral match is shown on top and corresponds to a phenol radical. Spectra were computed at the PBE0+D3/6-311+G(d,p) level of theory and scaled by 0.965. XYZ coordinates of all structures are listed on page 50ff.

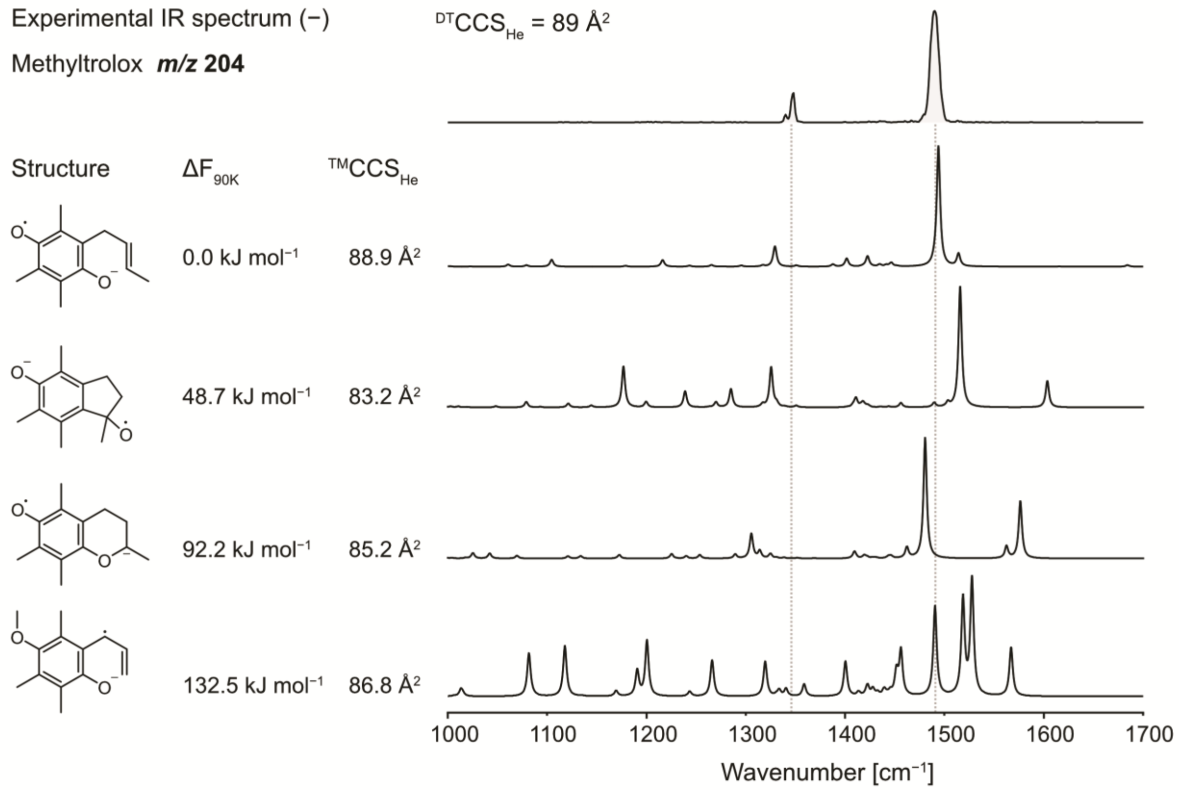

Figure S16. Experimental IR spectrum of the radical fragment at *m/z* 204 generated from deprotonated Methyltrolox precursor ions and computed IR spectra of candidate structures. The band at 1500 cm^{-1} coincides with the frequency of a phenolic C–O stretching vibration. The best spectral match is shown on top and corresponds to the most stable structure in which the radical and charge are delocalized in the aromatic ring. Spectra were computed at the PBE0+D3/6-311+G(d,p) level of theory and scaled by 0.965. XYZ coordinates of all structures are listed on page 52f.

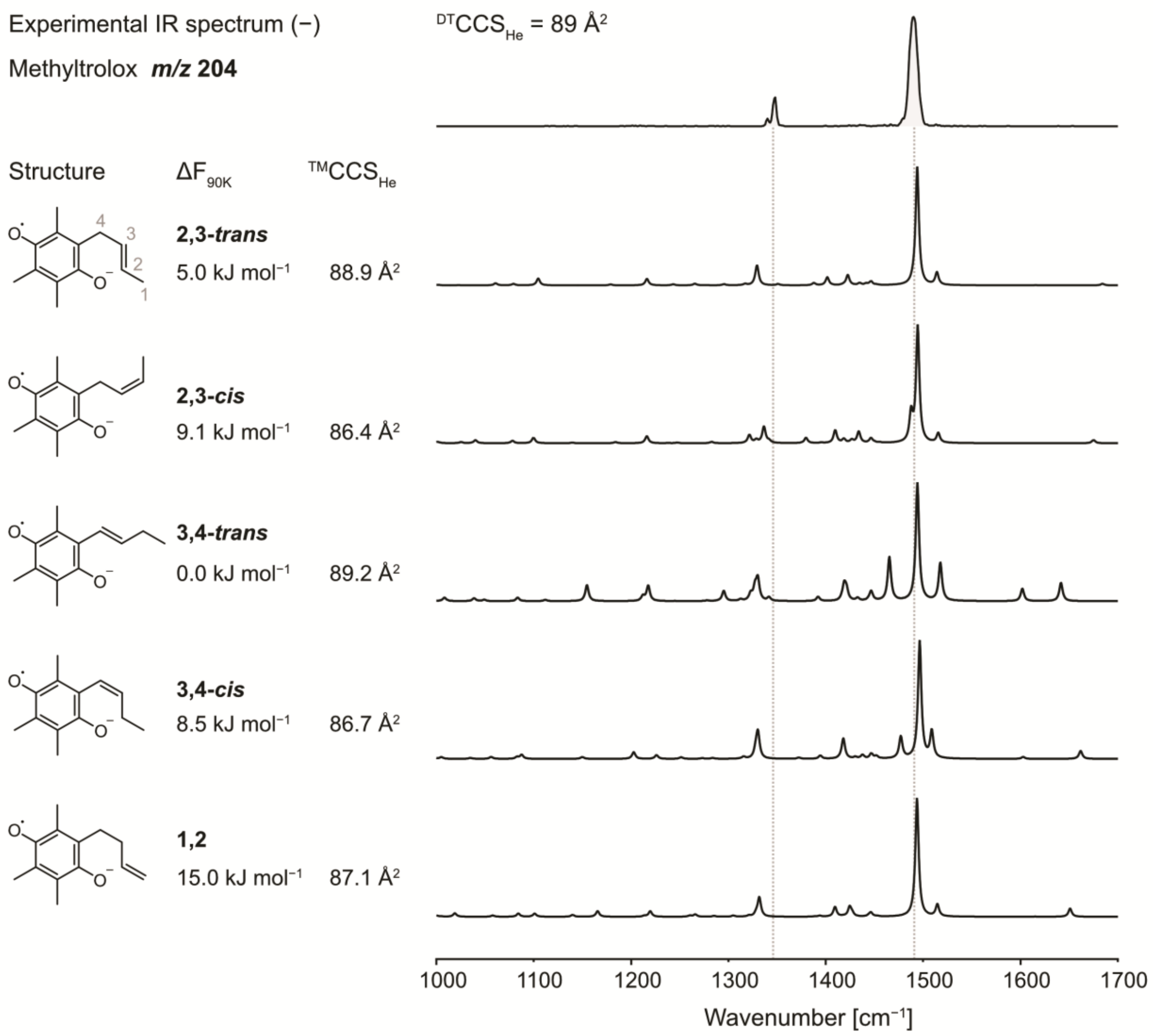

Figure S17. Experimental IR spectrum of the radical fragment at *m/z* 204 generated from deprotonated Methyltrolox precursor ions and computed IR spectra of candidate structures featuring varying positions and configurations of the C=C bond in the aliphatic chain. The best spectral match is obtained for the 2,3- and 1,2 double bond isomers, which can be formed via the fragmentation reaction shown in *Figure S18*. Spectra were computed at the PBE0+D3/6-311+G(d,p) level of theory and scaled by 0.965. XYZ coordinates of all structures are listed on page 53ff.

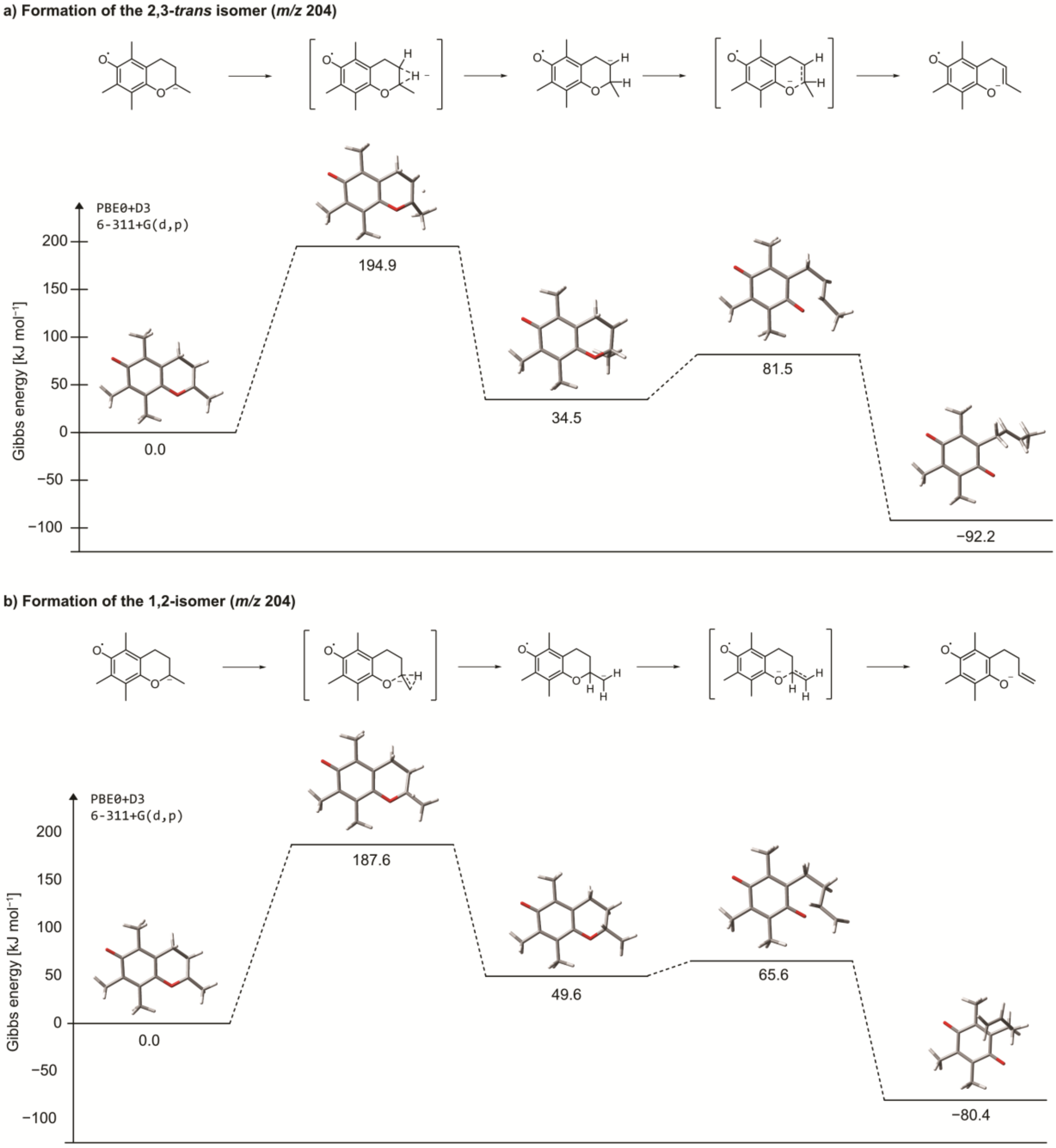

Figure S18. Computed reaction pathway for the intramolecular ring opening of deprotonated, decarboxylated and demethylated Methyltrolox (*m/z* 204) yielding the spectroscopically observed fragment at *m/z* 204 in negative ion mode. Computed energy profiles for the formation of the 2,3-*trans* (a), and 1,2- (b) double bond isomers are shown. According to the computation, the reaction involves an initial proton transfer, followed by a ring opening reaction. The activation barriers are similar for both isomers. The structures were optimized at the PBE0+D3/6-311+G(d,p) level of theory. XYZ coordinates of all structures are listed on page 55ff.

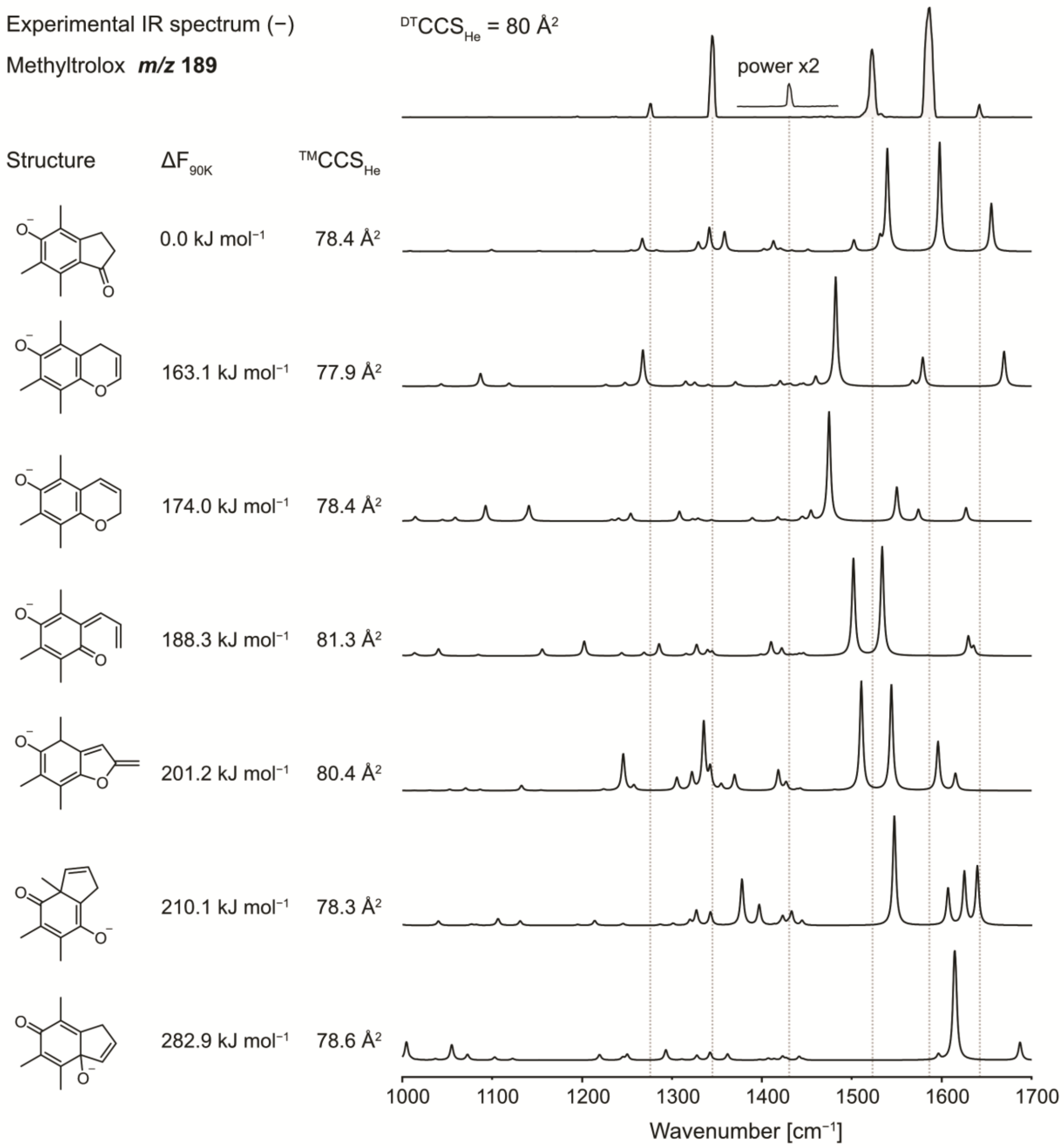

Figure S19. Experimental IR spectrum of the even-electron fragment at *m/z* 189 generated from deprotonated Methyltrolox precursor ions and computed IR spectra of candidate structures. The best spectral match is shown on top and corresponds to a rearranged structure featuring a cyclopentanone ring. Increasing the laser energy by 100 % renders an additional band at 1420 cm^{-1} visible but leads to oversaturation of the rest of the spectrum. Spectra were computed at the PBE0+D3/6-311+G(d,p) level of theory and scaled by 0.965. XYZ coordinates of all structures are listed on page 57ff.

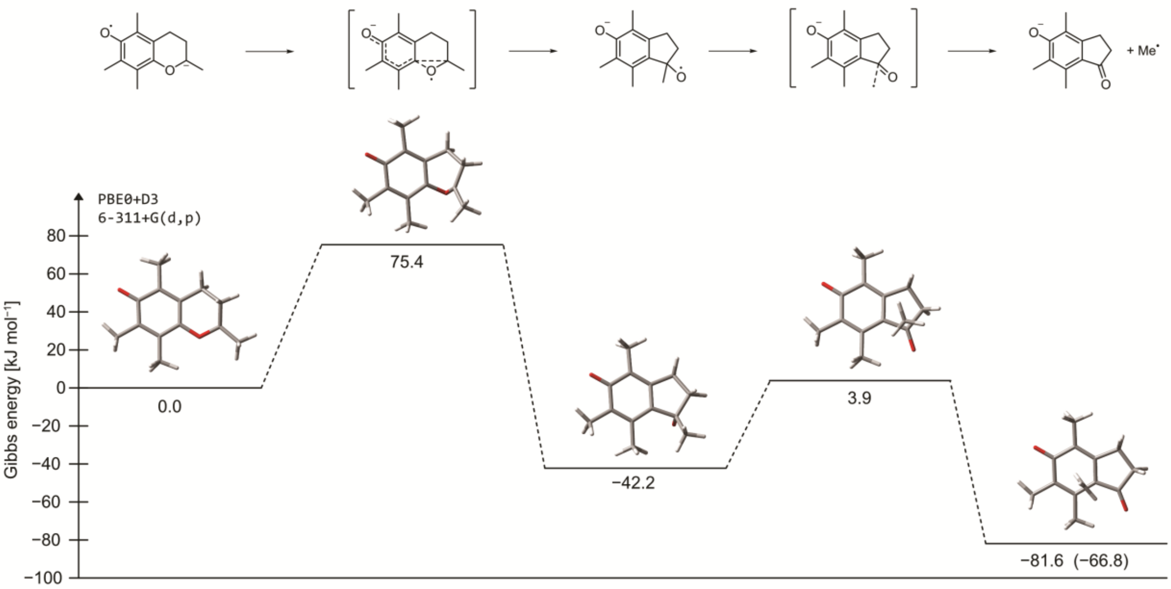

Figure S20. Computed reaction pathway leading from decarboxylated and demethylated Methyltrolox (*m/z* 204) to the spectroscopically observed fragment at *m/z* 189 in negative ion mode. According to the computation, the reaction involves a ring size reduction, followed by dissociation of a methyl radical. The Gibbs energy of the non-separated products is indicated in brackets. The structures were optimized at the PBE0+D3/6-311+G(d,p) level of theory. XYZ coordinates of all structures are listed on page 59ff.

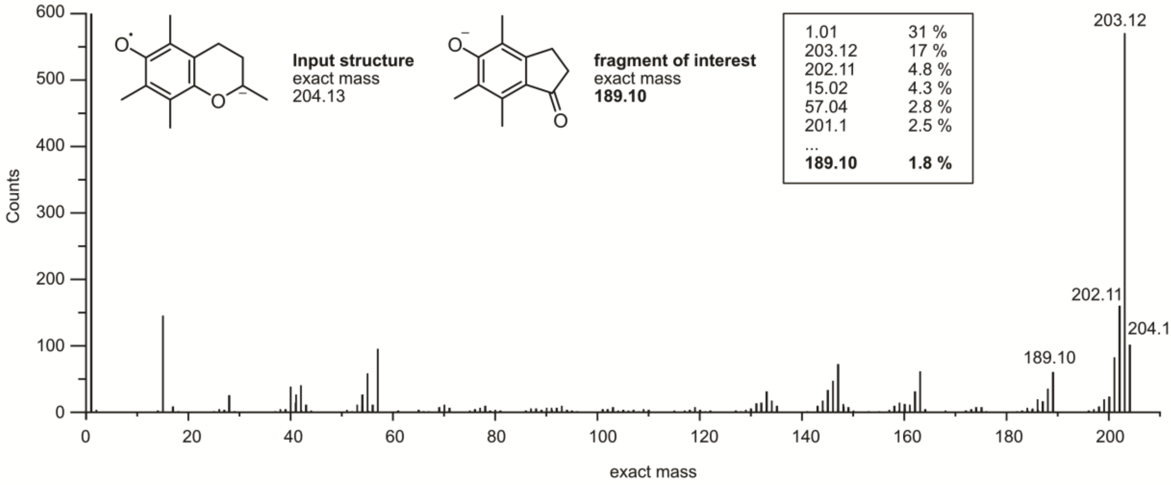

Figure S21. Simulation of the unimolecular dissociation of the depicted radical anion after dissociative electron attachment in QCxMS. The exact masses of all generated structures were extracted and plotted against their abundance, *i.e.,* the absolute number of xyz output files corresponding to the respective exact mass. The relative abundances of the most abundant fragments are shown and refer to the sum of all generated fragments (excluding the precursor). The fragment of interest (exact mass = 189.10) is among the most abundant fragments apart from the dominant hydrogen abstraction.

Table S4. Overview of fragments generated in the QCxMS simulation of the Methyltrolox radical anion at *m/z* 204 (Figure S21). The exact masses, sum formulas, absolute number of xyz files per fragment and the relative abundance are given.

Exact mass	Sum formula	number	%
1.01	H	1038	31.1
2.02	H2	3	0.1
14.02	CH2	2	0.1
15.02	CH3	145	4.3
17.01	HO	8	0.2
18.02	H2O	1	0.0
25.01	C2H	1	0.0
26.02	C2H2	4	0.1
27.02	C2H3	3	0.1
28.00	CO	25	0.7
28.03	C2H4	18	0.5
29.01	CHO	1	0.0
29.04	C2H5	1	0.0
37.01	C3H	1	0.0
38.02	C3H2	4	0.1
39.02	C3H3	4	0.1
40.00	C2O	1	0.0
40.03	C3H4	38	1.1
41.01	C2HO	14	0.4
41.04	C3H5	26	0.8
42.02	C2H2O	40	1.2
42.05	C3H6	2	0.1
43.02	C2H3O	11	0.3
44.00	CO2	1	0.0
44.03	C2H4O	2	0.1
51.02	C4H3	3	0.1
52.03	C4H4	1	0.0
53.01	C3HO	1	0.0
53.04	C4H5	11	0.3
54.02	C3H2O	26	0.8
54.05	C4H6	10	0.3
55.02	C3H3O	58	1.7
55.06	C4H7	2	0.1
56.00	C2O2	2	0.1
56.03	C3H4O	11	0.3
57.04	C3H5O	95	2.8
61.01	C5H	2	0.1
65.04	C5H5	3	0.1
66.02	C4H2O	1	0.0
67.02	C4H3O	1	0.0
69.04	C4H5O	7	0.2
70.05	C4H6O	11	0.3
71.05	C4H7O	6	0.2
75.02	C6H3	2	0.1
76.03	C6H4	4	0.1
77.04	C6H5	6	0.2
78.01	C5H2O	2	0.1
78.02	C5H2O	1	0.0
78.05	C6H6	9	0.3
79.02	C5H3O	2	0.1
79.06	C6H7	1	0.0
80.03	C5H4O	3	0.1
81.04	C5H5O	2	0.1
86.02	C7H2	2	0.1
87.02	C7H3	5	0.1
88.03	C7H4	5	0.1
89.04	C7H5	3	0.1
90.05	C7H6	6	0.2
91.02	C6H3O	6	0.2
91.06	C7H7	3	0.1
92.00	C5O2	2	0.1
92.03	C6H4O	6	0.2
92.06	C7H8	4	0.1
93.04	C6H5O	9	0.3
94.05	C6H6O	3	0.1
95.02	C5H3O2	2	0.1
96.06	C6H8O	1	0.0
101.04	C8H5	4	0.1
102.01	C7H2O	4	0.1
102.02	C7H2O	2	0.1
102.05	C8H6	2	0.1
103.02	C7H3O	7	0.2
103.06	C8H7	3	0.1
104.03	C7H4O	1	0.0
104.06	C8H8	2	0.1
105.04	C7H5O	2	0.1
105.07	C8H9	3	0.1
106.05	C7H6O	2	0.1
106.08	C8H10	1	0.0
107.05	C7H7O	3	0.1
108.06	C7H8O	1	0.0
109.04	C6H5O2	4	0.1
109.07	C7H9O	1	0.0
110.05	C6H6O2	3	0.1
115.02	C8H3O	1	0.0
115.06	C9H7	2	0.1
117.04	C8H5O	2	0.1

117.07	C9H9	1	0.0
118.05	C8H6O	3	0.1
119.05	C8H7O	7	0.2
120.03	C7H4O2	1	0.0
120.06	C8H8O	3	0.1
121.07	C8H9O	1	0.0
122.08	C8H10O	2	0.1
127.06	C10H7	2	0.1
128.06	C10H8	1	0.0
129.04	C9H5O	3	0.1
130.05	C9H6O	5	0.1
130.08	C10H10	1	0.0
131.02	C8H3O2	2	0.1
131.06	C9H7O	13	0.4
132.03	C8H4O2	1	0.0
132.06	C9H8O	14	0.4
133.04	C8H5O2	4	0.1
133.07	C9H9O	31	0.9
134.05	C8H6O2	1	0.0
134.08	C9H10O	17	0.5
135.09	C9H11O	9	0.3
141.04	C10H5O	1	0.0
143.06	C10H7O	9	0.3
143.09	C11H11	1	0.0
144.06	C10H8O	17	0.5
144.10	C11H12	2	0.1
145.04	C9H5O2	2	0.1
145.07	C10H9O	33	1.0
146.05	C9H6O2	2	0.1
146.08	C10H10O	47	1.4
147.05	C9H7O2	4	0.1
147.09	C10H11O	72	2.2
148.06	C9H8O2	12	0.4
148.10	C10H12O	8	0.2
149.07	C9H9O2	7	0.2
150.08	C9H10O2	2	0.1
153.04	C11H5O	1	0.0
155.06	C11H7O	1	0.0
157.07	C11H9O	3	0.1
158.08	C11H10O	9	0.3
159.05	C10H7O2	2	0.1
159.09	C11H11O	14	0.4
160.06	C10H8O2	5	0.1
160.10	C11H12O	12	0.4
161.07	C10H9O2	6	0.2
161.10	C11H13O	11	0.3
162.08	C10H10O2	31	0.9
162.11	C11H14O	1	0.0
163.09	C10H11O2	61	1.8
164.09	C10H12O2	4	0.1
168.06	C12H8O	2	0.1
172.06	C11H8O2	2	0.1
172.10	C12H12O	2	0.1
173.07	C11H9O2	3	0.1
173.10	C12H13O	4	0.1
174.08	C11H10O2	7	0.2
174.11	C12H14O	2	0.1
175.09	C11H11O2	7	0.2
175.12	C12H15O	2	0.1
176.09	C11H12O2	1	0.0
182.05	C12H6O2	1	0.0
183.05	C12H7O2	2	0.1
183.09	C13H11O	1	0.0
184.06	C12H8O2	6	0.2
184.10	C13H12O	2	0.1
185.07	C12H9O2	4	0.1
185.10	C13H13O	5	0.1
186.08	C12H10O2	19	0.6
186.11	C13H14O	3	0.1
187.09	C12H11O2	16	0.5
187.12	C13H15O	1	0.0
188.09	C12H12O2	35	1.0
**189.10**	**C12H13O2**	**60**	**1.8**
196.06	C13H8O2	2	0.1
197.07	C13H9O2	4	0.1
198.08	C13H10O2	8	0.2
199.09	C13H11O2	19	0.6
200.09	C13H12O2	23	0.7
201.10	C13H13O2	82	2.5
202.11	C13H14O2	160	4.8
203.12	C13H15O2	570	17.1
204.13	C13H16O2	101	

Collision Cross Sections

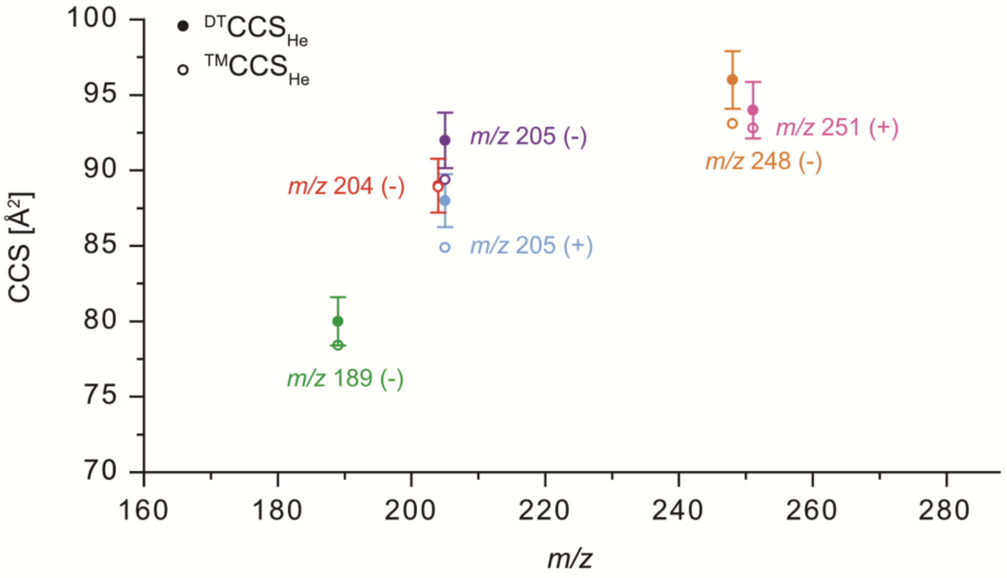

Figure S22. Comparison between experimental drift tube (DT) and computed trajectory method (TM) collision cross sections of ions derived from Trolox and Methyltrolox. The computed cross sections of the structures with the best spectral match are shown, respectively. Collision cross sections were measured in helium at a pressure of 1.8 Torr. The error of the measurement is estimated to be 2 % of the absolute value.

Comparison with Structure Prediction

Table S5. Comparison between the fragment structures identified in this work and structure predictions generated by CFM-ID 4.0. Fragments that are not listed were not generated during *in silico* fragmentation.

	**IR spectroscopy**	**CFM-ID 4.0**
Trolox $[M+H]^+$<br><br>fragment<br>*m/z* 205	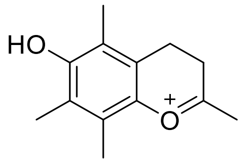	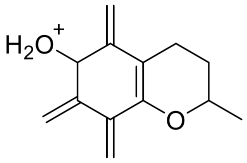
Trolox $[M-H]^-$<br><br>fragment<br>*m/z* 205	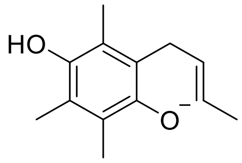	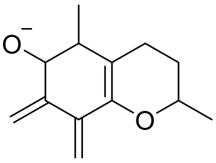
Methyltrolox $[M-H]^-$<br><br>fragment<br>*m/z* 189	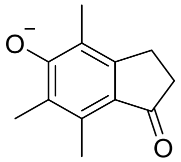	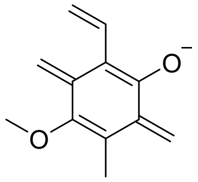

Coordinates of Computed Structures

XYZ coordinates of all geometry-optimized structures shown in this work are listed below in their order of appearance. Each xyz file is linked to the corresponding figure and identifiable by its relative free energy (ΔF_{90K}) or Gibbs energy (ΔG). Charge and multiplicity are indicated ahead of the coordinates, *e.g.,* −1 1 for a negatively charged singlet. The structures were optimized at the PBE0+D3/6-311+G(d,p) level of theory.

Trolox *m/z* 249 (−)

Figure S3

0.0 kJ mol^{-1} (ΔF_{90K})

```
-1 1
C -1.548875 -1.383176 -0.094572
C -0.255351 -0.900777 -0.333207
C -0.050899 0.480412 -0.367386
C -1.107990 1.379887 -0.178490
C -2.403062 0.898339 0.018295
C -2.600884 -0.482140 0.058705
O -3.890071 -0.936144 0.267840
H -3.860520 -1.893100 0.313360
C -3.568374 1.828003 0.211239
H -4.505941 1.272473 0.229762
H -3.624350 2.569751 -0.592207
H -3.486726 2.378998 1.155673
C -0.777977 2.841333 -0.160662
H 0.115471 2.996107 0.450092
H -0.540028 3.211136 -1.165655
H -1.596641 3.441748 0.239602
O 1.154169 1.041181 -0.616032
C 2.318593 0.226609 -0.407690
C 2.599986 -0.024528 1.125329
O 3.514891 -0.855526 1.316634
O 1.915822 0.620308 1.932643
C 2.108979 -1.092436 -1.132244
C 0.917347 -1.824567 -0.533519
H 0.635343 -2.664855 -1.178956
H 1.223743 -2.246591 0.432390
H 1.936609 -0.886147 -2.198029
H 3.013341 -1.693715 -1.026561
C 3.464790 1.021464 -0.998284
H 3.323491 1.170057 -2.075924
H 4.397560 0.483883 -0.818162
H 3.523193 2.000189 -0.513938
C -1.830467 -2.856699 0.019589
H -2.390106 -3.090638 0.936371
H -2.408370 -3.242407 -0.832512
H -0.909455 -3.435794 0.073388
```

17.7 kJ mol^{-1}

```
-1 1
C 1.897238 -1.330023 -0.113365
C 2.930858 -0.331600 -0.029813
C 2.475951 1.037206 0.022889
C 1.123500 1.369514 0.007555
C 0.172392 0.353400 -0.094329
C 0.545741 -0.987440 -0.157638
C -0.506209 -2.065033 -0.220139
C -1.890295 -1.515232 -0.530593
C -2.137647 -0.220675 0.248061
O -1.181226 0.745798 -0.152386
C -3.490914 0.346919 -0.185363
O -4.543238 -0.156752 0.109270
O -3.405041 1.437564 -0.946798
H -2.443757 1.622265 -1.004313
C -2.114574 -0.416926 1.757836
H -2.875356 -1.141855 2.053664
H -2.315567 0.531850 2.261658
H -1.127670 -0.767123 2.066543
H -2.679756 -2.235235 -0.297787
H -1.964377 -1.275150 -1.597708
H -0.527021 -2.615397 0.731582
H -0.235655 -2.808801 -0.977665
C 0.686972 2.806768 0.084680
H 0.805530 3.318071 -0.880311
H -0.361724 2.889230 0.377436
H 1.290611 3.360220 0.810278
C 3.531891 2.098614 0.103185
H 3.537091 2.612850 1.075692
H 3.408578 2.872189 -0.666215
H 4.502101 1.612989 -0.025606
O 4.163644 -0.633995 0.000857
C 2.331639 -2.764041 -0.148986
H 2.059409 -3.264954 -1.089878
H 3.420040 -2.781411 -0.054264
H 1.895154 -3.359371 0.665464
```

Trolox *m/z* 251 (+)

Figure S3 and Figure S5

0.0 kJ mol^{-1} (ΔF_{90K})

```
1 1
C 1.546558 1.352123 0.025065
C 0.248961 0.925597 -0.258514
C 0.046296 -0.434568 -0.461064
O -1.116179 -0.970375 -0.735662
C -2.325617 -0.193028 -0.566948
C -3.404486 -0.936198 -1.323984
H -4.359276 -0.423827 -1.203682
H -3.506066 -1.957561 -0.951729
H -3.149579 -0.971099 -2.385011
C -2.086091 1.219757 -1.082000
H -1.887393 1.146667 -2.155998
C -0.914269 1.874371 -0.362852
```

H -1.212607 2.201353 0.640690
H -0.617689 2.773417 -0.907630
H -2.998045 1.805633 -0.955176
C -2.591733 -0.159199 0.941700
O -1.793470 -0.486562 1.775235
O -3.805011 0.322418 1.196108
H -3.932067 0.344558 2.155468
C 1.133873 -1.433204 -0.372095
C 2.500469 -0.881410 -0.218471
C 2.659122 0.445115 0.025058
O 3.851692 1.037118 0.255734
H 4.558465 0.386832 0.294383
C 3.661276 -1.816260 -0.271292
H 3.380043 -2.783889 -0.688873
H 4.466215 -1.416025 -0.898401
H 4.077683 -2.000364 0.727567
H 1.092159 -2.044514 -1.285252
C 0.817324 -2.383239 0.821208
H 0.813934 -1.822423 1.756698
H 1.568049 -3.172176 0.872458
H -0.165181 -2.830856 0.676456
C 1.852733 2.774539 0.310318
H 0.964045 3.388213 0.431947
H 2.475533 2.843198 1.206833
H 2.469015 3.185688 -0.498857

11.4 kJ mol^{-1}
1 1
C -1.567088 -1.492558 0.215951
C -0.218840 -1.000401 -0.152893
C -0.042358 0.313504 -0.443698
C -1.113370 1.263758 -0.349770
C -2.406212 0.883779 0.033296
C -2.594423 -0.444069 0.390193
O -3.721773 -0.905396 0.882362
H -4.390600 -0.211534 0.951372
C -3.536091 1.868258 0.136944
H -3.524221 2.408809 1.088606
H -3.493611 2.603646 -0.666471
H -4.516018 1.390014 0.033071
C -0.753234 2.673317 -0.618353
H -1.577578 3.368469 -0.489597
H -0.339816 2.766925 -1.628682
H 0.069801 2.946812 0.052132
O 1.138923 0.851815 -0.818617
C 2.317479 0.128019 -0.463032
C 2.152583 -1.338968 -0.851092
H 2.038464 -1.375283 -1.938440
C 0.934261 -1.946980 -0.167971
H 1.167397 -2.216217 0.872553
H 0.657086 -2.887113 -0.654750
H 3.056953 -1.891406 -0.592704
C 3.463937 0.798706 -1.194947
H 3.525451 1.854921 -0.924689
H 4.407112 0.314063 -0.940477
H 3.300394 0.721604 -2.271747
C 2.491291 0.276184 1.052705
O 1.737045 0.872790 1.770656
O 3.589542 -0.355618 1.471943
H 3.675581 -0.213773 2.425182
H -1.504180 -2.045785 1.163971
C -2.099130 -2.490354 -0.854916
H -1.422851 -3.340771 -0.935642
H -3.083145 -2.855504 -0.561024
H -2.168945 -1.999782 -1.827913

13.9 kJ mol^{-1}
1 1
C -1.566130 -1.425614 -0.009713
C -0.198714 -0.860458 -0.000234
C -0.100097 0.610233 -0.037709
C -1.211512 1.448121 0.064117
C -2.467274 0.845070 0.089516
C -2.628912 -0.581971 0.034279
O -3.916769 -0.986777 0.041865
H -3.979566 -1.944461 -0.009952
C -3.712295 1.646224 0.175658
H -3.527025 2.714015 0.248864
H -4.303476 1.315397 1.036262
H -4.341048 1.442490 -0.698196
C -1.038739 2.936978 0.114807
H 0.013914 3.207903 0.072115
H -1.452248 3.344659 1.041096
H -1.548772 3.422094 -0.721527
O 1.078574 1.178813 -0.100291
C 2.266998 0.413227 -0.407964
C 2.743274 -0.254035 0.885136
O 2.092657 -0.331537 1.891592
O 3.960432 -0.764555 0.729635
H 4.227808 -1.181405 1.561678
C 3.275006 1.425359 -0.914009
H 2.887104 1.907395 -1.813425
H 4.214729 0.926668 -1.151629
H 3.465492 2.189794 -0.158058
C 1.892880 -0.631338 -1.452134
C 0.803479 -1.573803 -0.955308
H 0.255901 -1.976002 -1.809223
H 1.243913 -2.422846 -0.428953
H 1.554851 -0.081034 -2.335930
H 2.786757 -1.189376 -1.736113
H 0.177224 -1.041028 1.029525
C -1.733934 -2.909390 -0.016112
H -2.519752 -3.230705 0.676425
H -0.820323 -3.421918 0.286982
H -1.998816 -3.280821 -1.014325

18.7 kJ mol^{-1}
1 1
C -1.660495 -1.347434 -0.156059
C -2.669689 -0.406070 -0.102031
O -3.910376 -0.826364 0.061425
H -4.545136 -0.100661 0.061443
C -2.410256 1.049679 -0.154408
C -1.026471 1.449232 -0.495680
C -0.049369 0.498989 -0.542683
C -0.354541 -0.882978 -0.385120

C	0.759360	-1.873774	-0.457933
C	2.063983	-1.299288	-0.989818
H	2.030007	-1.211882	-2.079694
C	2.302630	0.098736	-0.432337
C	3.541329	0.756134	-1.007401
H	3.449299	0.810828	-2.093908
H	4.429025	0.177624	-0.750406
H	3.654205	1.768680	-0.614760
C	2.358955	0.051193	1.097832
O	1.511507	0.496802	1.823164
O	3.452343	-0.584105	1.520409
H	3.447397	-0.587693	2.488119
O	1.205345	0.932489	-0.800380
H	2.899577	-1.953005	-0.735090
H	0.895934	-2.269359	0.560208
H	0.440532	-2.735997	-1.051493
C	-0.704099	2.884431	-0.686693
H	-0.090853	3.017134	-1.582471
H	-0.096260	3.248346	0.150764
H	-1.598562	3.502675	-0.772338
H	-3.099982	1.504595	-0.881628
C	-2.743844	1.642653	1.250872
H	-3.769024	1.405604	1.544088
H	-2.637566	2.726799	1.226798
H	-2.058407	1.235235	1.996044
C	-1.959413	-2.807616	-0.005201
H	-2.990838	-2.963620	0.305373
H	-1.811805	-3.341968	-0.949225
H	-1.306583	-3.267238	0.741184

36.2 kJ mol^{-1}

1 1

C	-1.171646	-1.397344	-0.244146
C	0.151075	-0.980461	-0.323631
C	0.393443	0.389847	-0.394507
C	-0.584379	1.380244	-0.404839
C	-1.914279	0.956288	-0.367220
C	-2.183022	-0.416288	-0.278109
O	-3.478991	-0.766128	-0.216284
H	-3.578868	-1.721427	-0.179325
C	-3.039781	1.943266	-0.393187
H	-3.985039	1.453336	-0.618617
H	-3.146373	2.438575	0.578385
H	-2.865314	2.721623	-1.139428
C	-0.247516	2.837354	-0.467205
H	0.815039	3.025095	-0.323621
H	-0.799764	3.389490	0.296898
H	-0.535905	3.256502	-1.436715
O	1.719691	0.858291	-0.368470
C	2.729612	0.121509	-0.574247
C	1.082438	0.019719	2.658258
O	0.276950	0.293932	3.392407
C	2.532067	-1.285056	-0.965021
C	1.329614	-1.911130	-0.266441
H	1.107957	-2.859919	-0.755305
H	1.579249	-2.137434	0.777002
H	2.346546	-1.244984	-2.053506
H	3.454690	-1.850105	-0.824441
C	4.046140	0.754748	-0.441019
H	4.673525	0.490336	-1.298784
H	3.967549	1.835443	-0.333916
H	4.543479	0.334401	0.443475
C	-1.569924	-2.836568	-0.105586
H	-2.177134	-3.171319	-0.955689
H	-2.150428	-2.999453	0.810307
H	-0.713466	-3.505692	-0.046547

38.4 kJ mol^{-1}

1 1

C	0.895238	-1.394531	0.132961
C	-0.256373	-0.502963	0.314313
C	0.007872	0.932878	0.137066
C	1.264658	1.513790	0.061508
C	2.323648	0.613652	-0.018770
C	2.117492	-0.818391	-0.019091
O	3.260478	-1.520683	-0.167925
H	3.085248	-2.465552	-0.190662
C	3.723980	1.081053	-0.155125
H	4.352369	0.601459	0.601986
H	4.129540	0.749453	-1.117997
H	3.815422	2.161427	-0.078904
C	1.422691	3.001442	-0.012279
H	0.450684	3.491969	0.031413
H	1.909980	3.302188	-0.943313
H	2.026516	3.375382	0.818920
O	-1.112191	1.604355	0.034459
C	-2.208416	0.634236	0.098858
C	-1.535386	-0.584065	-0.600125
O	-2.338508	-1.690886	-0.432214
H	-2.032473	-2.385578	-1.024917
O	-1.145700	-0.399735	-1.911456
H	-1.924497	-0.255335	-2.461319
C	-2.260096	0.180562	1.555645
C	-0.943867	-0.594842	1.732707
H	-0.285727	-0.187149	2.500246
H	-1.120882	-1.648691	1.947481
H	-2.343099	1.046336	2.214125
H	-3.127529	-0.461800	1.709698
C	-3.436438	1.222702	-0.517592
H	-3.792214	2.071571	0.069725
H	-3.240440	1.573340	-1.534228
H	-4.224833	0.467252	-0.542768
C	0.717848	-2.876839	0.170389
H	1.457375	-3.363913	0.816748
H	0.819092	-3.310523	-0.833157
H	-0.264274	-3.158337	0.551221

40.5 kJ mol^{-1}

1 1

C	1.913018	-1.327100	-0.092016
C	0.549940	-1.010486	-0.057189
C	0.231721	0.332185	0.041266
C	1.128893	1.387756	0.097767
C	2.484251	1.064515	0.004406
C	2.850224	-0.286353	-0.076490
O	4.174013	-0.541784	-0.133414

H 4.337730 -1.483300 -0.230246
C 3.537937 2.129286 0.013239
H 3.269537 2.958492 -0.645280
H 3.676226 2.536992 1.020905
H 4.495745 1.727501 -0.311686
C 0.671940 2.810627 0.230728
H 0.573873 3.292660 -0.748960
H 1.394483 3.393631 0.802102
H -0.286677 2.891543 0.747006
O -1.164246 0.715225 0.143418
H -1.530563 1.355847 -0.543724
C -2.242971 -0.290368 0.301403
C -3.376559 0.466816 -0.379398
O -3.132340 1.475979 -1.004116
O -4.546339 -0.098579 -0.240735
H -5.219932 0.408771 -0.720110
C -1.869557 -1.512439 -0.523947
C -0.517674 -2.070458 -0.107843
H -0.593804 -2.575976 0.861972
H -0.239313 -2.840189 -0.830567
H -2.658696 -2.258126 -0.403121
H -1.844286 -1.230788 -1.582535
C -2.456039 -0.524164 1.777123
H -3.278141 -1.229559 1.911382
H -1.555931 -0.944040 2.229120
H -2.704431 0.406511 2.290299
C 2.407934 -2.743059 -0.146236
H 3.132990 -2.936593 0.653144
H 2.893066 -2.967919 -1.104169
H 1.611085 -3.472721 -0.013636

98.8 kJ mol^{-1}
1 1
C -1.526515 -1.411226 -0.129633
C -0.245616 -0.897906 -0.368057
C -0.058237 0.486753 -0.372628
C -1.107594 1.404307 -0.186628
C -2.394622 0.918239 0.036855
C -2.515496 -0.465970 0.055364
O -3.872686 -1.028928 0.321373
H -4.226222 -0.798192 1.195896
H -4.524161 -0.830623 -0.370684
C -3.550901 1.852293 0.259587
H -3.395235 2.457899 1.156464
H -4.514811 1.350362 0.380725
H -3.662635 2.538811 -0.582954
C -0.778839 2.862948 -0.202543
H -0.346725 3.149540 -1.165496
H -1.647085 3.491838 -0.014111
H -0.020354 3.078382 0.555199
O 1.143501 1.055290 -0.582388
C 2.315553 0.255319 -0.440531
C 2.526870 0.016685 1.057644
O 1.772791 0.366476 1.921961
O 3.661320 -0.656490 1.278014
H 3.759580 -0.769769 2.233501
C 2.117393 -1.071208 -1.164783
C 0.921407 -1.818145 -0.593557
H 0.637142 -2.630169 -1.268796
H 1.193465 -2.297425 0.356473
H 1.957920 -0.840313 -2.222611
H 3.024727 -1.672156 -1.086949
C 3.453347 1.075021 -1.018731
H 3.532011 2.035499 -0.505173
H 3.262454 1.259907 -2.077805
H 4.396697 0.538710 -0.912122
C -1.762593 -2.890301 -0.088638
H -2.778197 -3.150247 0.205672
H -1.575261 -3.338025 -1.069467
H -1.077665 -3.365656 0.618226

Methyltrolox m/z 263 (−)
Figure S4
-1 1
C 1.363840 -1.222566 -0.403124
C 0.033005 -0.802124 -0.506696
C -0.255461 0.562626 -0.426180
C 0.758227 1.517508 -0.265068
C 2.086736 1.099335 -0.187305
C 2.368060 -0.269940 -0.241972
O 3.689630 -0.692000 -0.135704
C 4.079195 -0.903480 1.203026
H 5.120089 -1.238504 1.188778
H 4.003376 0.019145 1.793182
H 3.456813 -1.669483 1.683553
C 3.208456 2.089951 -0.040798
H 3.211460 2.557424 0.951523
H 3.121300 2.896917 -0.774728
H 4.171450 1.599635 -0.190319
C 0.346153 2.952707 -0.136985
H -0.509207 3.018026 0.540653
H 0.017940 3.365580 -1.098972
H 1.154143 3.578839 0.245429
O -1.505754 1.054198 -0.540078
C -2.601438 0.153994 -0.298742
C -2.737721 -0.206802 1.231744
O -1.973360 0.375779 2.014809
O -3.630980 -1.054313 1.446957
C -2.372989 -1.107185 -1.116265
C -1.090256 -1.788527 -0.664442
H -0.807527 -2.577165 -1.371773
H -1.276845 -2.282063 0.298456
H -3.227983 -1.769813 -0.971950
H -2.307810 -0.833064 -2.178678
C -3.834498 0.907813 -0.750950
H -4.716292 0.303942 -0.530142
H -3.789457 1.123286 -1.825541
H -3.910953 1.853444 -0.206842
C 1.700336 -2.684528 -0.461959
H 1.338569 -3.136096 -1.392529
H 2.778666 -2.838308 -0.413131
H 1.226122 -3.233734 0.359440

Methyltrolox m/z 265 (+)
Figure S4

0.0 kJ mol^{-1} (ΔF_{90K})

1 1

C	-1.346805	1.236959	-0.246247
C	-0.012512	0.852699	-0.410081
C	0.261993	-0.501712	-0.518736
O	1.464643	-0.994995	-0.680372
C	2.619852	-0.151602	-0.460365
C	3.789420	-0.879312	-1.086884
H	4.707450	-0.315387	-0.920044
H	3.623766	-0.981687	-2.161155
H	3.905367	-1.873912	-0.651650
C	2.362934	1.220270	-1.070278
H	3.235332	1.854133	-0.902747
C	1.107257	1.852257	-0.484707
H	0.810098	2.706750	-1.097625
H	1.308106	2.250522	0.518040
H	2.257322	1.082888	-2.151069
C	2.761704	-0.028936	1.060577
O	1.919232	-0.362133	1.847099
O	3.923249	0.534072	1.381903
H	3.973193	0.606929	2.345796
C	-0.788537	-1.544323	-0.471530
C	-2.184092	-1.046771	-0.402783
C	-2.418377	0.283063	-0.228154
O	-3.688326	0.761857	-0.147716
C	-4.265477	0.710548	1.157241
H	-4.305310	-0.317099	1.530956
H	-3.697727	1.329454	1.860672
H	-5.276813	1.101850	1.060094
C	-3.291003	-2.033910	-0.505099
H	-3.497804	-2.493393	0.469253
H	-3.033227	-2.844702	-1.190873
H	-4.207060	-1.549101	-0.844258
H	-0.677297	-2.156713	-1.378536
C	-0.506683	-2.481365	0.738171
H	0.509228	-2.867254	0.666509
H	-0.606357	-1.928395	1.673302
H	-1.209069	-3.314704	0.729095
C	-1.677778	2.681018	-0.135032
H	-2.725434	2.841001	0.104498
H	-1.040580	3.168604	0.608684
H	-1.474938	3.176516	-1.092814

8.6 kJ mol^{-1}

1 1

C	-1.396348	1.330150	-0.095622
C	-2.407786	0.377770	-0.071859
O	-3.641347	0.808821	0.054518
C	-4.769567	-0.039810	-0.193546
H	-4.728416	-0.428475	-1.213911
H	-5.637410	0.605335	-0.081652
H	-4.824974	-0.855765	0.527375
C	-2.106198	-1.069400	-0.071563
C	-0.737612	-1.449481	-0.483349
C	0.224489	-0.487886	-0.547454
C	-0.091134	0.887224	-0.345458
C	1.014663	1.890674	-0.405444
C	2.312544	1.343826	-0.981344
H	3.148406	1.995765	-0.722782
H	2.257032	1.290209	-2.072567
C	2.572920	-0.069763	-0.474910
C	3.807816	-0.695537	-1.092564
H	4.694700	-0.117252	-0.832192
H	3.936478	-1.719552	-0.736106
H	3.696641	-0.715297	-2.178478
O	1.477790	-0.900619	-0.851504
C	2.659232	-0.073292	1.054620
O	1.839290	-0.564483	1.782322
O	3.746774	0.572850	1.477678
H	3.761631	0.540867	2.444680
H	0.680692	2.769160	-0.965344
H	1.170206	2.252939	0.622058
C	-0.404441	-2.882075	-0.678314
H	-1.287900	-3.520728	-0.638464
H	0.090258	-3.025560	-1.644126
H	0.315148	-3.210431	0.080438
H	-2.843656	-1.621793	-0.664693
C	-2.243076	-1.535178	1.423191
H	-2.132456	-2.618578	1.471880
H	-3.213327	-1.259684	1.836570
H	-1.456302	-1.071281	2.020369
C	-1.708835	2.785365	0.076999
H	-1.047079	3.242737	0.816837
H	-1.583824	3.333899	-0.862365
H	-2.735905	2.925413	0.408066

13.7 kJ mol^{-1}

1 1

C	1.482377	-1.248868	-0.487961
C	2.376406	-0.265323	-0.199002
C	2.001791	1.121637	-0.223992
C	0.680164	1.534117	-0.408654
C	-0.281813	0.545338	-0.587122
O	-1.533792	0.923728	-0.711901
C	-2.609838	0.034287	-0.319691
C	-3.879315	0.666477	-0.841356
H	-3.838437	0.722422	-1.930849
H	-4.740503	0.065790	-0.547791
H	-4.005265	1.673924	-0.440659
C	-2.364458	-1.366712	-0.865329
C	-0.992183	-1.885039	-0.464886
H	-0.795632	-2.836039	-0.961799
H	-0.941390	-2.067320	0.612153
C	0.090072	-0.869772	-0.842271
H	0.125977	-0.843405	-1.955560
H	-3.150944	-2.031145	-0.498928
H	-2.461669	-1.322208	-1.955492
C	-2.537073	-0.015489	1.210886
O	-1.523206	0.182163	1.825724
O	-3.698008	-0.363916	1.750232
H	-3.587714	-0.422474	2.710401
C	0.294202	2.979180	-0.342830
H	0.677842	3.446980	0.567220
H	0.703339	3.531081	-1.195158
H	-0.787277	3.095095	-0.357739
C	3.061892	2.141767	-0.016451

H 2.982901 2.558073 0.996549
H 4.055138 1.714024 -0.137445
H 2.932434 2.981034 -0.703417
O 3.677517 -0.573704 0.043589
C 4.006336 -0.717631 1.425870
H 3.813877 0.208194 1.978051
H 3.433454 -1.534157 1.877055
H 5.069341 -0.949595 1.464893
C 1.875684 -2.680897 -0.550902
H 1.524991 -3.144618 -1.478461
H 1.425950 -3.245463 0.274369
H 2.958018 -2.784795 -0.496395

31.4 kJ mol^{-1}
1 1
C 1.441894 -1.207816 -0.193788
C 0.074447 -0.756167 -0.545821
C -0.230169 0.555790 -0.421385
C 0.762094 1.537538 -0.077947
C 2.109421 1.205149 0.064903
C 2.459264 -0.136005 -0.099806
O 3.734161 -0.429489 -0.061758
C 4.237863 -1.713494 -0.455034
H 3.968333 -1.917779 -1.493830
H 5.318001 -1.630564 -0.365079
H 3.873804 -2.503978 0.201326
C 3.158890 2.238697 0.348237
H 4.148040 1.788034 0.391160
H 3.172067 3.011242 -0.424818
H 2.970643 2.728396 1.307598
C 0.278445 2.924989 0.105076
H -0.480566 2.925614 0.896259
H -0.243821 3.262576 -0.796212
H 1.070325 3.625061 0.355345
O -1.473680 1.060804 -0.597669
C -2.557693 0.143762 -0.480599
C -3.783917 0.901080 -0.958265
H -3.616481 1.244368 -1.980894
H -3.975348 1.768876 -0.324237
H -4.676278 0.269123 -0.965345
C -2.259428 -1.093351 -1.332147
H -3.092898 -1.799382 -1.301355
C -0.973706 -1.767339 -0.867672
H -0.607259 -2.458243 -1.635044
H -1.167534 -2.383092 0.021833
H -2.164681 -0.754642 -2.368049
C -2.664337 -0.249003 1.008170
O -1.834944 0.039789 1.820010
O -3.726674 -0.989227 1.344077
H -4.346469 -1.078002 0.612589
H 1.773706 -1.982374 -0.893937
C 1.362018 -1.845845 1.237918
H 0.752323 -2.748702 1.192722
H 2.348527 -2.111854 1.617993
H 0.894137 -1.137594 1.923649

37.7 kJ mol^{-1}
1 1
C -0.846519 -1.145526 0.370563
C 0.430376 -0.417978 0.395276
C 0.359927 1.016032 0.071879
C -0.802741 1.766066 0.036349
C -1.982297 1.030618 0.156377
C -1.988478 -0.412904 0.257465
O -3.197916 -1.030658 0.316270
C -3.746868 -1.391628 -0.951654
H -3.918444 -0.507929 -1.575051
H -4.697648 -1.880191 -0.745135
H -3.083155 -2.083684 -1.479511
C -3.288327 1.738652 0.139433
H -3.231316 2.675500 0.697353
H -4.084363 1.113577 0.540091
H -3.550808 2.007657 -0.892580
C -0.771277 3.247911 -0.169116
H -1.133101 3.776150 0.718563
H -1.403089 3.544651 -1.010102
H 0.244322 3.588170 -0.369976
O 1.553760 1.497427 -0.170439
C 2.503107 0.388274 -0.040478
C 1.624439 -0.791951 -0.551769
O 2.281095 -1.971116 -0.280938
H 1.798074 -2.699408 -0.685665
O 1.182387 -0.709614 -1.858071
H 1.935460 -0.788066 -2.455071
C 2.577809 0.103287 1.458326
C 1.180473 -0.451557 1.785481
H 0.632159 0.127606 2.528620
H 1.224501 -1.490490 2.111681
H 3.356444 -0.634007 1.654906
H 2.815571 1.019250 2.001035
C 3.761251 0.713846 -0.778541
H 4.268764 1.563391 -0.317263
H 4.431989 -0.147495 -0.745213
H 3.552962 0.968167 -1.820943
C -0.863274 -2.625517 0.515504
H -0.125838 -2.970357 1.244509
H -1.850522 -2.973482 0.818400
H -0.622171 -3.103075 -0.443781

41.8 kJ mol^{-1}
1 1
C 1.365868 1.232879 -0.433243
C 0.039838 0.814770 -0.592053
C -0.246128 -0.552171 -0.506546
O -1.549715 -1.029580 -0.652109
C -2.588608 -0.134294 -0.353247
C -3.895786 -0.830372 -0.704791
H -4.751778 -0.190500 -0.488594
H -3.996637 -1.772099 -0.161656
H -3.872795 -1.049814 -1.772869
C -2.400424 1.183242 -1.118621
H -2.456790 0.894160 -2.171956
C -1.057355 1.824613 -0.803384
H -1.136068 2.468431 0.084210
H -0.784171 2.501408 -1.617911
H -3.231686 1.863285 -0.918767

C -2.545697 0.140768 1.135872
O -1.534351 -0.089907 1.866684
O -3.590100 0.628929 1.691116
H -3.462600 0.788079 2.642830
C 0.737056 -1.527047 -0.297457
C 2.064918 -1.101620 -0.163487
C 2.354941 0.267766 -0.215275
O 3.651429 0.676657 -0.105776
C 4.100303 0.859653 1.231235
C 3.166481 -2.103314 0.000119
H 3.066625 -2.913305 -0.726749
H 4.138539 -1.634496 -0.145086
H 3.148867 -2.562180 0.994985
C 0.387370 -2.984108 -0.272061
H -0.749702 -0.370830 1.305224
C 1.721334 2.685453 -0.518044
H 1.244562 3.265849 0.279383
H 2.798766 2.821915 -0.448136
H 1.394120 3.115917 -1.469749
H 0.712781 -3.470403 -1.198202
H -0.686187 -3.141731 -0.181837
H 0.890281 -3.496189 0.551617
H 5.144093 1.165244 1.169508
H 4.026744 -0.070500 1.805348
H 3.520268 1.641665 1.734999

41.9 kJ mol^{-1}
1 1
C -1.668508 -1.229408 -0.265157
C -0.299860 -0.954013 -0.143328
C 0.053836 0.373110 0.007146
C -0.805258 1.459069 0.031456
C -2.165894 1.174143 -0.134410
C -2.573637 -0.162723 -0.251552
O -3.898356 -0.433959 -0.419071
C -4.634361 -0.546624 0.795076
H -4.253570 -1.371832 1.407126
H -5.666881 -0.748026 0.512786
H -4.588330 0.381949 1.373843
C -3.174881 2.280088 -0.198392
H -2.838100 3.081673 -0.860021
H -3.349981 2.723480 0.788189
H -4.125033 1.905922 -0.576923
C -0.305235 2.863816 0.199419
H -0.113759 3.340619 -0.769152
H 0.613986 2.908368 0.787576
H -1.044206 3.476674 0.715656
O 1.454746 0.700415 0.193187
C 2.498202 -0.346780 0.340572
C 2.109697 -1.535065 -0.526818
C 0.725939 -2.050969 -0.166098
H 0.745030 -2.569794 0.799828
H 0.437318 -2.805855 -0.900997
H 2.127731 -1.229474 -1.578843
H 2.869859 -2.309039 -0.398092
C 2.672261 -0.630036 1.813062
H 1.752993 -1.042951 2.231482
H 2.929991 0.278444 2.360363
H 3.474837 -1.358366 1.943330
C 3.669186 0.398386 -0.288156
O 3.470996 1.445029 -0.866916
O 4.815594 -0.213928 -0.159421
H 5.514850 0.290893 -0.603472
H 1.873686 1.362984 -0.445105
C -2.148950 -2.638260 -0.432794
H -1.868498 -3.263417 0.420888
H -3.231324 -2.663308 -0.541717
H -1.719659 -3.097802 -1.329133

77.3 kJ mol^{-1}
1 1
C 1.351140 -1.217849 -0.391701
C 2.288396 -0.212422 -0.233445
C 2.049470 1.153432 -0.203939
C 0.713132 1.545728 -0.313862
C -0.276929 0.557513 -0.439133
C 0.019105 -0.807797 -0.526221
C -1.069514 -1.818163 -0.754480
C -2.367941 -1.150399 -1.172528
H -3.203088 -1.843581 -1.066883
H -2.322868 -0.828064 -2.217424
C -2.631965 0.076680 -0.325364
C -3.890160 0.815645 -0.741216
H -4.760543 0.175082 -0.587324
H -4.024144 1.728747 -0.156689
H -3.826292 1.082994 -1.798140
O -1.552041 1.022794 -0.477236
C -2.741367 -0.297338 1.165986
O -3.172280 -1.349109 1.535846
O -2.354971 0.655240 2.020961
H -2.030872 1.413850 1.517361
H -0.757607 -2.532180 -1.522580
H -1.236710 -2.398501 0.160738
C 0.334531 2.994614 -0.277375
H 0.370682 3.385716 0.746265
H 1.019919 3.598901 -0.875421
H -0.673493 3.145492 -0.659073
C 3.139672 2.172260 -0.060191
H 2.984986 2.785688 0.832198
H 4.132242 1.726896 -0.002652
H 3.140199 2.851930 -0.916797
O 3.686928 -0.609576 -0.053899
H 3.933603 -1.309253 -0.677636
C 4.122685 -0.907033 1.326328
H 3.518287 -1.724226 1.715932
H 3.959114 0.013855 1.878984
H 5.181215 -1.153330 1.265709
C 1.695465 -2.677664 -0.456118
H 1.062770 -3.253252 0.224138
H 1.527918 -3.074928 -1.462208
H 2.729285 -2.909879 -0.184322

Trolox *m/z* 205 (+)

Figure S6

0.0 kJ mol^{-1} (ΔF_{90K})

1 1
C -1.076699 1.397065 -0.062488
C -2.096810 0.430739 0.008546
C -1.843498 -0.949972 0.029158
C -0.523556 -1.405729 -0.023334
C 0.464041 -0.425868 -0.062197
C 0.234566 0.946598 -0.080766
C 1.414977 1.869921 -0.166756
C 2.627468 1.222512 0.494828
C 2.806964 -0.173973 0.058174
O 1.787777 -0.897363 -0.159780
C 4.116578 -0.811216 -0.120037
H 4.618151 -0.349038 -0.980782
H 4.749229 -0.597969 0.748625
H 4.028244 -1.884329 -0.281575
H 3.552575 1.783737 0.354065
H 2.458836 1.155915 1.584832
H 1.634876 2.108550 -1.215061
H 1.203681 2.816960 0.331220
C -0.190295 -2.867418 -0.022818
H -0.694917 -3.379189 -0.846186
H 0.878474 -3.043679 -0.128394
H -0.516046 -3.339871 0.908439
C -3.010681 -1.889479 0.103845
H -2.699741 -2.931319 0.142507
H -3.665494 -1.787058 -0.770319
H -3.612349 -1.708088 1.002840
O -3.353046 0.903896 0.053163
H -3.990098 0.184034 0.081922
C -1.410490 2.856746 -0.110415
H -2.467789 3.005243 -0.321536
H -0.834011 3.373552 -0.881489
H -1.196872 3.340718 0.849396

24.7 kJ mol^{-1}
1 1
C -0.879744 1.456051 0.034824
C -1.961146 0.626297 0.088928
C -1.837816 -0.805054 0.001402
C -0.601488 -1.424779 -0.153339
C 0.531222 -0.604536 -0.186395
O 1.700511 -1.192539 -0.300785
C 2.917568 -0.433720 -0.056477
C 2.823676 0.903347 -0.763450
C 1.520291 1.553178 -0.513302
C 0.412556 0.843190 -0.167913
H 1.444229 2.624341 -0.675158
H 2.944353 0.797724 -1.853281
H 3.643155 1.554135 -0.441999
C 3.152238 -0.337726 1.435505
H 2.394245 0.271821 1.934526
H 3.150563 -1.333709 1.881380
H 4.130177 0.113982 1.620208
H 3.686998 -1.052692 -0.517459
C -0.459217 -2.914324 -0.246693
H -1.052646 -3.311509 -1.073980
H 0.578436 -3.196615 -0.410858
H -0.800034 -3.400425 0.671808
C -3.096083 -1.585542 0.092857
H -3.755223 -1.316436 -0.740735
H -2.929410 -2.659045 0.085841
H -3.646040 -1.303932 0.996466
O -3.230377 1.051620 0.231240
H -3.276602 2.011684 0.244146
C -1.030320 2.940459 0.146213
H -0.125302 3.418430 0.522186
H -1.279539 3.400921 -0.817103
H -1.818850 3.207629 0.857410

45.3 kJ mol^{-1}
1 1
C -0.596613 1.387594 -0.060857
C -1.932711 1.070152 0.032896
O -2.910110 1.998829 0.112334
H -2.542346 2.881445 0.199263
C -2.390980 -0.277099 0.031473
C -1.474953 -1.327523 -0.009856
C -0.114349 -1.031546 -0.065880
O 0.787530 -2.012921 -0.117891
H 0.348117 -2.870807 -0.091059
C 0.368667 0.317552 -0.067173
C 1.726473 0.631670 -0.022893
C 2.876751 -0.186530 0.003920
C 4.100938 0.405545 0.069956
C 5.386652 -0.308155 0.104301
H 6.014506 0.015198 -0.735725
H 5.944109 -0.027483 1.006994
H 5.270795 -1.391565 0.072939
H 4.145548 1.494906 0.099301
H 2.798236 -1.263534 -0.029434
H 1.951075 1.693849 0.018170
C -1.897694 -2.767838 0.002399
H -1.382305 -3.332784 0.789444
H -1.701894 -3.256960 -0.959479
H -2.960810 -2.877542 0.203884
C -3.858828 -0.537072 0.073494
H -4.429589 0.379063 -0.052491
H -4.136213 -0.978034 1.038329
H -4.150690 -1.248323 -0.703502
C -0.184560 2.827870 -0.139712
H 0.701185 2.974888 -0.758021
H -0.959026 3.436500 -0.615474
H 0.017180 3.256202 0.848896

109.1 kJ mol^{-1}
1 1
C 1.074029 1.411821 -0.032707
C 2.095685 0.449683 0.046212
C 1.860451 -0.929576 0.023337
C 0.540132 -1.402761 -0.084780
C -0.433485 -0.431422 -0.155768
C -0.238899 0.951218 -0.145283
C -1.404352 1.821102 -0.252874
C -2.634783 1.370545 0.019901
C -2.820802 -0.038484 0.462631
O -1.812948 -0.873411 -0.325353

H -2.058337 -0.847344 -1.268406
C -4.171885 -0.645862 0.225737
H -4.895124 -0.105795 0.842862
H -4.197315 -1.696834 0.516831
H -4.489165 -0.538017 -0.815968
H -2.459678 -0.211311 1.481899
H -3.513322 2.001217 -0.055769
H -1.262820 2.846581 -0.571185
C 0.222660 -2.868027 -0.114144
H -0.850418 -3.054461 -0.129374
H 0.659891 -3.346321 -0.995191
H 0.629916 -3.368620 0.767841
C 3.031908 -1.859767 0.122523
H 3.569698 -1.726427 1.069261
H 3.741910 -1.692367 -0.696641
H 2.737240 -2.905828 0.070990
O 3.340508 0.948738 0.153360
H 3.992520 0.244586 0.210881
C 1.454006 2.855965 0.022379
H 2.099364 3.114062 -0.822134
H 2.034723 3.056692 0.926799
H 0.592444 3.520953 0.020254

130.8 kJ mol^{-1}

1 1
C -1.143534 -1.361727 -0.019891
C -2.129192 -0.366281 0.045217
C -1.833097 1.006251 0.025425
C -0.494528 1.392505 -0.064114
C 0.439819 0.374908 -0.126494
C 0.199064 -0.978820 -0.101814
C 1.327363 -1.971276 -0.135422
C 2.666474 -1.356216 0.094171
C 2.936918 -0.067820 0.063288
O 1.822750 0.818054 -0.276886
H 1.926268 1.709503 0.102515
C 4.179714 0.708292 0.240631
H 4.105482 1.411730 1.078738
H 4.996819 0.021105 0.461103
H 4.437582 1.264095 -0.666601
H 3.504814 -2.013115 0.299744
H 1.341707 -2.504526 -1.095688
H 1.168122 -2.738759 0.628424
C -0.056962 2.828751 -0.090689
H 0.342078 3.118027 -1.069100
H -0.885919 3.497850 0.131348
H 0.701870 3.050928 0.676757
C -2.938471 2.016481 0.094318
H -3.909367 1.527795 0.051746
H -2.880314 2.725509 -0.735923
H -2.894332 2.587252 1.027519
O -3.434283 -0.691011 0.135873
H -3.552731 -1.644431 0.138826
C -1.564497 -2.801515 -0.005203
H -2.028483 -3.076333 0.949742
H -0.730738 -3.483651 -0.162489
H -2.285012 -3.010527 -0.805101

142.4 kJ mol^{-1}

1 1
C -0.010963 1.128456 -0.459142
C -0.369006 -0.168596 -0.656781
C 0.604490 -1.175818 -0.345736
O 0.192748 -2.398560 -0.495369
H 0.878336 -3.046243 -0.270966
C 1.957030 -0.945911 0.106011
C 2.355910 0.336633 0.290818
C 1.376352 1.443964 0.010886
O 1.717498 2.591957 0.160174
C 3.706775 0.733807 0.756867
H 3.795699 1.817703 0.800709
H 3.906037 0.325800 1.753840
H 4.479150 0.341938 0.087045
C 2.826439 -2.139937 0.355735
H 3.822637 -1.840854 0.675177
H 2.956876 -2.746073 -0.549455
H 2.422733 -2.777740 1.152427
C -1.747804 -0.582472 -1.098807
H -1.694288 -1.538755 -1.625988
H -2.129085 0.153950 -1.810206
C -2.678220 -0.683988 0.079995
C -3.792555 0.033242 0.212325
C -4.746437 -0.068123 1.350974
H -5.736833 -0.372402 0.997017
H -4.875222 0.902937 1.840313
H -4.411724 -0.790765 2.098150
H -4.055164 0.736631 -0.579415
H -2.409969 -1.406155 0.850333
C -0.930016 2.264753 -0.684021
H -1.850073 2.113787 -0.108035
H -1.226020 2.313795 -1.738702
H -0.467022 3.209209 -0.405428

Trolox *m/z* 251 → 205 (+)

Figure S7

0.0 kJ mol^{-1} (ΔG)

1 1
C 1.493231 1.342582 -0.244171
C 0.212272 0.815642 -0.434099
C 0.033465 -0.540504 -0.193002
O -1.122147 -1.153277 -0.259791
C -2.340229 -0.378882 -0.337842
C -3.418257 -1.342287 -0.784090
H -4.379951 -0.829936 -0.819254
H -3.495183 -2.183878 -0.092861
H -3.180695 -1.723068 -1.779470
C -2.128813 0.782166 -1.299681
H -1.935975 0.354614 -2.288827
C -0.963356 1.656302 -0.856008
H -1.267323 2.307613 -0.027558
H -0.680047 2.313079 -1.682258
H -3.048922 1.365026 -1.366840
C -2.583912 0.150990 1.079810
O -1.766795 0.127534 1.957298
O -3.802116 0.675450 1.184924

H -3.916321 1.008137 2.086723
C 1.151050 -1.453203 0.128064
C 2.466427 -0.810551 0.347929
C 2.602272 0.531126 0.148970
O 3.834273 1.064156 0.314616
C 3.623263 -1.648033 0.753589
H 3.304682 -2.576247 1.230869
H 4.235519 -1.907682 -0.119544
H 4.274942 -1.093192 1.431455
C 1.251752 -2.555017 -0.961741
H 0.863322 -1.954655 1.065493
C 1.754008 2.798161 -0.426754
H 0.864663 3.363082 -0.692201
H 2.147571 3.232255 0.501856
H 2.505862 2.957986 -1.209385
H 1.540494 -2.113222 -1.917685
H 0.288385 -3.052715 -1.069653
H 2.000657 -3.292944 -0.675636
H 3.813555 2.023892 0.322560

57.6 kJ mol^{-1}
1 1
C 1.502006 1.413496 -0.167586
C 0.218841 1.051087 -0.576786
C -0.047158 -0.302610 -0.793672
O -1.303247 -0.719361 -1.100275
C -2.296724 -0.052735 -0.322990
C -3.620899 -0.740447 -0.614954
H -4.435846 -0.241259 -0.089602
H -3.591720 -1.791386 -0.319344
H -3.800637 -0.688092 -1.690204
C -2.302519 1.419964 -0.722181
H -2.699742 1.436945 -1.739638
C -0.903081 2.054799 -0.679392
H -0.835183 2.726663 0.180911
H -0.768457 2.685674 -1.563669
H -3.001370 1.976595 -0.094529
C -1.948365 -0.281750 1.147565
O -0.978385 -0.928952 1.553216
O -2.803759 0.245934 1.978927
H -2.548827 0.041814 2.892838
C 0.872162 -1.327345 -0.426741
C 2.177825 -0.937303 -0.008413
C 2.461603 0.417409 0.114032
O 3.701334 0.744808 0.530509
C 3.217433 -1.967498 0.277773
H 2.903081 -2.620666 1.098520
H 3.376196 -2.607691 -0.595784
H 4.164341 -1.507115 0.549902
C 0.586628 -2.767847 -0.769327
H 0.014697 -1.079475 0.652790
C 1.894860 2.846024 0.021718
H 1.121180 3.538692 -0.302151
H 2.118404 3.065046 1.072986
H 2.788985 3.083611 -0.567756
H 1.121927 -3.042345 -1.683007
H -0.475918 -2.928268 -0.942844
H 0.920338 -3.436938 0.025257

H 3.825329 1.697291 0.542027

31.0 kJ mol^{-1}
1 1
C 1.560779 1.392509 -0.127469
C 0.275239 0.931019 -0.424160
C 0.056590 -0.452260 -0.459530
O -1.199696 -0.983417 -0.764988
C -2.310242 -0.180522 -0.465054
C -3.544412 -0.911391 -0.973596
H -4.450819 -0.340506 -0.770052
H -3.627229 -1.901591 -0.521244
H -3.432028 -1.033885 -2.051457
C -2.147324 1.206258 -1.101316
H -2.116451 1.006919 -2.176248
C -0.868503 1.892073 -0.641086
H -1.052210 2.454459 0.284703
H -0.588622 2.641539 -1.386029
H -3.026760 1.824405 -0.906488
C -2.387831 -0.041613 1.042472
O -1.412335 -0.266846 1.820333
O -3.502797 0.316002 1.560321
H -3.450775 0.392360 2.529263
C 1.074413 -1.390608 -0.237560
C 2.363202 -0.922547 0.031284
C 2.584348 0.458982 0.081626
O 3.843804 0.858125 0.362003
C 3.494695 -1.877790 0.254270
H 3.381534 -2.405895 1.207442
H 3.528358 -2.635383 -0.533066
H 4.449407 -1.356340 0.273682
C 0.800178 -2.860782 -0.328992
H -0.572352 -0.439851 1.285977
C 1.888819 2.854039 -0.028932
H 1.012168 3.490629 -0.134041
H 2.337732 3.097678 0.941476
H 2.594304 3.160301 -0.811460
H 1.235291 -3.271651 -1.246430
H -0.267139 -3.074017 -0.349783
H 1.253793 -3.398047 0.507231
H 3.920278 1.813753 0.303443

68.4 kJ mol^{-1}
1 1
C -1.050790 -1.398694 -0.233102
C 0.117420 -0.647230 -0.562381
C 0.027263 0.771873 -0.524001
O 1.214967 1.435116 -0.481139
C 2.248251 0.499853 -0.172231
C 3.463959 1.247289 0.315999
H 4.231524 0.543099 0.643079
H 3.216220 1.914810 1.143815
H 3.871725 1.844183 -0.502328
C 2.460641 -0.416544 -1.360825
H 2.645324 0.211123 -2.234639
C 1.172825 -1.238676 -1.490953
H 1.350413 -2.281829 -1.229805
H 0.784111 -1.222940 -2.511950

H 3.336998 -1.048622 -1.209056
C 1.578778 -0.364694 0.930986
O 1.072098 0.186084 2.017936
O 2.140382 -1.527014 1.160189
H 1.898187 -1.851427 2.040001
C -1.150168 1.475684 -0.295768
C -2.290749 0.715451 -0.028072
C -2.213704 -0.703695 0.033977
O -3.380468 -1.308991 0.340068
C -3.613013 1.342514 0.247794
H -3.963573 1.071970 1.248933
H -3.587572 2.426084 0.159062
H -4.362010 0.946011 -0.444908
C -1.153005 2.976136 -0.290775
H 0.692213 1.054358 1.823814
C -1.035798 -2.896340 -0.229870
H -0.032249 -3.290616 -0.066556
H -1.659827 -3.305377 0.572250
H -1.396708 -3.312722 -1.178066
H -1.801216 3.362822 -1.082095
H -0.151548 3.369115 -0.461892
H -1.522532 3.375731 0.657666
H -3.296767 -2.264896 0.291577

37.0 kJ mol^{-1}

1 1
C -0.899200 -1.391668 -0.107391
C 0.246510 -0.501626 -0.298895
C -0.014363 0.932529 -0.147969
O 1.115725 1.605283 -0.036079
C 2.212689 0.632995 -0.119516
C 3.455892 1.226363 0.461113
H 4.251078 0.477589 0.447587
H 3.304560 1.552561 1.492599
H 3.784376 2.083677 -0.129908
C 2.240931 0.158286 -1.567912
H 2.312021 1.013506 -2.241448
C 0.929509 -0.628308 -1.719826
H 1.105675 -1.687627 -1.905797
H 0.264787 -0.240231 -2.491771
H 3.109503 -0.482999 -1.721628
C 1.548596 -0.580750 0.609443
O 1.336265 -0.427574 1.963458
O 2.222453 -1.759275 0.416215
H 2.612147 -2.015303 1.259788
C -1.265719 1.513648 -0.060569
C -2.330443 0.611934 0.029077
C -2.126630 -0.814364 0.031859
O -3.267764 -1.522682 0.163120
C -3.729879 1.082754 0.161483
H -4.154622 0.719831 1.104246
H -3.817426 2.165160 0.116773
H -4.346701 0.630331 -0.622239
C -1.426599 3.001865 0.002486
H 1.183571 0.493605 2.196305
C -0.727230 -2.873060 -0.153552
H 0.325048 -3.152131 -0.168868
H -1.174973 -3.346556 0.728937
H -1.205088 -3.306419 -1.041709
H -2.034532 3.366762 -0.829758
H -0.456035 3.494567 -0.051341
H -1.912476 3.311536 0.931447
H -3.088729 -2.467463 0.154090

Figure S8

0.0 kJ mol^{-1} (ΔG)

1 1
C 1.560778 1.392510 -0.127471
C 0.275238 0.931018 -0.424161
C 0.056591 -0.452260 -0.459529
O -1.199695 -0.983420 -0.764984
C -2.310241 -0.180525 -0.465053
C -3.544412 -0.911396 -0.973593
H -4.450819 -0.340512 -0.770047
H -3.627226 -1.901596 -0.521240
H -3.432029 -1.033890 -2.051453
C -2.147324 1.206253 -1.101319
H -2.116451 1.006913 -2.176251
C -0.868505 1.892070 -0.641089
H -1.052214 2.454457 0.284699
H -0.588624 2.641536 -1.386032
H -3.026761 1.824399 -0.906492
C -2.387829 -0.041611 1.042471
O -1.412337 -0.266850 1.820334
O -3.502793 0.316015 1.560319
H -3.450771 0.392377 2.529261
C 1.074414 -1.390608 -0.237557
C 2.363202 -0.922546 0.031285
C 2.584347 0.458983 0.081626
O 3.843804 0.858127 0.362002
C 3.494697 -1.877787 0.254270
H 3.381532 -2.405902 1.207436
H 3.528366 -2.635373 -0.533073
H 4.449407 -1.356335 0.273691
C 0.800178 -2.860781 -0.328990
H -0.572354 -0.439859 1.285980
C 1.888817 2.854040 -0.028936
H 1.012164 3.490629 -0.134044
H 2.337729 3.097681 0.941472
H 2.594300 3.160302 -0.811465
H 1.235255 -3.271642 -1.246450
H -0.267140 -3.074019 -0.349743
H 1.253825 -3.398053 0.507210
H 3.920275 1.813756 0.303454

185.4 kJ mol^{-1}

1 1
C 1.429830 1.419166 -0.180823
C 0.152347 0.988465 -0.559809
C -0.065045 -0.384897 -0.705254
O -1.335474 -0.862870 -0.989669
C -2.339272 -0.110823 -0.349751
C -3.668287 -0.837369 -0.517736
H -4.474863 -0.290361 -0.026981
H -3.615511 -1.852613 -0.120086

H -3.874396 -0.894275 -1.588355
C -2.347475 1.306492 -0.914861
H -2.615818 1.180740 -1.966509
C -0.976284 1.971811 -0.765781
H -0.997708 2.663801 0.081841
H -0.778037 2.586556 -1.649117
H -3.131282 1.904295 -0.444455
C -1.999788 -0.090097 1.119406
O -1.087859 -0.957979 1.681328
O -2.423705 0.559179 2.065455
H -1.466314 -0.239121 2.606230
C 0.918222 -1.350174 -0.436856
C 2.196501 -0.908954 -0.051268
C 2.426001 0.466092 0.074432
O 3.665109 0.837152 0.461438
C 3.300311 -1.888408 0.193760
H 3.078071 -2.530253 1.052895
H 3.432209 -2.545845 -0.670600
H 4.241488 -1.379123 0.388526
C 0.649527 -2.810863 -0.664987
H -0.220056 -1.065983 1.139433
C 1.776362 2.871687 -0.037566
H 0.957401 3.529179 -0.321951
H 2.050633 3.122835 0.994320
H 2.622170 3.138674 -0.683235
H 1.148491 -3.142719 -1.581430
H -0.414464 -3.011643 -0.782044
H 1.040990 -3.424395 0.149863
H 3.754729 1.793627 0.455799

71.5 kJ mol^{-1}
1 1
C 1.448698 1.427585 -0.146096
C 0.170650 0.982085 -0.496779
C -0.036986 -0.396424 -0.617028
O -1.299781 -0.907154 -0.883217
C -2.346322 -0.126489 -0.383022
C -3.651312 -0.864906 -0.645214
H -4.500376 -0.306363 -0.248140
H -3.630905 -1.864154 -0.206325
H -3.763034 -0.962613 -1.726778
C -2.296799 1.265264 -1.008315
H -2.423612 1.089771 -2.079888
C -0.966278 1.953211 -0.713090
H -1.068689 2.587213 0.174862
H -0.724098 2.631222 -1.536706
H -3.139979 1.871169 -0.670539
C -2.209568 0.024739 1.131778
O -1.168013 -1.047414 1.688475
O -2.720395 0.672445 1.943221
H -0.971620 -0.860563 2.621585
C 0.968552 -1.347844 -0.385260
C 2.250761 -0.890040 -0.040059
C 2.464879 0.486678 0.079090
O 3.709901 0.876474 0.428541
C 3.376231 -1.854527 0.167151
H 3.196971 -2.493820 1.038234
H 3.484856 -2.515256 -0.697804
H 4.317282 -1.331447 0.322407
C 0.713211 -2.812201 -0.603572
H -0.321618 -1.069097 1.118140
C 1.778657 2.884449 -0.005974
H 0.923146 3.529300 -0.196413
H 2.138410 3.120238 1.002865
H 2.557861 3.183380 -0.718193
H 1.181493 -3.136264 -1.538922
H -0.350701 -3.032185 -0.679514
H 1.146885 -3.418563 0.195049
H 3.787778 1.833732 0.408271

19.1 kJ mol^{-1} (separated products)
1 1
C -0.881524 -1.400281 -0.029441
C 0.322874 -0.596050 -0.203327
C 0.180703 0.854666 -0.079768
O 1.356472 1.445733 0.037571
C 2.389677 0.420000 0.048822
C 3.630978 0.926975 0.704588
H 4.350405 0.110226 0.793844
H 3.414599 1.306541 1.704549
H 4.081626 1.723713 0.109384
C 2.456281 -0.127819 -1.381067
H 2.619633 0.691438 -2.083096
C 1.094958 -0.826724 -1.576178
H 1.195558 -1.902588 -1.725969
H 0.500738 -0.415132 -2.391922
H 3.289323 -0.828348 -1.466459
C 1.603190 -0.743882 0.702653
O 1.897476 -1.549652 1.509320
C -1.028093 1.526457 -0.020023
C -2.159151 0.710039 0.043621
C -2.066307 -0.730731 0.059886
O -3.261900 -1.342491 0.165566
C -3.520994 1.287246 0.145654
H -3.990947 0.964017 1.081478
H -3.523182 2.372947 0.096076
H -4.155570 0.880926 -0.648904
C -1.074417 3.022405 0.040739
C -0.784881 -2.889095 -0.027308
H 0.215992 -3.220941 0.252847
H -1.473010 -3.334994 0.699000
H -1.018546 -3.314725 -1.011514
H -1.637092 3.432475 -0.802087
H -0.068371 3.439803 0.008191
H -1.551829 3.365799 0.962050
H -3.165478 -2.299113 0.149771

0 1
H 0.759872 -0.467510 -0.000000
O 0.000000 0.116878 0.000000
H -0.759872 -0.467511 0.000000

85.2 kJ mol^{-1}
1 1
C -1.257203 -1.375459 -0.142760
C 0.026688 -0.892431 -0.409037

C 0.213358 0.501025 -0.435092
O 1.482452 1.036199 -0.580887
C 2.495076 0.229122 -0.299210
C 3.793265 0.941664 -0.124492
H 4.518575 0.310510 0.391651
H 3.662632 1.883588 0.408390
H 4.192974 1.157450 -1.122133
C 2.469536 -1.100885 -0.985710
H 2.585728 -0.848894 -2.048332
C 1.156230 -1.838887 -0.745306
H 1.278372 -2.571004 0.059756
H 0.895482 -2.414222 -1.637575
H 3.338107 -1.697123 -0.701188
C 1.881564 -0.252226 1.425158
O 1.978688 -0.275215 2.545155
C -0.819195 1.438611 -0.270808
C -2.092336 0.946707 -0.018107
C -2.284646 -0.448854 0.050676
O -3.543779 -0.839154 0.304537
C -3.280358 1.829049 0.193139
H -3.740822 1.626269 1.164135
H -3.024208 2.885163 0.143487
H -4.046742 1.621698 -0.559619
C -0.543531 2.910240 -0.357554
C -1.580240 -2.839944 -0.085518
H -0.717840 -3.469122 -0.298099
H -1.945326 -3.132166 0.906680
H -2.348581 -3.108676 -0.820584
H -1.146068 3.365843 -1.147787
H 0.502239 3.116126 -0.575560
H -0.801556 3.411541 0.579441
H -3.611107 -1.798006 0.334626

7.5 kJ mol^{-1}

1 1
C -1.171670 -1.397357 -0.244086
C 0.151050 -0.980480 -0.323603
C 0.393423 0.389825 -0.394523
O 1.719672 0.858264 -0.368520
C 2.729588 0.121473 -0.574290
C 4.046118 0.754712 -0.441096
H 4.543479 0.334370 0.443387
H 3.967530 1.835407 -0.334001
H 4.673484 0.490293 -1.298875
C 2.532030 -1.285101 -0.965026
H 2.346488 -1.245058 -2.053508
C 1.329588 -1.911151 -0.266406
H 1.579240 -2.137425 0.777040
H 1.107921 -2.859954 -0.755238
H 3.454654 -1.850151 -0.824447
C 1.082579 0.019841 2.658218
O 0.277152 0.294127 3.392406
C -0.584397 1.380224 -0.404868
C -1.914298 0.956274 -0.367216
C -2.183045 -0.416299 -0.278064
O -3.479013 -0.766134 -0.216215
C -3.039794 1.943258 -0.393193
H -3.146322 2.438647 0.578345
H -2.865363 2.721555 -1.139506
H -3.985069 1.453319 -0.618530
C -0.247532 2.837331 -0.467285
C -1.569950 -2.836576 -0.105480
H -0.713493 -3.505697 -0.046404
H -2.150467 -2.999429 0.810411
H -2.177148 -3.171358 -0.955580
H -0.535952 3.256452 -1.436797
H 0.815028 3.025074 -0.323741
H -0.799755 3.389491 0.296820
H -3.578892 -1.721432 -0.179234

−4.7 kJ mol^{-1} (separated products)

1 1
C 1.098853 -1.378983 -0.052552
C -0.225255 -0.960742 -0.073960
C -0.470595 0.410324 -0.061957
O -1.797207 0.871146 -0.168484
C -2.812520 0.145617 0.055773
C -4.126180 0.772420 -0.128460
H -4.624783 0.300163 -0.985441
H -4.044129 1.844677 -0.298917
H -4.757597 0.562896 0.742016
C -2.619419 -1.244783 0.503816
H -2.442580 -1.168334 1.591754
C -1.406028 -1.887082 -0.162134
H -1.629108 -2.122636 -1.210229
H -1.204801 -2.834721 0.337130
H -3.539451 -1.816467 0.372805
C 0.503436 1.403145 -0.022271
C 1.832575 0.979669 0.041006
C 2.105932 -0.394639 0.013640
O 3.403084 -0.743488 0.047392
C 2.955160 1.967953 0.110188
H 3.135804 2.422003 -0.870578
H 2.727351 2.776572 0.808143
H 3.879698 1.488332 0.425928
C 0.162617 2.860655 -0.025677
C 1.506106 -2.820845 -0.115906
H 0.655575 -3.495315 -0.196340
H 2.135225 -3.015154 -0.992692
H 2.066992 -3.121637 0.777555
H 0.377994 3.307784 0.950322
H -0.886343 3.041505 -0.253807
H 0.769355 3.392013 -0.762446
H 3.505692 -1.699201 0.044500

0 1
C 0.000000 -0.000000 -0.643420
O -0.000000 0.000000 0.482565

2.6 kJ mol^{-1}

1 1
C 0.895188 -1.394555 0.132997
C -0.256403 -0.502910 0.314305
C 0.007919 0.932920 0.136835
C 1.264753 1.513749 0.061335
C 2.323696 0.613573 -0.018790

C 2.117447 -0.818501 -0.019133
O 3.260407 -1.520806 -0.168092
H 3.085180 -2.465685 -0.190593
C 3.724063 1.080854 -0.154956
H 4.352254 0.601222 0.602321
H 4.129854 0.749082 -1.117663
H 3.815601 2.161218 -0.078766
C 1.422938 3.001403 -0.012430
H 0.451019 3.492053 0.031843
H 1.909716 3.302137 -0.943737
H 2.027280 3.375160 0.818464
O -1.112082 1.604420 0.034168
C -2.208375 0.634352 0.098796
C -1.535453 -0.584120 -0.599963
O -2.338656 -1.690856 -0.431729
H -2.032786 -2.385646 -1.024404
O -1.145904 -0.400114 -1.911400
H -1.924716 -0.255305 -2.461131
C -2.259974 0.180938 1.555667
C -0.943709 -0.594434 1.732781
H -0.285500 -0.186552 2.500164
H -1.120717 -1.648221 1.947879
H -2.342954 1.046832 2.213989
H -3.127390 -0.461413 1.709876
C -3.436436 1.222700 -0.517688
H -3.792866 2.070995 0.070057
H -3.240226 1.574083 -1.534024
H -4.224449 0.466867 -0.543641
C 0.717580 -2.876844 0.170402
H 1.457456 -3.364149 0.816184
H 0.818028 -3.310405 -0.833276
H -0.264320 -3.158168 0.551933

170.9 kJ mol^{-1}

1 1
C 0.911965 -1.387151 0.152660
C -0.235049 -0.505986 0.359884
C -0.003000 0.927005 0.192435
C 1.238957 1.521629 0.069967
C 2.313814 0.633092 -0.025026
C 2.130951 -0.796426 -0.008488
O 3.280729 -1.487723 -0.151257
H 3.120806 -2.434302 -0.096798
C 3.704063 1.121500 -0.192407
H 4.342432 0.691334 0.586467
H 4.116135 0.750603 -1.137609
H 3.776395 2.205671 -0.166243
C 1.378466 3.010250 -0.024643
H 0.403092 3.491710 0.042285
H 1.838387 3.307273 -0.970774
H 2.000624 3.399154 0.785875
O -1.146250 1.583450 0.093491
C -2.225088 0.605736 0.163331
C -1.554484 -0.625343 -0.503184
O -2.168834 -1.743884 -0.676757
H -1.822577 -1.366132 -1.929998
O -1.205346 -0.393229 -2.030846
H -1.694257 0.337795 -2.438277
C -2.268048 0.102618 1.606086
C -0.946552 -0.670382 1.762635
H -0.300398 -0.291493 2.554382
H -1.115133 -1.736305 1.918552
H -2.358809 0.947229 2.290700
H -3.131976 -0.551331 1.733879
C -3.478544 1.167959 -0.427037
H -3.888787 1.952243 0.212111
H -3.305129 1.603102 -1.415579
H -4.218997 0.369882 -0.515757
C 0.723294 -2.865477 0.207772
H 0.961501 -3.265464 1.201898
H 1.360854 -3.382022 -0.518013
H -0.311036 -3.132016 -0.018072

16.8 kJ mol^{-1}

1 1
C -0.929874 -1.443957 -0.080918
C 0.267739 -0.657625 -0.355871
C 0.129441 0.796423 -0.333509
C -1.080216 1.472081 -0.308860
C -2.205399 0.667362 -0.148262
C -2.110260 -0.766956 -0.010438
O -3.303925 -1.365943 0.182713
H -3.205416 -2.321098 0.228714
C -3.566758 1.251957 -0.063789
H -4.222001 0.775712 -0.800134
H -4.010331 1.018567 0.910617
H -3.571803 2.328532 -0.214113
C -1.124307 2.968046 -0.375873
H -0.118310 3.378225 -0.462527
H -1.586806 3.391948 0.519442
H -1.700703 3.307826 -1.240517
O 1.303091 1.391646 -0.308126
C 2.334982 0.371144 -0.253422
C 1.591026 -0.770033 0.479915
O 1.966057 -1.601848 1.227742
H 0.762163 0.050778 3.205283
O 0.574761 0.667584 2.492610
H 0.435742 1.515063 2.921917
C 2.351921 -0.270896 -1.648901
C 0.980435 -0.974168 -1.746935
H 0.354884 -0.609770 -2.561659
H 1.073206 -2.057559 -1.833738
H 2.491739 0.499581 -2.408689
H 3.178307 -0.981053 -1.715788
C 3.596227 0.919441 0.324770
H 4.025935 1.677304 -0.333435
H 3.404159 1.358634 1.304728
H 4.319728 0.109996 0.442568
C -0.827631 -2.928198 0.033477
H -1.030677 -3.426383 -0.923057
H -1.532555 -3.327643 0.770217
H 0.168917 -3.227902 0.362347

19.0 kJ mol^{-1} (separated products)

1 1
C 0.881525 -1.400281 0.029439

C -0.322873 -0.596050 0.203326
C -0.180702 0.854665 0.079771
C 1.028093 1.526457 0.020025
C 2.159152 0.710041 -0.043620
C 2.066309 -0.730730 -0.059884
O 3.261903 -1.342489 -0.165561
H 3.165478 -2.299111 -0.149797
C 3.520995 1.287245 -0.145654
H 4.155579 0.880901 0.648886
H 3.990938 0.964029 -1.081489
H 3.523191 2.372945 -0.096054
C 1.074415 3.022406 -0.040740
H 0.068369 3.439803 -0.008192
H 1.551825 3.365796 -0.962054
H 1.637091 3.432479 0.802083
O -1.356471 1.445734 -0.037568
C -2.389676 0.420005 -0.048819
C -1.603193 -0.743880 -0.702652
O -1.897484 -1.549653 -1.509312
C -2.456282 -0.127819 1.381068
C -1.094960 -0.826733 1.576174
H -0.500742 -0.415152 2.391924
H -1.195568 -1.902598 1.725953
H -2.619624 0.691436 2.083101
H -3.289327 -0.828344 1.466463
C -3.630976 0.926978 -0.704592
H -4.081626 1.723720 -0.109395
H -3.414592 1.306539 -1.704554
H -4.350403 0.110229 -0.793848
C 0.784883 -2.889095 0.027301
H 1.018526 -3.314727 1.011511
H 1.473025 -3.334994 -0.698993
H -0.215985 -3.220939 -0.252877

0 1
H 0.759872 -0.467510 -0.000000
O 0.000000 0.116878 0.000000
H -0.759872 -0.467511 0.000000

85.7 kJ mol^{-1}
1 1
C 1.255916 -1.380078 0.142126
C -0.026090 -0.894977 0.409507
C -0.210065 0.499107 0.433477
C 0.826554 1.428253 0.266899
C 2.102258 0.935550 0.023313
C 2.291312 -0.459329 -0.053957
O 3.542487 -0.869495 -0.317407
H 3.595611 -1.829398 -0.333630
C 3.261071 1.859771 -0.179668
H 4.207403 1.327381 -0.108214
H 3.216212 2.326411 -1.170276
H 3.255110 2.664525 0.558829
C 0.572974 2.901967 0.343226
H -0.488335 3.133762 0.405249
H 0.990297 3.412144 -0.528665
H 1.060171 3.326986 1.226729
O -1.476186 1.040262 0.580857
C -2.491860 0.235493 0.301528
C -1.890277 -0.247636 -1.422066
O -1.994994 -0.273127 -2.541247
C -2.468358 -1.093792 0.990209
C -1.158880 -1.837475 0.745991
H -0.899522 -2.415642 1.636815
H -1.286012 -2.567609 -0.060159
H -2.579383 -0.839492 2.052810
H -3.340248 -1.687337 0.710183
C -3.788885 0.951740 0.131384
H -4.182790 1.170590 1.130635
H -3.658037 1.892359 -0.403760
H -4.518683 0.321986 -0.380102
C 1.571061 -2.846354 0.082215
H 2.345276 -3.119171 0.809764
H 1.924217 -3.141096 -0.913566
H 0.707865 -3.470975 0.304628

7.5 kJ mol^{-1}
1 1
C -1.171646 -1.397344 -0.244146
C 0.151075 -0.980461 -0.323631
C 0.393443 0.389847 -0.394507
C -0.584379 1.380244 -0.404839
C -1.914279 0.956288 -0.367220
C -2.183022 -0.416288 -0.278109
O -3.478991 -0.766128 -0.216284
H -3.578868 -1.721427 -0.179325
C -3.039781 1.943266 -0.393187
H -3.985039 1.453336 -0.618617
H -3.146373 2.438575 0.578385
H -2.865314 2.721623 -1.139428
C -0.247516 2.837354 -0.467205
H 0.815039 3.025095 -0.323621
H -0.799764 3.389490 0.296898
H -0.535905 3.256502 -1.436715
O 1.719691 0.858291 -0.368470
C 2.729612 0.121509 -0.574247
C 1.082438 0.019719 2.658258
O 0.276950 0.293932 3.392407
C 2.532067 -1.285056 -0.965021
C 1.329614 -1.911130 -0.266441
H 1.107957 -2.859919 -0.755305
H 1.579249 -2.137434 0.777002
H 2.346546 -1.244984 -2.053506
H 3.454690 -1.850105 -0.824441
C 4.046140 0.754748 -0.441019
H 4.673525 0.490336 -1.298784
H 3.967549 1.835443 -0.333916
H 4.543479 0.334401 0.443475
C -1.569924 -2.836568 -0.105586
H -2.177134 -3.171319 -0.955689
H -2.150428 -2.999453 0.810307
H -0.713466 -3.505692 -0.046547

−4.8 kJ mol^{-1} (separated products)
1 1
C 1.098853 -1.378983 -0.052552

C -0.225255 -0.960742 -0.073960
C -0.470595 0.410324 -0.061957
C 0.503436 1.403145 -0.022271
C 1.832575 0.979669 0.041006
C 2.105932 -0.394639 0.013640
O 3.403084 -0.743488 0.047392
H 3.505692 -1.699201 0.044500
C 2.955160 1.967953 0.110188
H 3.879698 1.488332 0.425928
H 3.135804 2.422003 -0.870578
H 2.727351 2.776572 0.808143
C 0.162617 2.860655 -0.025677
H -0.886343 3.041505 -0.253807
H 0.769355 3.392013 -0.762446
H 0.377994 3.307784 0.950322
O -1.797207 0.871146 -0.168484
C -2.812520 0.145617 0.055773
C -2.619419 -1.244782 0.503816
C -1.406028 -1.887082 -0.162134
H -1.204801 -2.834721 0.337130
H -1.629108 -2.122636 -1.210229
H -2.442580 -1.168334 1.591754
H -3.539451 -1.816467 0.372805
C -4.126180 0.772420 -0.128460
H -4.757597 0.562896 0.742016
H -4.044129 1.844677 -0.298917
H -4.624783 0.300163 -0.985441
C 1.506106 -2.820845 -0.115906
H 2.066992 -3.121637 0.777555
H 2.135225 -3.015154 -0.992692
H 0.655575 -3.495315 -0.196340

0 1
C 0.000000 -0.000000 -0.643420
O -0.000000 0.000000 0.482565

Trolox *m/z* 205 (−)

Figure S11

45.9 kJ mol⁻¹ (Δ*F*₉₀K)

-1 1
C 2.276922 0.276051 0.258113
C 1.388136 1.316594 -0.009719
C 0.084864 1.058518 -0.438191
C -0.336144 -0.262770 -0.608162
C 0.528798 -1.377685 -0.326211
C 1.863531 -1.045958 0.090847
C 2.798249 -2.186610 0.358995
H 2.308122 -3.106273 0.029999
H 3.033627 -2.294337 1.428688
H 3.756219 -2.075393 -0.166514
O 0.126984 -2.582096 -0.444819
C -1.725590 -0.625850 -1.067704
C -2.697570 -0.643318 0.067427
C -3.835467 0.047826 0.144555
C -4.771244 0.030198 1.312089
H -5.774466 -0.313115 1.027164
H -4.400344 -0.635395 2.097110
H -4.893342 1.029495 1.750625
H -4.118514 0.685160 -0.696002
H -2.400076 -1.277040 0.904045
H -2.093003 0.028054 -1.868868
H -1.638281 -1.647655 -1.460329
C -0.827115 2.229280 -0.697006
H -1.831669 1.905407 -0.965414
H -0.451782 2.871039 -1.506023
H -0.945732 2.864034 0.195633
O 1.839864 2.631259 0.145699
H 1.065118 3.188214 0.234236
C 3.672882 0.585018 0.724350
H 3.781298 1.644942 0.959623
H 3.929585 0.000255 1.615180
H 4.416940 0.331531 -0.042482

0.0 kJ mol⁻¹

-1 1
C 0.805721 1.458086 -0.017402
C 2.048596 0.743784 0.089896
C 1.946714 -0.691545 0.007253
C 0.724861 -1.342469 -0.156045
C -0.451133 -0.595204 -0.265562
C -0.411793 0.798860 -0.203720
C -1.690018 1.595654 -0.290386
C -2.876336 0.739419 -0.713245
C -2.810759 -0.626338 -0.041685
O -1.645303 -1.300035 -0.468082
C -2.883898 -0.557085 1.479042
H -3.794506 -0.039351 1.800297
H -2.016481 -0.027503 1.881088
H -2.887483 -1.566687 1.898982
H -3.636008 -1.249292 -0.408437
H -2.848288 0.570246 -1.795699
H -3.827740 1.234143 -0.481527
H -1.895831 2.063408 0.684387
H -1.565246 2.431157 -0.989080
C 0.669100 -2.843949 -0.224884
H 1.153308 -3.220881 -1.135637
H -0.362906 -3.197534 -0.221163
H 1.199275 -3.297961 0.620664
C 3.223431 -1.470714 0.111096
H 3.257947 -2.102303 1.011340
H 4.047566 -0.754907 0.161802
H 3.378266 -2.138092 -0.748251
O 3.163999 1.337113 0.251162
C 0.866013 2.952853 0.081522
H 1.905467 3.227746 0.277766
H 0.545899 3.452523 -0.845454
H 0.234208 3.350557 0.888997

37.8 kJ mol⁻¹

-1 1
C 0.704316 1.428761 -0.000153
C 2.075134 1.146032 0.000155
O 3.002758 2.007563 -0.000069
C 2.470473 -0.297169 0.000192
C 1.576108 -1.317445 0.000256

C	0.139378	-1.036001	0.000338
O	-0.678221	-1.962225	-0.000870
C	-0.287610	0.395354	-0.000036
C	-1.631761	0.676042	-0.000169
H	-1.903663	1.729922	-0.000389
C	-2.788725	-0.268067	0.000009
C	-4.128729	0.459657	0.000368
C	-5.319213	-0.488526	0.000491
H	-5.301342	-1.138633	0.882169
H	-5.301700	-1.138414	-0.881356
H	-6.273214	0.049987	0.000752
H	-4.181863	1.118567	-0.876483
H	-4.181494	1.118337	0.877413
H	-2.733191	-0.949068	-0.861876
H	-2.732767	-0.949236	0.861719
C	1.999242	-2.755886	0.000104
H	1.115353	-3.395949	0.001122
H	2.608927	-2.997641	0.879914
H	2.607017	-2.998040	-0.880938
C	3.940690	-0.573014	0.000103
H	4.472760	0.379791	-0.000224
H	4.231479	-1.159743	0.880371
H	4.231260	-1.160305	-0.879857
C	0.285125	2.862935	-0.000502
H	-0.321227	3.123190	0.881794
H	1.182120	3.485767	-0.000695
H	-0.321327	3.122720	-0.882863

46.6 kJ mol^{-1}

-1 1

C	-2.255584	-0.424201	0.214839
C	-2.029917	0.998185	0.196522
C	-0.704625	1.416519	-0.186087
C	0.277526	0.487652	-0.530036
C	0.012521	-0.884629	-0.469248
C	-1.252896	-1.342084	-0.100926
C	-1.531518	-2.818620	-0.049523
H	-2.279963	-3.110228	-0.797814
H	-1.936535	-3.111884	0.926636
H	-0.622092	-3.391239	-0.237751
O	1.000462	-1.810695	-0.820592
H	1.808569	-1.549410	-0.367352
C	1.668514	0.922992	-0.934564
C	2.651540	0.654084	0.163703
C	3.728548	-0.130233	0.073451
C	4.669270	-0.427926	1.197371
H	4.725675	-1.504083	1.401452
H	5.689435	-0.096778	0.966450
H	4.351909	0.071769	2.116880
H	3.957035	-0.589059	-0.890818
H	2.410302	1.107907	1.126364
H	1.985630	0.396420	-1.843334
H	1.663215	1.993209	-1.161320
C	-0.449001	2.896560	-0.204052
H	0.404925	3.192716	0.420914
H	-1.349785	3.382966	0.179467
H	-0.260050	3.284823	-1.215806
O	-2.942041	1.833664	0.497906
C	-3.626932	-0.890341	0.601880
H	-3.624268	-1.471684	1.535888
H	-4.252400	-0.006350	0.747595
H	-4.083643	-1.530598	-0.165940

200.1 kJ mol^{-1}

-1 1

C	-1.084740	-1.347463	-0.056638
C	0.250572	-0.921168	-0.041957
C	0.524770	0.460680	-0.026241
O	1.762599	0.946714	-0.054454
C	2.850578	0.027675	-0.452798
C	4.097865	0.782475	-0.099765
H	4.179513	1.724063	-0.655125
H	4.976623	0.177630	-0.351996
H	4.168500	1.037680	0.990858
C	2.691371	-1.188013	0.404394
C	1.388501	-1.904684	0.068964
H	1.154597	-2.650486	0.847298
H	1.512037	-2.446966	-0.876553
H	3.541626	-1.862452	0.251857
H	2.679268	-0.930095	1.498645
C	-0.532070	1.392590	0.005361
C	-1.857740	0.957412	0.028149
C	-2.116442	-0.412224	-0.016959
O	-3.444517	-0.817512	-0.029335
C	-2.993149	1.939196	0.090314
H	-3.099278	2.494261	-0.850839
H	-3.937049	1.427839	0.282538
H	-2.832895	2.681113	0.880160
C	-0.203266	2.855977	0.028249
H	-0.873814	3.430917	-0.619036
H	0.826045	3.004972	-0.298980
H	-0.290361	3.274002	1.040883
C	-1.435194	-2.810395	-0.118950
H	-2.161080	-3.018138	-0.917675
H	-1.862065	-3.182340	0.824653
H	-0.556074	-3.418599	-0.329461
H	-3.456589	-1.769361	0.080345

Figure S11

2,3-*trans* 6.7 kJ mol^{-1} (ΔF_{90K})

-1 1

C	2.276922	0.276051	0.258113
C	1.388136	1.316594	-0.009719
C	0.084864	1.058518	-0.438191
C	-0.336144	-0.262770	-0.608162
C	0.528798	-1.377685	-0.326211
C	1.863531	-1.045958	0.090847
C	2.798249	-2.186610	0.358995
H	2.308122	-3.106273	0.029999
H	3.033627	-2.294337	1.428688
H	3.756219	-2.075393	-0.166514
O	0.126984	-2.582096	-0.444819
C	-1.725590	-0.625850	-1.067704
C	-2.697570	-0.643318	0.067427
C	-3.835467	0.047826	0.144555

C -4.771244 0.030198 1.312089
H -5.774466 -0.313115 1.027164
H -4.400344 -0.635395 2.097110
H -4.893342 1.029495 1.750625
H -4.118514 0.685160 -0.696002
H -2.400076 -1.277040 0.904045
H -2.093003 0.028054 -1.868868
H -1.638281 -1.647655 -1.460329
C -0.827115 2.229280 -0.697006
H -1.831669 1.905407 -0.965414
H -0.451782 2.871039 -1.506023
H -0.945732 2.864034 0.195633
O 1.839864 2.631259 0.145699
H 1.065118 3.188214 0.234236
C 3.672882 0.585018 0.724350
H 3.781298 1.644942 0.959623
H 3.929585 0.000255 1.615180
H 4.416940 0.331531 -0.042482

2,3-*cis* 9.4 kJ mol^{-1}

-1 1
C 2.090069 -0.146545 0.193124
C 1.536902 1.131444 0.118338
C 0.223738 1.321200 -0.316492
C -0.562734 0.216676 -0.649303
C -0.044681 -1.124646 -0.593990
C 1.321455 -1.252960 -0.170006
C 1.892941 -2.637867 -0.113087
H 2.826497 -2.728855 -0.685090
H 1.150557 -3.325252 -0.525226
H 2.120190 -2.955254 0.915573
O -0.758855 -2.139140 -0.900318
C -1.997568 0.360998 -1.098266
C -2.950335 0.834820 -0.030287
C -3.393566 0.111217 1.001239
C -3.020926 -1.305010 1.298535
H -3.917743 -1.929937 1.406450
H -2.373124 -1.739821 0.527683
H -2.481684 -1.364148 2.253141
H -4.070549 0.597395 1.706313
H -3.289267 1.869893 -0.095174
H -2.069308 1.038018 -1.960918
H -2.287810 -0.637497 -1.443556
C -0.313484 2.725257 -0.401773
H -0.648837 3.104002 0.575585
H -1.177202 2.780634 -1.067174
H 0.444654 3.417933 -0.785349
O 2.337136 2.228914 0.464916
H 1.775511 2.860910 0.915563
C 3.507041 -0.327529 0.662130
H 3.565105 -1.069755 1.467052
H 4.152534 -0.693298 -0.147161
H 3.921115 0.614509 1.024533

3,4-*trans* 0.0 kJ mol^{-1}

-1 1
C 2.444233 0.232954 0.001647
C 1.525398 1.292085 0.014342
C 0.154795 1.065519 -0.007782
C -0.334287 -0.259331 0.013579
C 0.576654 -1.390422 -0.030251
C 1.985357 -1.075425 -0.047317
C 2.940700 -2.229082 -0.094344
H 3.508553 -2.339108 0.841482
H 3.677311 -2.128259 -0.902486
H 2.359305 -3.141491 -0.245421
O 0.167756 -2.589063 -0.040309
C -1.734343 -0.630480 0.039836
C -2.834927 0.020909 0.465826
H -2.769693 1.013700 0.907248
C -4.210736 -0.567195 0.404308
C -5.154788 0.199969 -0.523603
H -6.164199 -0.230013 -0.526269
H -5.238750 1.248456 -0.215772
H -4.773008 0.189838 -1.549274
H -4.659283 -0.592129 1.409999
H -4.138273 -1.609374 0.072198
H -1.871184 -1.661583 -0.292575
C -0.763568 2.251746 -0.132670
H -0.280720 3.063515 -0.688967
H -1.671577 1.975439 -0.672678
H -1.085330 2.657025 0.838295
O 2.042840 2.592790 0.004671
H 1.414150 3.152414 0.461341
C 3.919451 0.522346 0.024782
H 4.427719 -0.087402 0.780281
H 4.108900 1.575416 0.236482
H 4.387674 0.282187 -0.938904

3,4-*cis* 5.7 kJ mol^{-1}

-1 1
C 2.111712 -0.654940 0.079549
C 2.026411 0.741672 0.091011
C 0.806819 1.384356 -0.078005
C -0.369866 0.636876 -0.287894
C -0.337743 -0.808406 -0.315707
C 0.960853 -1.409442 -0.110733
C 1.023338 -2.907037 -0.131738
H 1.446311 -3.319868 0.794640
H 1.641700 -3.286769 -0.958064
H 0.006109 -3.283920 -0.259344
O -1.364520 -1.522531 -0.525409
C -1.609084 1.357654 -0.597120
C -2.856660 1.141591 -0.153095
C -3.296689 0.144326 0.867582
C -4.267962 -0.883191 0.291874
H -5.139323 -0.397394 -0.166304
H -4.633502 -1.565336 1.068842
H -3.734556 -1.464023 -0.463678
H -2.424664 -0.378063 1.263995
H -3.780732 0.683218 1.698656
H -3.648788 1.772171 -0.565285
H -1.501564 2.185131 -1.303314
C 0.763979 2.889486 -0.002196
H -0.236572 3.237906 0.259582
H 1.448836 3.269450 0.766063

H 1.033429 3.372243 -0.954839
O 3.201308 1.467736 0.316399
H 3.091525 2.322600 -0.100425
C 3.441385 -1.327925 0.278618
H 3.645481 -2.044806 -0.525460
H 4.250625 -0.597100 0.307187
H 3.462422 -1.897208 1.216953

1,2 17.2 kJ mol^{-1}
-1 1
C 2.363618 -0.025597 0.157754
C 1.697752 1.190859 0.020304
C 0.331912 1.238861 -0.272890
C -0.378555 0.046915 -0.439966
C 0.252274 -1.237228 -0.282330
C 1.659694 -1.220348 -0.002050
C 2.346477 -2.545812 0.131261
H 1.635187 -3.322432 -0.160755
H 2.665975 -2.751552 1.164063
H 3.243681 -2.617838 -0.498149
O -0.416388 -2.319252 -0.388745
C -1.843133 -0.011814 -0.754209
C -2.691343 -0.263731 0.505297
C -4.131453 -0.472053 0.193308
C -5.145563 0.281274 0.618571
H -6.173144 0.075015 0.332346
H -4.976583 1.133053 1.274063
H -4.346899 -1.313549 -0.467519
H -2.276456 -1.169417 0.964513
H -2.571122 0.565045 1.213653
H -2.206632 0.885268 -1.268112
H -1.992214 -0.867397 -1.423818
C -0.319379 2.593868 -0.382507
H -0.187943 3.189065 0.535489
H -1.395270 2.516428 -0.537949
H 0.092183 3.184266 -1.212694
O 2.436981 2.369360 0.167873
H 1.814602 3.075058 0.348411
C 3.832741 -0.043944 0.478360
H 4.190463 0.954703 0.733806
H 4.044889 -0.717190 1.316895
H 4.424281 -0.406903 -0.372685

Trolox m/z 249 → 205 (−)

Figure S13

2,3-*trans* 0.0 kJ mol^{-1} (ΔG)
-1 1
C -1.084740 -1.347463 -0.056638
C 0.250572 -0.921168 -0.041957
C 0.524770 0.460680 -0.026241
O 1.762599 0.946714 -0.054454
C 2.850578 0.027675 -0.452798
C 4.097865 0.782475 -0.099765
H 4.179513 1.724063 -0.655125
H 4.976623 0.177630 -0.351996
H 4.168500 1.037680 0.990858
C 2.691371 -1.188013 0.404394
C 1.388501 -1.904684 0.068964
H 1.154597 -2.650486 0.847298
H 1.512037 -2.446966 -0.876553
H 3.541626 -1.862452 0.251857
H 2.679268 -0.930095 1.498645
C -0.532070 1.392590 0.005361
C -1.857740 0.957412 0.028149
C -2.116442 -0.412224 -0.016959
O -3.444517 -0.817512 -0.029335
C -2.993149 1.939196 0.090314
H -3.099278 2.494261 -0.850839
H -3.937049 1.427839 0.282538
H -2.832895 2.681113 0.880160
C -0.203266 2.855977 0.028249
H -0.873814 3.430917 -0.619036
H 0.826045 3.004972 -0.298980
H -0.290361 3.274002 1.040883
C -1.435194 -2.810395 -0.118950
H -2.161080 -3.018138 -0.917675
H -1.862065 -3.182340 0.824653
H -0.556074 -3.418599 -0.329461
H -3.456589 -1.769361 0.080345

2,3-*trans* 188.6 kJ mol^{-1}
-1 1
C -1.082266 -1.347721 -0.036848
C 0.250876 -0.917377 -0.034011
C 0.518494 0.466859 -0.042128
O 1.753104 0.980238 -0.084624
C 2.887665 0.037860 -0.338583
C 4.103868 0.795991 0.088804
H 4.179816 1.768490 -0.411372
H 5.003851 0.217254 -0.142244
H 4.098278 0.984439 1.184352
C 2.743273 -1.284283 0.317052
C 1.401767 -1.900895 -0.022406
H 1.188535 -2.695481 0.704860
H 1.427055 -2.392759 -1.011827
H 3.254634 -1.074152 -0.891575
H 2.991739 -1.354067 1.402228
C -0.545256 1.391609 -0.025855
C -1.869968 0.950504 0.022857
C -2.121304 -0.418703 0.010803
O -3.445866 -0.832681 0.047404
C -3.008302 1.928921 0.082712
H -3.122313 2.473987 -0.863351
H -3.949008 1.416542 0.287109
H -2.845526 2.680016 0.863481
C -0.232477 2.859313 -0.037698
H -0.904571 3.410001 -0.704543
H 0.797268 3.013944 -0.360286
H -0.334506 3.302250 0.962940
C -1.423242 -2.814314 -0.081795
H -2.214449 -3.023953 -0.815098
H -1.760154 -3.198385 0.892600
H -0.560618 -3.410811 -0.376550
H -3.446700 -1.784347 0.159610

2,3-*trans* −152.2 kJ mol^{-1}
-1 1
C -0.468088 -1.321375 -0.334665
C 0.378268 -0.272124 -0.699128
C -0.023353 1.106352 -0.574782
O 0.758877 2.062969 -0.885164
C 3.539413 0.392111 0.338464
C 4.445161 0.315305 1.525107
H 4.145428 1.034297 2.298313
H 5.484474 0.552056 1.262118
H 4.429570 -0.684159 1.972260
C 2.762533 -0.601389 -0.090471
C 1.778603 -0.480771 -1.216534
H 1.844500 -1.359211 -1.871852
H 2.023857 0.409047 -1.804426
H 3.462173 1.350581 -0.173496
H 2.774829 -1.550093 0.453661
C -1.357562 1.327990 -0.088575
C -2.192327 0.274255 0.286103
C -1.744217 -1.039640 0.159121
O -2.610959 -2.078955 0.520136
C -3.569280 0.548198 0.824943
H -4.231944 0.949461 0.046650
H -4.024138 -0.363360 1.215843
H -3.538018 1.296350 1.625483
C -1.814912 2.751090 0.022908
H -2.786685 2.917904 -0.461041
H -1.060041 3.383641 -0.450739
H -1.920612 3.077891 1.068543
C -0.051436 -2.765964 -0.434559
H -0.764787 -3.353173 -1.027448
H 0.020793 -3.246209 0.554677
H 0.929634 -2.873901 -0.897862
H -2.069196 -2.817453 0.801951

1,2 0.0 kJ mol^{-1}
-1 1
C 1.084739 -1.347463 -0.056635
C -0.250573 -0.921167 -0.041948
C -0.524770 0.460681 -0.026231
C 0.532071 1.392590 0.005368
C 1.857741 0.957411 0.028151
C 2.116441 -0.412224 -0.016962
O 3.444515 -0.817513 -0.029348
H 3.456588 -1.769364 0.080311
C 2.993153 1.939192 0.090311
H 3.937040 1.427840 0.282610
H 2.832865 2.681156 0.880106
H 3.099333 2.494200 -0.850870
C 0.203267 2.855977 0.028252
H -0.826047 3.004970 -0.298966
H 0.290373 3.274009 1.040884
H 0.873808 3.430914 -0.619044
O -1.762599 0.946715 -0.054439
C -2.850574 0.027678 -0.452802
C -2.691376 -1.188013 0.404390
C -1.388502 -1.904683 0.068972
H -1.154602 -2.650481 0.847312
H -1.512030 -2.446972 -0.876543
H -2.679285 -0.930097 1.498641
H -3.541631 -1.862450 0.251843
C -4.097865 0.782476 -0.099782
H -4.168504 1.037689 0.990836
H -4.976618 0.177622 -0.352015
H -4.179515 1.724059 -0.655151
C 1.435194 -2.810394 -0.118947
H 2.161052 -3.018145 -0.917696
H 1.862102 -3.182328 0.824644
H 0.556069 -3.418603 -0.329420

1,2 180.1 kJ mol^{-1}
-1 1
C 1.055724 -1.356535 -0.045648
C -0.265320 -0.890270 0.008572
C -0.504016 0.496947 0.034549
C 0.581159 1.397104 0.029037
C 1.895010 0.924988 0.017465
C 2.114262 -0.450369 -0.030622
O 3.428741 -0.892426 -0.074050
H 3.418651 -1.843945 0.038573
C 3.056912 1.876382 0.045890
H 3.992999 1.342265 0.212590
H 2.936499 2.623781 0.837534
H 3.148775 2.427255 -0.899039
C 0.297729 2.869924 0.054846
H -0.753882 3.042377 -0.175108
H 0.496850 3.302314 1.045112
H 0.920021 3.412915 -0.665101
O -1.732631 1.016249 0.065799
C -2.894447 0.124472 -0.365393
C -2.734978 -1.135033 0.427324
C -1.427673 -1.847369 0.092176
H -1.216309 -2.622816 0.846247
H -1.546346 -2.366191 -0.868851
H -2.752757 -0.902455 1.514015
H -3.583286 -1.800032 0.235298
C -4.214043 0.760554 -0.206019
H -4.700624 0.801128 0.792303
H -3.855490 -0.045306 -1.215010
H -4.364630 1.661383 -0.801688
C 1.365184 -2.828028 -0.125015
H 2.074113 -3.048399 -0.935307
H 1.795214 -3.219103 0.809307
H 0.468302 -3.412366 -0.327829

1,2 −149.6 kJ mol^{-1}
-1 1
C -0.001808 -1.093616 0.507053
C -0.470270 0.212532 0.677060
C 0.346005 1.355031 0.358689
C 1.676232 1.074591 -0.104103
C 2.149179 -0.231774 -0.233113
C 1.310093 -1.302872 0.071717
O 1.803261 -2.603014 -0.100773
H 1.374109 -3.159691 0.549794
C 3.552414 -0.487101 -0.709244

H	3.798526	-1.548287	-0.651092
H	4.280697	0.074452	-0.112067
H	3.685887	-0.162950	-1.749600
C	2.547877	2.247511	-0.437090
H	1.955473	3.154978	-0.297184
H	3.435554	2.307632	0.209570
H	2.914027	2.216810	-1.472962
O	-0.101577	2.542838	0.486427
C	-3.045703	-0.044089	-0.973853
C	-2.787465	1.010884	0.043447
C	-1.847966	0.544260	1.172226
H	-1.746961	1.390844	1.861103
H	-2.307923	-0.285966	1.719658
H	-2.277050	1.864515	-0.421450
H	-3.738630	1.371516	0.457320
C	-4.243999	-0.484720	-1.357978
H	-5.159480	-0.073692	-0.936480
H	-2.151607	-0.484190	-1.416487
H	-4.352902	-1.264885	-2.106199
C	-0.859908	-2.304155	0.776507
H	-0.740733	-3.053567	-0.015435
H	-0.611135	-2.793969	1.731550
H	-1.919406	-2.051032	0.816818

2,3-*cis* 21.2 kJ mol^{-1}

-1 1

C	0.906375	1.395473	0.050224
C	-0.379078	0.834868	-0.003241
C	-0.515286	-0.570350	-0.052739
C	0.635706	-1.388835	-0.064622
C	1.908348	-0.823850	0.007655
C	2.026477	0.565531	0.053190
O	3.307597	1.100205	0.104174
H	3.220472	2.045421	0.236554
C	3.139420	-1.685408	0.036907
H	4.020678	-1.093166	0.285917
H	3.321617	-2.162835	-0.934749
H	3.042583	-2.490068	0.773524
C	0.452186	-2.875816	-0.140848
H	0.482824	-3.338937	0.855494
H	-0.526816	-3.099007	-0.567502
H	1.231231	-3.350788	-0.745422
O	-1.688214	-1.204130	-0.110502
C	-2.976558	-0.508634	-0.375729
C	-2.680092	0.874778	-0.887636
C	-1.639574	1.641563	-0.040036
H	-1.461432	2.646338	-0.445020
H	-2.045250	1.777693	0.974655
H	-2.306124	0.844137	-1.921798
H	-3.625674	1.430811	-0.915428
C	-3.691779	-0.477132	0.951882
H	-4.691507	-0.045448	0.816892
H	-3.190203	0.108948	1.770199
H	-3.829821	-1.490565	1.345041
C	1.124021	2.882901	0.135573
H	1.765305	3.153477	0.988343
H	1.591307	3.289828	-0.772222
H	0.184751	3.415021	0.282665

2,3-*cis* 213.9 kJ mol^{-1}

-1 1

C	0.961956	-1.378567	-0.041408
C	-0.342009	-0.883182	0.095172
C	-0.530243	0.506818	0.158148
C	0.573468	1.382303	0.110896
C	1.870996	0.881430	-0.003190
C	2.044591	-0.500811	-0.077061
O	3.341382	-0.982257	-0.196857
H	3.297324	-1.938603	-0.155379
C	3.070290	1.788268	-0.055413
H	3.986443	1.206199	-0.156487
H	3.155510	2.396072	0.852995
H	3.014405	2.481572	-0.902545
C	0.285705	2.851710	0.191031
H	-0.308835	3.178283	-0.670309
H	-0.326772	3.070748	1.071687
H	1.196829	3.451374	0.232499
O	-1.723220	1.116429	0.284632
C	-2.968172	0.362990	0.244886
C	-2.725389	-1.047362	0.775071
C	-1.538586	-1.790327	0.195603
H	-1.285417	-2.661195	0.815622
H	-1.751114	-2.190956	-0.818230
H	-3.076089	-0.004726	1.487260
H	-3.662768	-1.607890	0.772860
C	-3.569664	0.582605	-1.128387
H	-4.557370	0.113601	-1.174812
H	-2.952632	0.146799	-1.943541
H	-3.696415	1.650934	-1.345976
C	1.225047	-2.856061	-0.160704
H	1.889977	-3.083073	-1.006273
H	1.682919	-3.275650	0.747298
H	0.300773	-3.405751	-0.336726

2,3-*cis* 17.2 kJ mol^{-1}

-1 1

C	0.835953	1.400176	0.014219
C	-0.427428	0.818233	-0.149760
C	-0.518427	-0.584198	-0.184716
C	0.638266	-1.379974	-0.128028
C	1.898106	-0.788384	0.007976
C	1.976937	0.598921	0.076265
O	3.235189	1.167630	0.205319
H	3.117473	2.118596	0.229834
C	3.146878	-1.620681	0.088545
H	4.007588	-1.000564	0.340821
H	3.362795	-2.119627	-0.864981
H	3.054795	-2.406610	0.846336
C	0.488906	-2.871077	-0.209904
H	0.377559	-3.322507	0.785721
H	-0.410821	-3.123916	-0.773359
H	1.354061	-3.339307	-0.687958
O	-1.683116	-1.262597	-0.243698
C	-2.915598	-0.462087	-0.193954
C	-2.747716	0.807738	-0.937576
C	-1.677410	1.651447	-0.307407

H -1.441920 2.506694 -0.954349
H -1.894353 2.115247 0.701612
H -3.657538 -1.103148 -0.686758
H -3.702321 1.323323 -1.091822
C -3.275331 -0.404183 1.306711
H -4.264212 0.053152 1.418260
H -2.553552 0.208713 1.856998
H -3.295070 -1.403399 1.765592
C 0.987321 2.894475 0.113839
H 1.653581 3.184757 0.939501
H 1.386130 3.338115 -0.810062
H 0.026477 3.367779 0.314372

2,3-*cis* 18.9 kJ mol⁻¹

-1 1
C 0.837930 1.396196 0.020592
C -0.428388 0.819065 -0.143443
C -0.526514 -0.587996 -0.182158
C 0.635506 -1.380628 -0.133336
C 1.896767 -0.792091 0.005473
C 1.978562 0.594193 0.077273
O 3.238385 1.162194 0.202561
H 3.117980 2.111041 0.265975
C 3.144243 -1.626702 0.084660
H 4.003706 -1.010280 0.350470
H 3.367152 -2.114721 -0.873021
H 3.045786 -2.421498 0.832304
C 0.486524 -2.871292 -0.223403
H 0.375823 -3.329648 0.769251
H -0.415643 -3.118941 -0.785674
H 1.350784 -3.337277 -0.705825
O -1.685027 -1.269193 -0.238954
C -2.946302 -0.430214 -0.191377
C -2.757517 0.832854 -0.874897
C -1.669708 1.672678 -0.308054
H -1.429911 2.493100 -0.997871
H -1.858114 2.187696 0.681718
H -3.684519 -1.057038 -0.704166
H -3.548307 1.252396 -1.484629
C -3.275493 -0.424513 1.316291
H -4.270288 0.009542 1.458370
H -2.555263 0.193530 1.863075
H -3.258979 -1.433715 1.752378
C 0.996231 2.890421 0.120159
H 1.643490 3.179738 0.961560
H 1.423400 3.327673 -0.794344
H 0.034987 3.372572 0.294894

2,3-*cis* −155.9 kJ mol⁻¹

-1 1
C 0.223308 1.321123 0.316549
C -0.562868 0.216364 0.649408
C -0.044487 -1.124798 0.594050
C 1.321672 -1.252750 0.169999
C 2.089963 -0.146151 -0.193200
C 1.536477 1.131705 -0.118336
O 2.336489 2.229235 -0.465124
H 1.774004 2.862470 -0.912935
C 3.507000 -0.326626 -0.662227
H 3.919526 0.614835 -1.027913
H 3.565806 -1.071479 -1.464614
H 4.153472 -0.688558 0.148040
C 1.893534 -2.637520 0.113247
H 2.827854 -2.727813 0.684084
H 1.151851 -3.324866 0.526718
H 2.119475 -2.955544 -0.915510
O -0.758379 -2.139500 0.900398
C -3.393263 0.110834 -1.001371
C -2.950662 0.834139 0.030634
C -1.997712 0.360325 1.098455
H -2.069552 1.037151 1.961255
H -2.287704 -0.638305 1.443591
H -4.070485 0.596943 -1.706261
H -3.290347 1.868930 0.096096
C -3.019625 -1.304971 -1.299430
H -3.916023 -1.930292 -1.408496
H -2.372172 -1.740011 -0.528414
H -2.479601 -1.363098 -2.253659
C -0.314397 2.725006 0.401916
H 0.443599 3.418084 0.785094
H -0.650491 3.103495 -0.575277
H -1.177724 2.780108 1.067849

Methyltrolox *m/z* 248 (−)

Figure S15

7.3 kJ mol⁻¹ (ΔF_{90K})

-1 2
C -1.546174 1.442853 -0.085435
C -2.689413 0.558122 0.063439
C -2.450952 -0.879034 0.036834
C -1.180992 -1.369530 -0.140745
C -0.098429 -0.464939 -0.310293
C -0.281742 0.937348 -0.280608
C 0.916878 1.827104 -0.447902
C 2.070079 1.085622 -1.107394
C 2.282975 -0.255327 -0.425697
O 1.076984 -1.035046 -0.554251
C 2.623291 -0.006729 1.099986
O 3.732892 0.538681 1.254555
O 1.755049 -0.341047 1.917837
C 3.359814 -1.080545 -1.093204
H 3.401684 -2.075848 -0.642264
H 4.319645 -0.587877 -0.934225
H 3.165837 -1.188015 -2.167062
H 2.996263 1.657487 -1.023547
H 1.857055 0.915605 -2.172201
H 1.236461 2.178528 0.541553
H 0.646972 2.714952 -1.029064
C -0.851755 -2.831268 -0.131098
H -0.061205 -3.024355 0.599783
H -0.462961 -3.154391 -1.103062
H -1.722658 -3.439264 0.114489
C -3.640171 -1.770115 0.229441
H -3.751765 -2.482848 -0.594929
H -3.559497 -2.351670 1.155238

H -4.538366 -1.154211 0.287711
O -3.846552 1.017633 0.212068
C -1.795722 2.916244 -0.018060
H -1.159233 3.391080 0.736465
H -2.842714 3.093963 0.229448
H -1.578620 3.406268 -0.975605

170.6 kJ mol^{-1}

-1 2
C 1.462006 -1.241140 -0.123517
C 0.090917 -0.962715 -0.079163
C -0.331856 0.372341 -0.091708
C 0.581383 1.434717 -0.165666
C 1.946170 1.150141 -0.220947
C 2.368538 -0.183005 -0.184081
O 3.729125 -0.459196 -0.229880
C 4.322748 -0.478077 1.048834
H 4.218470 0.491101 1.553942
H 3.872207 -1.250222 1.686533
H 5.384234 -0.698999 0.909835
C 2.947546 2.263555 -0.327983
H 3.937565 1.872869 -0.566386
H 2.660285 2.972792 -1.110389
H 3.016531 2.836368 0.605260
C 0.075649 2.847153 -0.183618
H 0.228098 3.309522 -1.167067
H 0.605100 3.466418 0.548239
H -0.990934 2.873270 0.039442
O -1.633257 0.725575 -0.002185
C -2.623375 -0.222239 -0.021889
C -4.017305 0.242709 0.280467
O -4.877993 -0.665945 0.155358
O -4.155846 1.437276 0.611683
C -2.268027 -1.536258 -0.598774
C -0.949828 -2.045390 -0.020151
H -1.104906 -2.358567 1.021483
H -0.608135 -2.932674 -0.565012
H -3.107170 -2.206017 -0.407557
H -2.157548 -1.454410 -1.695967
C 1.950800 -2.661105 -0.110787
H 3.038089 -2.697308 -0.180778
H 1.542380 -3.224895 -0.956509
H 1.637300 -3.185053 0.799013

0.0 kJ mol^{-1}

-1 2
C 0.599305 1.051159 -0.017950
C 1.993668 1.442979 0.109314
O 2.324012 2.652245 0.239123
C 2.991413 0.387883 0.087834
C 2.641202 -0.932050 -0.038236
C 1.253956 -1.332117 -0.161373
O 0.919121 -2.547181 -0.246477
C 0.261251 -0.274814 -0.168050
C -1.143342 -0.773267 -0.280015
H -1.095208 -1.780293 -0.702749
C -1.944534 -0.799451 1.022124
C -3.380722 -0.668430 0.540161
C -3.219937 0.101984 -0.763543
O -1.961101 0.027262 -1.189662
O -4.083933 0.691842 -1.361043
C -4.370303 -0.016727 1.486639
H -3.998504 0.958170 1.817002
H -5.330089 0.141976 0.988806
H -4.529886 -0.637457 2.373118
H -3.758008 -1.657143 0.240025
H -1.736756 -1.713686 1.580500
H -1.670857 0.054068 1.650664
C 3.673636 -2.018475 -0.050129
H 3.177494 -2.984056 -0.161093
H 4.261739 -2.026820 0.876496
H 4.386053 -1.887440 -0.874387
C 4.426549 0.800337 0.218638
H 5.016277 0.484068 -0.651060
H 4.896957 0.346375 1.100173
H 4.481650 1.886333 0.308082
C -0.402090 2.169516 0.000913
H 0.098972 3.075346 0.345933
H -0.808980 2.356955 -0.997705
H -1.255953 1.959097 0.652711

112.1 kJ mol^{-1}

-1 2
C -0.774702 1.204933 -0.656007
C -0.294595 -0.052160 -0.944374
C -1.034379 -1.248744 -0.601126
C -2.298818 -1.074768 0.083852
C -2.780115 0.175937 0.381892
C -2.049626 1.378271 0.020692
C 1.027273 -0.271717 -1.624819
O -0.580814 -2.395529 -0.882961
C -0.018697 2.457529 -0.996892
C -3.058731 -2.313650 0.450512
C -4.088520 0.355779 1.090987
O -2.500341 2.524263 0.290637
H 0.092527 2.582296 -2.081138
H 0.989802 2.461159 -0.567760
H -0.572806 3.313996 -0.609499
H -2.501012 -3.189094 0.113317
H -4.054300 -2.327444 -0.010840
H -3.213412 -2.386773 1.534671
H -4.093092 -0.151937 2.063770
H -4.920930 -0.065060 0.512390
H -4.270294 1.420626 1.244896
C 2.074927 -1.007029 -0.774781
C 2.541804 -0.274462 0.448264
H 1.448914 0.673843 -1.973947
H 0.861356 -0.906237 -2.503440
C 3.954442 -0.018801 0.624416
O 5.060660 -0.323614 0.953531
O 3.356092 1.031869 0.111708
H 2.943258 -1.244256 -1.399181
H 1.630486 -1.954806 -0.451804
C 1.611288 -0.095686 1.607662
H 1.282156 -1.084082 1.941724
H 0.719104 0.455659 1.303514

H 2.104163 0.413615 2.438348

188.8 kJ mol^{-1}

-1 2
C 0.397194 0.539442 -0.847361
C 1.594513 1.076280 -0.335846
C 2.634729 0.281580 0.199601
C 2.466689 -1.084507 0.259706
C 1.231842 -1.691987 -0.217131
C 0.192221 -0.829974 -0.765313
C -1.106822 -1.462366 -1.163920
C -2.167677 -1.114948 -0.149629
C -3.258962 -0.392983 -0.400705
C -4.238452 0.064397 0.678454
O -4.971442 1.013202 0.310554
O -4.179153 -0.506666 1.784005
H -3.456673 -0.024124 -1.408389
H -2.006513 -1.449075 0.874539
H -0.955907 -2.544035 -1.212893
H -1.420279 -1.111775 -2.153867
O 1.078972 -2.930653 -0.165906
C 3.519179 -1.982097 0.823111
H 3.159375 -3.010996 0.823622
H 4.442532 -1.933469 0.232842
H 3.781258 -1.695328 1.848008
C 3.891576 0.927860 0.704913
H 3.912717 1.988440 0.455814
H 4.776456 0.451914 0.270623
H 3.978118 0.824530 1.792963
O 1.784753 2.432921 -0.369679
C 1.120367 3.126247 0.677717
H 1.538851 2.846621 1.652130
H 1.284272 4.190369 0.501690
H 0.046399 2.916847 0.679755
C -0.624709 1.451465 -1.457654
H -0.184187 2.416953 -1.709010
H -1.035297 1.011005 -2.369343
H -1.476281 1.601250 -0.785935

Methyltrolox m/z 204 (−)

Figure S16

0.0 kJ mol^{-1} (ΔF_{90K})

-1 2
C -0.097562 1.138796 -0.436070
C -1.445452 1.402389 0.036581
C -2.294933 0.251820 0.284914
C -1.848049 -1.031485 0.083036
C -0.503878 -1.295661 -0.390888
C 0.337296 -0.145954 -0.653419
C 1.732051 -0.445457 -1.142919
C 2.677430 -0.634833 0.001779
C 3.780940 0.077126 0.226015
C 4.693593 -0.106435 1.396663
H 5.717147 -0.346364 1.081182
H 4.341972 -0.915942 2.042837
H 4.756160 0.805175 2.004454
H 4.046633 0.864673 -0.481282
H 2.391212 -1.417084 0.705606
H 1.681894 -1.382794 -1.709038
H 2.099865 0.347968 -1.801833
O -0.075511 -2.470943 -0.567325
C -2.728530 -2.215843 0.346840
H -2.175118 -3.129387 0.121571
H -3.637201 -2.189692 -0.268561
H -3.058383 -2.250095 1.393372
C -3.685324 0.520512 0.777723
H -3.864805 0.053507 1.754888
H -3.830418 1.598372 0.869108
H -4.442149 0.116771 0.092611
O -1.858997 2.580094 0.224922
C 0.785979 2.328320 -0.673013
H 1.728367 2.244567 -0.120294
H 0.257306 3.228286 -0.353792
H 1.042220 2.439064 -1.735063

48.7 kJ mol^{-1}

-1 2
C 1.257629 -1.343310 0.009239
C -0.097596 -1.141368 -0.023192
C -0.663211 0.153809 0.031412
O -2.376257 0.313131 -1.354758
C -2.181557 0.067759 -0.029219
C -2.986663 0.987139 0.893516
H -2.887937 2.031404 0.595158
H -2.671552 0.884369 1.938781
H -4.047994 0.718688 0.822175
C -2.433351 -1.414344 0.349619
C -1.191974 -2.173832 -0.117743
H -1.302714 -2.489809 -1.162041
H -0.991258 -3.071209 0.477137
H -2.533650 -1.505932 1.439413
H -3.362310 -1.759835 -0.115264
C 0.167745 1.295651 -0.032088
C 1.538079 1.128893 0.034486
C 2.143117 -0.195781 0.070357
O 3.388813 -0.351145 0.132325
C 2.482878 2.293981 0.057488
H 2.198903 3.040838 0.807646
H 3.485852 1.924496 0.280116
H 2.522929 2.809232 -0.912192
C -0.434357 2.665954 -0.177991
H -0.695750 3.104467 0.793913
H -1.340227 2.603570 -0.783804
H 0.263650 3.349618 -0.666861
C 1.899771 -2.693653 -0.010905
H 2.477206 -2.835323 -0.932701
H 1.161469 -3.496286 0.059462
H 2.619879 -2.789563 0.808645

92.2 kJ mol^{-1}

-1 2
C 1.107785 1.388862 -0.045893
C -0.206876 0.931465 -0.086854
C -0.466914 -0.440077 -0.077277
O -1.761853 -0.951454 -0.160135

C	-2.827831	-0.113454	-0.182982
C	-4.107372	-0.844220	0.009889
H	-4.136578	-1.745106	-0.610457
H	-4.958712	-0.211074	-0.257774
H	-4.250344	-1.166237	1.060083
C	-2.624485	1.232216	0.414703
C	-1.368481	1.887334	-0.146311
H	-1.134966	2.798215	0.414665
H	-1.552602	2.197736	-1.185155
H	-3.513439	1.848585	0.240908
H	-2.510137	1.139290	1.514645
C	0.574254	-1.371727	-0.022749
C	1.890740	-0.923012	0.028216
C	2.224598	0.480510	0.019230
O	3.426716	0.889496	0.066472
C	3.030609	-1.894233	0.095453
H	3.059170	-2.567705	-0.773092
H	3.959143	-1.319224	0.124301
H	2.984102	-2.533737	0.988639
C	0.276254	-2.845689	-0.017743
H	0.754710	-3.347606	-0.867742
H	-0.796457	-3.035142	-0.067689
H	0.668993	-3.324480	0.887903
C	1.422917	2.855008	-0.070821
H	2.509699	2.957768	-0.121259
H	1.072892	3.377583	0.832020
H	0.974439	3.368892	-0.931856

132.5 kJ mol^{-1}

-1 2

C	0.271502	-0.901475	-0.227600
C	-1.112559	-0.844906	-0.237606
C	-1.824027	0.369551	-0.132126
C	-1.128134	1.557871	-0.003007
C	0.315011	1.589612	0.025906
C	1.025005	0.297933	-0.068475
C	2.432544	0.411241	0.003782
C	3.468473	-0.540523	0.090536
C	4.800286	-0.222580	0.167700
H	5.132198	0.812043	0.154421
H	5.561115	-0.992376	0.246251
H	3.226268	-1.597762	0.117712
H	2.749963	1.452520	0.042578
O	0.934447	2.678848	0.132965
C	-1.843858	2.870383	0.104922
H	-1.108180	3.644882	0.330728
H	-2.611753	2.860794	0.889420
H	-2.349571	3.145651	-0.832039
C	-3.326301	0.366634	-0.180083
H	-3.763121	0.625016	0.794301
H	-3.707168	-0.611608	-0.478271
H	-3.696718	1.111496	-0.893011
O	-1.847776	-2.024172	-0.388971
C	-2.137472	-2.637768	0.842225
H	-1.219397	-2.914102	1.378679
H	-2.728060	-1.976313	1.491717
H	-2.716524	-3.542588	0.630379
C	0.930226	-2.234239	-0.440110
H	1.420105	-2.606895	0.468692
H	0.191753	-2.972938	-0.756157
H	1.701548	-2.165630	-1.213750

Figure S17

2,3-*trans* 5.0 kJ mol^{-1} (ΔF_{90K})

-1 2

C	-0.097562	1.138796	-0.436070
C	-1.445452	1.402389	0.036581
C	-2.294933	0.251820	0.284914
C	-1.848049	-1.031485	0.083036
C	-0.503878	-1.295661	-0.390888
C	0.337296	-0.145954	-0.653419
C	1.732051	-0.445457	-1.142919
C	2.677430	-0.634833	0.001779
C	3.780940	0.077126	0.226015
C	4.693593	-0.106435	1.396663
H	5.717147	-0.346364	1.081182
H	4.341972	-0.915942	2.042837
H	4.756160	0.805175	2.004454
H	4.046633	0.864673	-0.481282
H	2.391212	-1.417084	0.705606
H	1.681894	-1.382794	-1.709038
H	2.099865	0.347968	-1.801833
O	-0.075511	-2.470943	-0.567325
C	-2.728530	-2.215843	0.346840
H	-2.175118	-3.129387	0.121571
H	-3.637201	-2.189692	-0.268561
H	-3.058383	-2.250095	1.393372
C	-3.685324	0.520512	0.777723
H	-3.864805	0.053507	1.754888
H	-3.830418	1.598372	0.869108
H	-4.442149	0.116771	0.092611
O	-1.858997	2.580094	0.224922
C	0.785979	2.328320	-0.673013
H	1.728367	2.244567	-0.120294
H	0.257306	3.228286	-0.353792
H	1.042220	2.439064	-1.735063

2,3-*cis* 9.1 kJ mol^{-1}

-1 2

C	0.447160	1.385664	-0.254368
C	1.816235	1.086284	0.125278
C	2.196313	-0.311442	0.188497
C	1.299238	-1.311900	-0.096809
C	-0.068315	-1.016178	-0.472502
C	-0.449278	0.383385	-0.536826
C	-1.886499	0.655749	-0.913601
C	-2.737382	1.022275	0.268678
C	-3.583906	0.220949	0.919523
C	-3.849567	-1.220544	0.617374
H	-4.835704	-1.356904	0.152311
H	-3.856469	-1.806100	1.544081
H	-3.080148	-1.645310	-0.033160
H	-4.122576	0.644036	1.768619
H	-2.618201	2.039824	0.639695
H	-2.271596	-0.244898	-1.394904

H -1.932350 1.472846 -1.644537
O -0.893770 -1.936988 -0.737167
C 1.696681 -2.756090 -0.029922
H 2.528002 -2.978719 -0.711295
H 0.840566 -3.375967 -0.301733
H 2.031623 -3.036412 0.977256
C 3.609717 -0.621233 0.580247
H 4.142684 0.313487 0.763582
H 3.651852 -1.238336 1.487130
H 4.133724 -1.180704 -0.205586
O 2.635367 2.009540 0.387973
C 0.084690 2.840126 -0.293224
H 0.961550 3.426191 -0.577828
H -0.217778 3.199119 0.699427
H -0.738469 3.045150 -0.983571

3,4-*trans* 0.0 kJ mol^{-1}
-1 2
C 0.545949 1.363212 -0.041943
C 1.976148 1.170201 0.061015
C 2.464425 -0.198280 0.074551
C 1.606275 -1.261445 -0.005341
C 0.164908 -1.078935 -0.107816
C -0.335777 0.290694 -0.124410
C -1.764370 0.559294 -0.226886
C -2.796556 -0.297972 -0.316056
C -4.220324 0.152168 -0.402697
C -5.065348 -0.332880 0.776078
H -6.114306 -0.029619 0.676114
H -4.678068 0.069915 1.716963
H -5.034611 -1.424751 0.851439
H -4.260717 1.247729 -0.459553
H -4.673593 -0.224170 -1.332426
H -2.592844 -1.364461 -0.311805
H -2.026139 1.617405 -0.233356
O -0.595662 -2.077067 -0.177922
C 2.114018 -2.671879 0.009209
H 2.792594 -2.864158 -0.831910
H 2.678950 -2.887336 0.925217
H 1.267852 -3.357819 -0.055302
C 3.946172 -0.397864 0.179719
H 4.212193 -0.971538 1.076921
H 4.436512 0.576010 0.223825
H 4.338824 -0.957320 -0.679019
O 2.775304 2.145489 0.136130
C 0.081942 2.792083 -0.053194
H -0.458157 3.042073 -0.974652
H -0.589187 3.013281 0.785835
H 0.958784 3.436996 0.022669

3,4-*cis* 8.5 kJ mol^{-1}
-1 2
C 0.752925 1.443137 -0.101641
C 2.057728 0.862595 0.135930
C 2.147641 -0.586348 0.111348
C 1.049331 -1.368095 -0.138667
C -0.263564 -0.792080 -0.386255
C -0.361664 0.658617 -0.335373
C -1.641776 1.321461 -0.625060
C -2.854726 1.068230 -0.118971
C -3.213929 0.057228 0.920454
C -4.168074 -1.008505 0.386516
H -5.080025 -0.556661 -0.023118
H -3.660514 -1.567490 -0.402108
H -4.465660 -1.707055 1.176887
H -2.308517 -0.423360 1.295083
H -3.686632 0.579386 1.766902
H -3.685033 1.675948 -0.485870
H -1.578108 2.151264 -1.330919
O -1.251077 -1.525935 -0.650437
C 1.154587 -2.863256 -0.178572
H 1.505220 -3.268707 0.779390
H 1.869180 -3.196962 -0.941936
H 0.173884 -3.285222 -0.404333
C 3.494461 -1.193907 0.364842
H 4.215414 -0.397770 0.558330
H 3.473879 -1.875263 1.224937
H 3.839622 -1.782880 -0.494858
O 3.071538 1.581226 0.357653
C 0.662558 2.939834 -0.065521
H -0.157502 3.278040 0.576755
H 1.609318 3.343110 0.298232
H 0.478794 3.357820 -1.065278

1,2 15.0 kJ mol^{-1}
-1 2
C 0.035026 1.121977 -0.545412
C -1.305413 1.414142 -0.064945
C -2.156990 0.282799 0.253420
C -1.722124 -1.010830 0.100081
C -0.388707 -1.303661 -0.385853
C 0.465730 -0.175019 -0.699009
C 1.846700 -0.519989 -1.180355
C 2.761380 -1.022271 -0.047771
C 2.990991 -0.006472 1.018557
C 4.180600 0.406539 1.452657
H 4.274103 1.154889 2.234168
H 5.104094 0.008200 1.036809
H 2.087960 0.421940 1.453760
H 3.724164 -1.353378 -0.457652
H 2.263967 -1.900500 0.381761
H 2.313313 0.336476 -1.676130
H 1.761672 -1.334190 -1.907936
O 0.021659 -2.489641 -0.534469
C -2.606051 -2.176686 0.426660
H -2.061727 -3.102714 0.232432
H -2.921259 -2.163372 1.478071
H -3.523013 -2.170702 -0.176820
C -3.536723 0.583401 0.757495
H -3.702853 0.159546 1.756384
H -4.307865 0.156923 0.102825
H -3.672759 1.665214 0.804958
O -1.711439 2.601346 0.072667
C 0.904397 2.303958 -0.868780
H 1.113780 2.369536 -1.944528
H 1.870687 2.257392 -0.354988

H 0.381071 3.213768 -0.569168

Methyltrolox *m/z* 263 → 204 (−)

Figure S18

0.0 kJ mol⁻¹ (Δ*G*)

-1 2
C 1.107766 1.388846 -0.046090
C -0.206898 0.931458 -0.087027
C -0.466917 -0.440081 -0.077369
O -1.761886 -0.951480 -0.160571
C -2.827842 -0.113422 -0.182915
C -4.107419 -0.844145 0.009938
H -4.136453 -1.745189 -0.610175
H -4.958717 -0.211085 -0.258075
H -4.250618 -1.165860 1.060192
C -2.624361 1.232102 0.414986
C -1.368499 1.887359 -0.146248
H -1.134870 2.798139 0.414849
H -1.552823 2.197886 -1.185006
H -3.513390 1.848454 0.241526
H -2.509655 1.139025 1.514871
C 0.574270 -1.371732 -0.022629
C 1.890742 -0.923008 0.028086
C 2.224577 0.480527 0.019309
O 3.426668 0.889538 0.066879
C 3.030656 -1.894228 0.095123
H 3.053131 -2.574329 -0.768309
H 3.959547 -1.319393 0.114435
H 2.989805 -2.526780 0.993602
C 0.276279 -2.845727 -0.017415
H 0.746250 -3.346216 -0.873052
H -0.796862 -3.035049 -0.057491
H 0.677973 -3.325995 0.883417
C 1.422890 2.855015 -0.071019
H 2.509634 2.957752 -0.122323
H 1.073690 3.377386 0.832256
H 0.973638 3.369022 -0.931560

194.9 kJ mol⁻¹

-1 2
C 1.089117 1.398296 -0.021907
C -0.206199 0.961853 -0.273150
C -0.490306 -0.399409 -0.351471
O -1.802247 -0.828768 -0.644070
C -2.819162 -0.129406 -0.044392
C -3.670773 -0.924049 0.887843
H -3.979443 -1.867607 0.429384
H -4.566546 -0.363770 1.173400
H -3.108044 -1.158398 1.806470
C -2.596231 1.314873 0.207391
C -1.378118 1.894647 -0.460069
H -1.162709 2.882526 -0.048849
H -1.560203 2.044583 -1.536976
H -3.463233 0.827437 -0.661498
H -2.971608 1.729520 1.140858
C 0.509375 -1.357662 -0.198535
C 1.817533 -0.935559 0.042499
C 2.170024 0.458754 0.154689
O 3.360263 0.838178 0.390752
C 2.924736 -1.932256 0.210582
H 2.997008 -2.626119 -0.638456
H 3.862261 -1.378421 0.301799
H 2.801349 -2.549189 1.113310
C 0.182818 -2.822080 -0.290152
H 0.677131 -3.287992 -1.152269
H -0.892247 -2.978558 -0.392009
H 0.532333 -3.360540 0.598795
C 1.428897 2.857064 0.069351
H 2.513881 2.933869 0.177278
H 0.966293 3.345495 0.939784
H 1.119394 3.421485 -0.820558

34.5 kJ mol⁻¹

-1 2
C 0.917869 1.450896 -0.038315
C -0.337771 0.936223 -0.338744
C -0.535005 -0.437612 -0.460063
O -1.810589 -0.908053 -0.794442
C -2.832081 -0.358219 0.025615
C -2.757823 -0.938244 1.440872
H -2.870307 -2.026844 1.408129
H -3.544622 -0.517852 2.075483
H -1.785499 -0.696203 1.878664
C -2.772680 1.131334 0.013472
C -1.559248 1.806465 -0.522400
H -1.419065 2.780007 -0.042646
H -1.692226 2.025293 -1.600194
H -3.771392 -0.697550 -0.438388
H -3.605326 1.697721 0.422291
C 0.519402 -1.331165 -0.279318
C 1.791444 -0.833215 0.008250
C 2.051564 0.577995 0.151841
O 3.207891 1.028562 0.430707
C 2.953676 -1.762020 0.193901
H 3.106180 -2.420927 -0.672544
H 3.849047 -1.153201 0.341178
H 2.833271 -2.416433 1.070189
C 0.284889 -2.810761 -0.400362
H 0.859489 -3.240556 -1.230828
H -0.771741 -3.022541 -0.573070
H 0.605244 -3.338543 0.506400
C 1.160680 2.925379 0.096958
H 2.237072 3.069598 0.221326
H 0.658205 3.358700 0.974715
H 0.823750 3.492923 -0.781065

81.5 kJ mol⁻¹

-1 2
C 1.065764 1.404111 -0.055166
C -0.232064 0.930530 -0.006432
C -0.467271 -0.471297 -0.033465
O -1.673698 -1.008736 -0.162670
C -2.904336 0.046044 -0.352929
C -4.098979 -0.833600 -0.091111
H -3.974717 -1.812497 -0.563329

H -5.001702 -0.361823 -0.489350
H -4.231717 -0.981199 0.985009
C -2.706021 1.201429 0.451521
C -1.410100 1.883982 0.128206
H -1.162306 2.639741 0.882643
H -1.493365 2.442858 -0.823455
H -2.766375 0.239900 -1.426340
H -2.931199 1.078724 1.513688
C 0.623752 -1.375717 0.080757
C 1.921025 -0.907848 0.023511
C 2.204323 0.509676 -0.073870
O 3.389062 0.944328 -0.116061
C 3.092324 -1.842605 0.039381
H 3.014482 -2.611186 -0.739717
H 4.003612 -1.262892 -0.118658
H 3.182666 -2.369523 0.998997
C 0.328074 -2.845268 0.168127
H 0.406285 -3.333470 -0.812773
H -0.689031 -3.007470 0.528781
H 1.033898 -3.348751 0.835614
C 1.351065 2.873717 -0.027741
H 2.410180 3.032592 -0.238367
H 1.127889 3.314390 0.953655
H 0.743155 3.416946 -0.761291

–92.2 kJ mol^{-1}
-1 2
C 0.458674 1.374652 -0.287461
C -0.352902 0.330618 -0.660686
C 0.085544 -1.049090 -0.580250
O -0.665520 -1.998359 -0.936782
C -3.595058 -0.424422 0.266194
C -4.509242 -0.452705 1.448256
H -4.289663 -1.309849 2.097070
H -5.560724 -0.546242 1.147676
H -4.408744 0.456962 2.048321
C -2.734648 0.554038 -0.006139
C -1.763317 0.544311 -1.149308
H -1.832820 1.489298 -1.701196
H -2.017388 -0.277393 -1.824192
H -3.606169 -1.293765 -0.390394
H -2.674422 1.405696 0.674602
C 1.427227 -1.281226 -0.080288
C 2.242540 -0.240867 0.293302
C 1.804543 1.140026 0.204747
O 2.559373 2.094338 0.542347
C 3.628500 -0.487784 0.809623
H 4.248903 -1.009319 0.069096
H 4.096026 0.468521 1.050651
H 3.618285 -1.116201 1.709634
C 1.888635 -2.704810 0.009474
H 2.785314 -2.878944 -0.599409
H 1.090202 -3.361365 -0.341137
H 2.149069 -2.981347 1.039454
C 0.010710 2.805548 -0.373256
H 0.805857 3.444326 0.015656
H -0.903513 2.988323 0.203358
H -0.198276 3.103726 -1.408965

0.0 kJ mol^{-1}
-1 2
C 1.107765 1.388846 -0.046093
C -0.206898 0.931458 -0.087028
C -0.466917 -0.440082 -0.077368
O -1.761886 -0.951480 -0.160571
C -2.827841 -0.113422 -0.182914
C -4.107420 -0.844145 0.009937
H -4.136456 -1.745186 -0.610181
H -4.958718 -0.211082 -0.258072
H -4.250618 -1.165864 1.060189
C -2.624361 1.232101 0.414991
C -1.368499 1.887359 -0.146245
H -1.134870 2.798138 0.414854
H -1.552826 2.197888 -1.185002
H -3.513390 1.848454 0.241533
H -2.509651 1.139022 1.514875
C 0.574270 -1.371732 -0.022625
C 1.890743 -0.923008 0.028091
C 2.224577 0.480527 0.019312
O 3.426667 0.889541 0.066883
C 3.030656 -1.894228 0.095126
H 3.053180 -2.574268 -0.768355
H 3.959544 -1.319393 0.114521
H 2.989756 -2.526846 0.993556
C 0.276282 -2.845728 -0.017421
H 0.746204 -3.346200 -0.873094
H -0.796862 -3.035050 -0.057441
H 0.678028 -3.326012 0.883379
C 1.422890 2.855014 -0.071028
H 2.509629 2.957752 -0.122427
H 1.073780 3.377370 0.832292
H 0.973554 3.369037 -0.931515

187.6 kJ mol^{-1}
-1 2
C 1.058321 1.400370 -0.039767
C -0.244042 0.902912 0.002654
C -0.457206 -0.474830 0.033699
O -1.747365 -1.033981 0.079002
C -2.798736 -0.205308 -0.133109
C -4.105732 -0.875464 -0.290530
H -4.095372 -1.948056 -0.436449
H -3.439462 -0.265493 -1.274581
H -4.999061 -0.385846 0.083533
C -2.697928 1.160751 0.453403
C -1.419205 1.847457 -0.008780
H -1.209447 2.713350 0.629145
H -1.565870 2.244944 -1.023447
H -3.587106 1.744466 0.194207
H -2.689260 1.051646 1.552289
C 0.612758 -1.371559 0.037747
C 1.916539 -0.881724 0.009039
C 2.205507 0.530151 -0.033230
O 3.395021 0.977724 -0.063627
C 3.087786 -1.817150 0.017778
H 3.099938 -2.482207 -0.857879

H 3.997911 -1.212715 0.009359
H 3.100283 -2.465017 0.905798
C 0.363198 -2.854174 0.078384
H 0.844531 -3.357404 -0.768835
H -0.703262 -3.080109 0.053184
H 0.785690 -3.300939 0.987207
C 1.322490 2.875104 -0.100037
H 2.400997 3.011613 -0.211699
H 1.004613 3.399995 0.813454
H 0.811130 3.362603 -0.941796

49.6 kJ mol^{-1}

-1 2
C 1.044644 1.400150 -0.012949
C -0.256447 0.897875 0.050113
C -0.472216 -0.481348 0.069938
O -1.756743 -1.030061 0.156505
C -2.771366 -0.201992 -0.352587
C -4.056379 -0.920880 -0.219437
H -4.070714 -1.926279 0.182110
H -2.560725 0.001541 -1.425392
H -4.977585 -0.460876 -0.558937
C -2.743530 1.147407 0.368875
C -1.427071 1.850541 0.068793
H -1.251563 2.644245 0.804615
H -1.496161 2.361682 -0.903028
H -3.602095 1.758210 0.065818
H -2.841996 0.941951 1.441026
C 0.605528 -1.371430 0.045403
C 1.907717 -0.878086 0.003823
C 2.193145 0.534664 -0.035726
O 3.382582 0.984159 -0.089512
C 3.082091 -1.809828 -0.011553
H 3.124118 -2.416621 -0.928401
H 3.989954 -1.203852 0.039323
H 3.069627 -2.513412 0.832343
C 0.355316 -2.854143 0.075467
H 0.913223 -3.364017 -0.718407
H -0.706122 -3.074852 -0.046048
H 0.686479 -3.296499 1.024499
C 1.300926 2.876215 -0.067316
H 2.381017 3.020166 -0.151350
H 0.950382 3.401206 0.834034
H 0.811288 3.359748 -0.925215

65.6 kJ mol^{-1}

-1 2
C -1.018497 1.417324 -0.000984
C 0.257643 0.904592 -0.155047
C 0.458439 -0.490916 -0.235297
O 1.689480 -0.984598 -0.425751
C 2.806843 -0.210479 0.441194
C 4.019116 -0.961473 0.491976
H 4.675919 -0.982010 -0.374509
H 2.313913 -0.153635 1.417128
H 4.112205 -1.778818 1.199454
C 2.813161 1.128314 -0.263256
C 1.452843 1.827032 -0.174645
H 1.352419 2.551049 -0.994657
H 1.422251 2.432802 0.742221
H 3.590198 1.771244 0.164709
H 3.088279 0.938923 -1.307195
C -0.636448 -1.376229 -0.106033
C -1.920834 -0.875114 0.013040
C -2.173926 0.548508 0.076333
O -3.343719 1.010592 0.199165
C -3.107388 -1.787026 0.086266
H -3.123717 -2.366490 1.020039
H -4.015758 -1.182618 0.046233
H -3.117639 -2.512737 -0.736690
C -0.380456 -2.855191 -0.138656
H -0.906447 -3.364612 0.676282
H 0.689123 -3.054284 -0.057309
H -0.736415 -3.303748 -1.075510
C -1.259369 2.892197 0.089396
H -2.335810 3.066729 0.139954
H -0.849331 3.430406 -0.775548
H -0.792041 3.330617 0.982373

–80.4 kJ mol^{-1}

-1 2
C 0.116585 1.151091 -0.574510
C 0.598123 -0.125348 -0.741205
C -0.218733 -1.289853 -0.460044
O 0.237041 -2.458026 -0.620209
C 3.176446 0.156920 0.964792
C 2.593877 0.228929 2.158425
H 1.874143 -0.521668 2.473529
H 3.864731 0.951457 0.669646
H 2.785530 1.052860 2.839493
C 2.907021 -0.900855 -0.053311
C 1.997530 -0.413208 -1.201146
H 1.957888 -1.220249 -1.940522
H 2.442488 0.465634 -1.679066
H 3.858055 -1.247025 -0.481323
H 2.417936 -1.763819 0.406453
C -1.568909 -1.054544 0.009707
C -2.054823 0.219517 0.175040
C -1.241388 1.385976 -0.112102
O -1.694064 2.555320 0.036228
C -3.452632 0.460379 0.661188
H -3.617439 0.017830 1.652256
H -3.630587 1.535567 0.720042
H -4.197131 0.013226 -0.010405
C -2.413456 -2.257458 0.305041
H -2.746614 -2.271392 1.350998
H -1.831094 -3.159323 0.107157
H -3.319983 -2.277668 -0.313848
C 0.948202 2.369398 -0.855648
H 0.351018 3.256510 -0.636821
H 1.266914 2.413918 -1.904815
H 1.853277 2.390138 -0.238277

Methyltrolox *m/z* 189 (−)

Figure S19

0.0 kJ mol^{-1} (ΔF_{90K})

-1 1

```
C  1.884925   0.411261   0.000000
C  1.454993  -0.983108  -0.000001
C  0.122634  -1.337977  -0.000001
C -0.865381  -0.318921  -0.000001
C -0.466712   1.038515  -0.000002
C  0.848762   1.422165  -0.000002
C  1.296404   2.850054   0.000000
H  0.451190   3.544710  -0.000016
H  1.925479   3.060641  -0.873173
H  1.925450   3.060650   0.873193
C -1.674833   1.947631  -0.000001
C -2.873325   0.996200  -0.000001
C -2.299244  -0.424452   0.000001
O -3.030817  -1.416891   0.000004
H -1.670764   2.604610   0.877731
H -1.670765   2.604609  -0.877734
C -0.274619  -2.788635  -0.000001
H -1.359172  -2.894867   0.000001
H  0.134072  -3.303597  -0.878588
H  0.134076  -3.303598   0.878582
C  2.537536  -2.020468  -0.000000
H  2.481622  -2.674124  -0.880826
H  2.481619  -2.674125   0.880824
H  3.504872  -1.513799   0.000002
O  3.099571   0.730949   0.000003
H -3.517276   1.111418   0.878429
H -3.517274   1.111416  -0.878432
```

163.1 kJ mol^{-1}

-1 1

```
C  1.697534   0.867951   0.000142
C  1.729272  -0.573268   0.000019
C  0.565803  -1.338717   0.000040
C -0.676432  -0.701524   0.000100
C -0.773747   0.689238   0.000108
C  0.384700   1.462581   0.000089
C  0.315933   2.961140   0.000065
H  1.341629   3.337946   0.000267
H -0.203605   3.360301  -0.882658
H -0.203979   3.360314   0.882557
C -2.124797   1.361833   0.000159
C -3.238189   0.365941  -0.000219
C -3.011668  -0.949766  -0.000201
O -1.806899  -1.536027   0.000089
H -3.808085  -1.688009  -0.000434
H -4.270026   0.703875  -0.000502
H -2.207324   2.030030   0.871900
H -2.207152   2.030505  -0.871223
C  0.651503  -2.840388  -0.000040
H -0.338432  -3.297564   0.000383
H  1.197979  -3.203637  -0.879158
H  1.198781  -3.203678   0.878554
C  3.078555  -1.226224  -0.000113
H  3.832312  -0.435291  -0.000384
H  3.236614  -1.863481   0.881886
H  3.236272  -1.863845  -0.881905
O  2.757426   1.568497  -0.000111
```

174.0 kJ mol^{-1}

-1 1

```
C  1.723314   0.833518   0.037598
C  0.437981   1.476285  -0.014215
C -0.743203   0.729518  -0.081321
C -0.701711  -0.674644  -0.079745
C  0.515540  -1.346646  -0.052046
C  1.701961  -0.609318   0.010535
C  3.027646  -1.305632   0.062125
H  3.807061  -0.540394   0.083312
H  3.190846  -1.958516  -0.806811
H  3.131456  -1.938533   0.955590
C  0.549933  -2.848690  -0.095467
H  1.026925  -3.209870  -1.015583
H  1.131920  -3.256505   0.739555
H -0.459195  -3.261387  -0.052773
O -1.879094  -1.401361  -0.239921
C -2.978202  -0.812197   0.418570
C -3.164718   0.614821   0.000698
C -2.056715   1.339971  -0.210480
H -2.121287   2.384836  -0.501123
H -4.164816   1.025317  -0.106649
H -2.818005  -0.873041   1.514049
H -3.847612  -1.427965   0.168830
C  0.418966   2.975393   0.015058
H  0.037173   3.411581  -0.919741
H  1.449883   3.312561   0.149117
H -0.195828   3.372964   0.833223
O  2.809936   1.491946   0.101315
```

188.3 kJ mol^{-1}

-1 1

```
C -1.998208  -0.148176   0.032776
C -1.444217   1.240654  -0.013612
O -2.265479   2.195540  -0.008721
C -0.043281   1.383608  -0.044084
C  0.837078   0.268918  -0.036013
C  2.214952   0.448875   0.016777
C  3.267610  -0.513498   0.065780
C  4.582023  -0.189475   0.114063
H  5.350644  -0.954226   0.159133
H  4.912818   0.847200   0.105865
H  2.983927  -1.559589   0.061057
H  2.553116   1.482592   0.048393
C  0.254169  -1.109695  -0.081589
O  0.939318  -2.124028  -0.212986
C -1.208798  -1.245419   0.023540
C -1.767577  -2.635801   0.058588
H -2.471048  -2.761885   0.889456
H -0.955755  -3.357260   0.160489
H -2.316783  -2.872695  -0.862170
C  0.500579   2.777953  -0.047656
H -0.336041   3.477872  -0.088289
H  1.092118   2.997799   0.853875
H  1.158396   2.964841  -0.908848
C -3.488185  -0.266984   0.085365
```

H -3.810908 -0.730506 1.026208
H -3.863929 -0.902210 -0.725347
H -3.924134 0.730216 0.010219

201.2 kJ mol^{-1}
-1 1
C -1.855993 -0.726601 -0.273838
O -2.931141 -1.340409 -0.453687
C -1.749205 0.669458 -0.052931
C -0.484296 1.299591 0.079264
C 0.667265 0.551416 -0.000544
C 0.706292 -0.831003 -0.204141
C 2.029699 -1.205571 -0.269004
C 2.822229 -0.019764 -0.099246
C 4.167409 0.178940 -0.081689
H 4.834794 -0.662904 -0.210554
H 4.583796 1.168011 0.058069
H 2.440153 -2.194101 -0.420875
C -0.577345 -1.593333 -0.259944
C -0.677426 -2.562749 0.924920
H -0.626125 -2.006510 1.866328
H -1.632998 -3.090486 0.878825
H 0.146628 -3.283184 0.912450
H -0.615712 -2.186097 -1.182355
O 1.961474 1.043346 0.065167
C -0.393127 2.782452 0.282862
H 0.646990 3.107117 0.341292
H -0.905470 3.082438 1.204615
H -0.883085 3.320068 -0.537234
C -3.014444 1.472911 -0.011426
H -3.859310 0.784443 -0.087346
H -3.095470 2.193915 -0.839512
H -3.123205 2.049312 0.918768

210.1 kJ mol^{-1}
-1 1
C 0.993683 -0.622697 0.234206
C 2.268841 -0.891921 -0.528416
C 2.961536 0.233029 -0.717460
C 2.234964 1.442482 -0.183361
C 0.891456 0.873688 0.165307
C -0.301517 1.549933 0.070177
O -0.466544 2.804585 -0.066229
C -1.519872 0.668131 0.045054
C -1.498100 -0.688072 -0.122098
C -0.227877 -1.399799 -0.212269
O -0.147692 -2.589728 -0.514658
C -2.751632 -1.495034 -0.295220
H -3.307887 -1.202118 -1.195295
H -2.494086 -2.552484 -0.384662
H -3.434182 -1.366220 0.553916
C -2.826705 1.394047 0.084864
H -3.371691 1.276203 -0.860489
H -2.624656 2.455502 0.242151
H -3.474877 0.995675 0.874886
H 2.792721 1.884936 0.663835
H 2.133243 2.251705 -0.919909
H 3.944843 0.280185 -1.180182
H 2.573953 -1.895714 -0.806307
C 1.187353 -1.101222 1.706472
H 0.300179 -0.852808 2.297026
H 1.353537 -2.184641 1.743702
H 2.050007 -0.584471 2.134883

282.9 kJ mol^{-1}
-1 1
C 0.093194 1.552285 0.082996
C -0.961964 0.727205 0.143356
C -0.854495 -0.781202 0.290399
C 0.520459 -1.297348 -0.067663
C 1.599057 -0.485135 -0.140453
C 1.447092 0.975800 -0.013710
C -2.411860 1.058587 -0.076318
O -1.107066 -1.194189 1.562292
C 0.011585 3.048686 0.047128
C 0.618239 -2.787294 -0.125112
C 3.007149 -0.994889 -0.285536
O 2.430632 1.723707 -0.032712
H -1.024573 3.391536 0.094911
H 0.480295 3.451303 -0.857757
H 0.558922 3.481868 0.891418
H -0.095134 -3.190505 0.599833
H 1.618788 -3.160532 0.105504
H 0.343537 -3.152534 -1.124652
H 3.099556 -1.715550 -1.104721
H 3.354083 -1.491818 0.629994
H 3.676147 -0.154220 -0.479101
C -2.896354 -0.207576 -0.748384
C -2.014396 -1.201597 -0.616395
H -2.548918 1.960146 -0.684367
H -2.940869 1.215201 0.875070
H -3.885320 -0.287000 -1.193104
H -2.171284 -2.229167 -0.931515

Methyltrolox *m/z* 204 → 189 (−)
Figure S20
0.0 kJ mol^{-1} (Δ*G*)
-1 2
C -1.107765 1.388846 -0.046090
C 0.206898 0.931458 -0.087027
C 0.466917 -0.440082 -0.077368
O 1.761886 -0.951479 -0.160573
C 2.827842 -0.113422 -0.182915
C 4.107419 -0.844145 0.009938
H 4.250617 -1.165864 1.060191
H 4.958718 -0.211083 -0.258070
H 4.136455 -1.745187 -0.610178
C 2.624361 1.232101 0.414988
C 1.368499 1.887358 -0.146247
H 1.552825 2.197885 -1.185006
H 1.134871 2.798140 0.414849
H 2.509653 1.139023 1.514872
H 3.513390 1.848454 0.241529
C -0.574270 -1.371732 -0.022625
C -1.890742 -0.923008 0.028090

C -2.224577 0.480527 0.019310
O -3.426668 0.889539 0.066878
C -3.030656 -1.894228 0.095126
H -2.989768 -2.526829 0.993568
H -3.959544 -1.319394 0.114499
H -3.053167 -2.574284 -0.768342
C -0.276281 -2.845727 -0.017418
H -0.678018 -3.326008 0.883387
H 0.796862 -3.035050 -0.057448
H -0.746211 -3.346203 -0.873085
C -1.422890 2.855015 -0.071022
H -2.509632 2.957752 -0.122357
H -0.973609 3.369026 -0.931545
H -1.073720 3.377381 0.832268

75.4 kJ mol^{-1}
-1 2
C 1.100627 -1.390183 -0.065456
C -0.200140 -1.082629 -0.379969
C -0.625437 0.279332 -0.446999
O -1.906666 0.510946 -1.003023
C -2.311316 0.110908 0.287512
C -3.097725 1.093060 1.067440
H -2.600140 2.067855 1.085902
H -3.222681 0.753044 2.100984
H -4.111252 1.253380 0.646731
C -2.511988 -1.365418 0.371110
C -1.378654 -2.014433 -0.460868
H -1.717155 -2.097308 -1.500534
H -1.149480 -3.022885 -0.110692
H -2.449937 -1.679764 1.421392
H -3.500379 -1.691107 -0.005833
C 0.301929 1.332283 -0.281056
C 1.618374 1.020305 0.037606
C 2.082408 -0.340821 0.145750
O 3.285698 -0.631911 0.422242
C 2.632751 2.103200 0.252811
H 2.315725 2.822734 1.019659
H 3.569680 1.636450 0.565309
H 2.824895 2.680834 -0.663183
C -0.143503 2.756829 -0.439860
H -0.134368 3.295068 0.518678
H -1.155873 2.800488 -0.846288
H 0.521267 3.310876 -1.113145
C 1.610323 -2.788997 0.096200
H 2.424053 -2.978624 -0.613281
H 0.832831 -3.542519 -0.054221
H 2.054658 -2.921429 1.089445

–42.2 kJ mol^{-1}
-1 2
C 1.257629 -1.343310 0.009239
C -0.097596 -1.141368 -0.023192
C -0.663211 0.153809 0.031412
O -2.376257 0.313131 -1.354758
C -2.181557 0.067759 -0.029219
C -2.986663 0.987139 0.893516
H -2.887937 2.031404 0.595158
H -2.671552 0.884369 1.938781
H -4.047994 0.718688 0.822175
C -2.433351 -1.414344 0.349619
C -1.191974 -2.173832 -0.117743
H -1.302714 -2.489809 -1.162041
H -0.991258 -3.071209 0.477137
H -2.533650 -1.505932 1.439413
H -3.362310 -1.759835 -0.115264
C 0.167745 1.295651 -0.032088
C 1.538079 1.128893 0.034486
C 2.143117 -0.195781 0.070357
O 3.388813 -0.351145 0.132325
C 2.482878 2.293981 0.057488
H 2.198903 3.040838 0.807646
H 3.485852 1.924496 0.280116
H 2.522929 2.809232 -0.912192
C -0.434357 2.665954 -0.177991
H -0.695750 3.104467 0.793913
H -1.340227 2.603570 -0.783804
H 0.263650 3.349618 -0.666861
C 1.899771 -2.693653 -0.010905
H 2.477206 -2.835323 -0.932701
H 1.161469 -3.496286 0.059462
H 2.619879 -2.789563 0.808645

3.9 kJ mol^{-1}
-1 2
C 1.126426 -1.397609 -0.037700
C -0.204648 -1.094239 -0.131994
C -0.671647 0.241488 -0.191982
O -2.881637 1.223475 -0.639772
C -2.147071 0.269901 -0.298166
C -2.231298 0.241062 1.774865
H -1.918332 1.256436 1.998732
H -1.627587 -0.531791 2.246629
H -3.306883 0.086786 1.818316
C -2.560726 -1.191438 -0.572254
C -1.369865 -2.057067 -0.162573
H -1.195561 -2.893147 -0.848202
H -1.520963 -2.493169 0.833721
H -3.498793 -1.443410 -0.072190
H -2.736950 -1.252976 -1.653609
C 0.241493 1.318395 -0.137844
C 1.592726 1.045752 -0.039699
C 2.097967 -0.320123 0.011725
O 3.326149 -0.568045 0.097335
C 2.606534 2.146917 0.010440
H 2.411517 2.841901 0.836963
H 3.598865 1.710413 0.137501
H 2.602995 2.745187 -0.910484
C -0.255088 2.737269 -0.179148
H -0.036132 3.253967 0.764787
H -1.331488 2.756500 -0.355660
H 0.248606 3.306294 -0.969743
C 1.659084 -2.793938 0.019749
H 2.423185 -2.947415 -0.749979
H 0.867536 -3.537213 -0.106858
H 2.160574 -2.980031 0.977055

−66.8 kJ mol−1

-1 2

C -0.903080 1.432446 -0.214242
C 0.417336 1.069019 -0.275893
C 0.834219 -0.281200 -0.334305
O 3.013881 -1.347942 -0.377402
C 2.269927 -0.365442 -0.349863
C 0.382706 -0.261164 3.040637
H 0.209117 -0.070430 4.092739
H -0.292317 0.126915 2.288196
H 1.229870 -0.858457 2.730199
C 2.823311 1.061999 -0.312548
C 1.611576 1.995228 -0.269422
H 1.584530 2.676498 -1.128067
H 1.610029 2.625181 0.627569
H 3.471829 1.158389 0.564614
H 3.458095 1.214479 -1.191911
C -0.137363 -1.314974 -0.323118
C -1.474179 -0.981102 -0.261384
C -1.924615 0.406187 -0.212159
O -3.142491 0.707018 -0.160699
C -2.539465 -2.035371 -0.234575
H -2.427867 -2.708400 0.625711
H -3.513288 -1.545197 -0.174347
H -2.517650 -2.665724 -1.133628
C 0.280760 -2.758824 -0.359803
H -0.066442 -3.283787 0.539167
H 1.364869 -2.848621 -0.422929
H -0.172852 -3.274365 -1.215374
C -1.369800 2.852463 -0.145688
H -2.017921 3.090805 -0.997595
H -0.534160 3.558201 -0.132257
H -1.984961 3.016318 0.746895

−81.6 kJ mol−1 (separated products)

-1 1

C 0.848762 -1.422165 0.000002
C -0.466713 -1.038514 -0.000003
C -0.865381 0.318921 -0.000004
O -3.030816 1.416892 -0.000001
C -2.299244 0.424453 0.000000
C -2.873325 -0.996200 0.000013
C -1.674834 -1.947630 -0.000010
H -1.670770 -2.604592 -0.877755
H -1.670760 -2.604626 0.877710
H -3.517253 -1.111414 0.878460
H -3.517297 -1.111419 -0.878401
C 0.122635 1.337977 -0.000001
C 1.454993 0.983107 0.000001
C 1.884925 -0.411262 0.000002
O 3.099571 -0.730950 0.000003
C 2.537536 2.020468 -0.000000
H 2.481704 2.674037 0.880896
H 3.504873 1.513799 -0.000128
H 2.481538 2.674211 -0.880754
C -0.274617 2.788635 -0.000001
H 0.134176 3.303626 0.878520
H -1.359170 2.894868 0.000106
H 0.133977 3.303569 -0.878650
C 1.296402 -2.850055 0.000000
H 1.925327 -3.060697 -0.873269
H 0.451187 -3.544710 0.000162
H 1.925597 -3.060596 0.873097

-1 2

C 0.000001 -0.000001 -0.000001
H -0.410043 1.000661 0.000003
H 1.071620 -0.145226 0.000003
H -0.661586 -0.855430 0.000003
